# Focused Screening Identifies Different Sensitivities of Human TET Oxygenases to the Oncometabolite 2-Hydroxyglutarate

**DOI:** 10.1021/acs.jmedchem.3c01820

**Published:** 2024-01-31

**Authors:** Roman Belle, Hilal Saraç, Eidarus Salah, Bhaskar Bhushan, Aleksandra Szykowska, Grace Roper, Anthony Tumber, Skirmantas Kriaucionis, Nicola Burgess-Brown, Christopher J. Schofield, Tom Brown, Akane Kawamura

**Affiliations:** † Chemistry Research Laboratory, Department of Chemistry, University of Oxford, 12 Mansfield Road, OX1 3TA Oxford, United Kingdom; ‡ Chemistry − School of Natural and Environmental Sciences, Bedson Building, Newcastle University, NE1 7RU Newcastle upon Tyne, United Kingdom; § Radcliffe Department of Medicine, Division of Cardiovascular Medicine, University of Oxford, Wellcome Trust Centre for Human Genetics, Roosevelt Drive, OX3 7BN Oxford, United Kingdom; ∥ Centre for Medicines Discovery, University of Oxford, Old Road Campus Research Building, Roosevelt Drive, OX3 7DQ Oxford, United Kingdom; ⊥ Ludwig Institute for Cancer Research, Nuffield Department of Medicine, University of Oxford, Old Road Campus Research Building, Roosevelt Drive, OX3 7DQ Oxford, United Kingdom; # Ineos Oxford Institute for Antimicrobial Research, University of Oxford, 12 Mansfield Road, OX1 3TA Oxford, United Kingdom

## Abstract

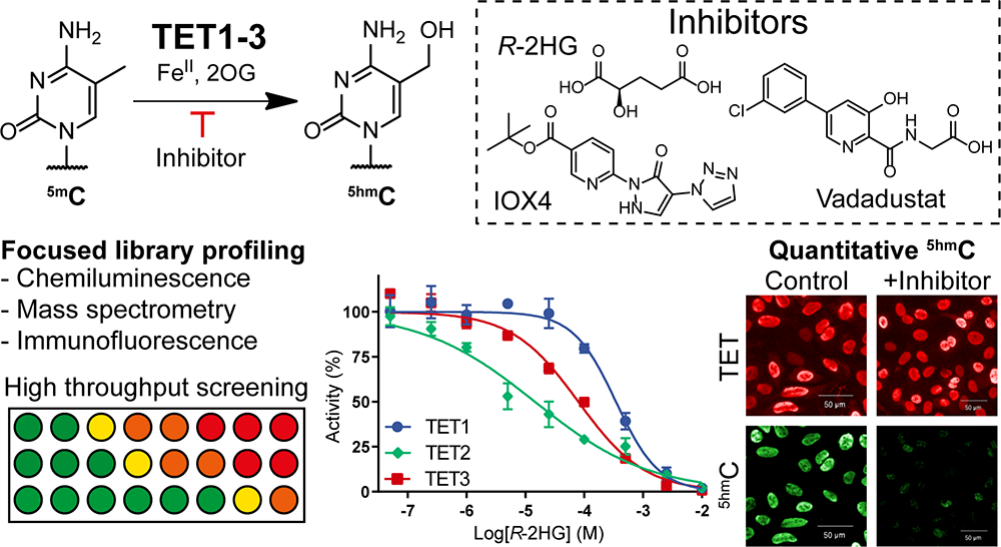

Ten-eleven translocation enzymes (TETs) are Fe(II)/2-oxoglutarate (2OG) oxygenases that catalyze the sequential oxidation of 5-methylcytosine to 5-hydroxymethylcytosine, 5-formylcytosine, and 5-carboxylcytosine in eukaryotic DNA. Despite their roles in epigenetic regulation, there is a lack of reported TET inhibitors. The extent to which 2OG oxygenase inhibitors, including clinically used inhibitors and oncometabolites, modulate DNA modifications via TETs has been unclear. Here, we report studies on human TET1–3 inhibition by a set of 2OG oxygenase-focused inhibitors, employing both enzyme-based and cellular assays. Most inhibitors manifested similar potencies for TET1–3 and caused increases in cellular ^5hm^C levels. (*R*)-2-Hydroxyglutarate, an oncometabolite elevated in isocitrate dehydrogenase mutant cancer cells, showed different degrees of inhibition, with TET1 being less potently inhibited than TET3 and TET2, potentially reflecting the proposed role of *TET2* mutations in tumorigenesis. The results highlight the tractability of TETs as drug targets and provide starting points for selective inhibitor design.

## Introduction

**Figure 1:**
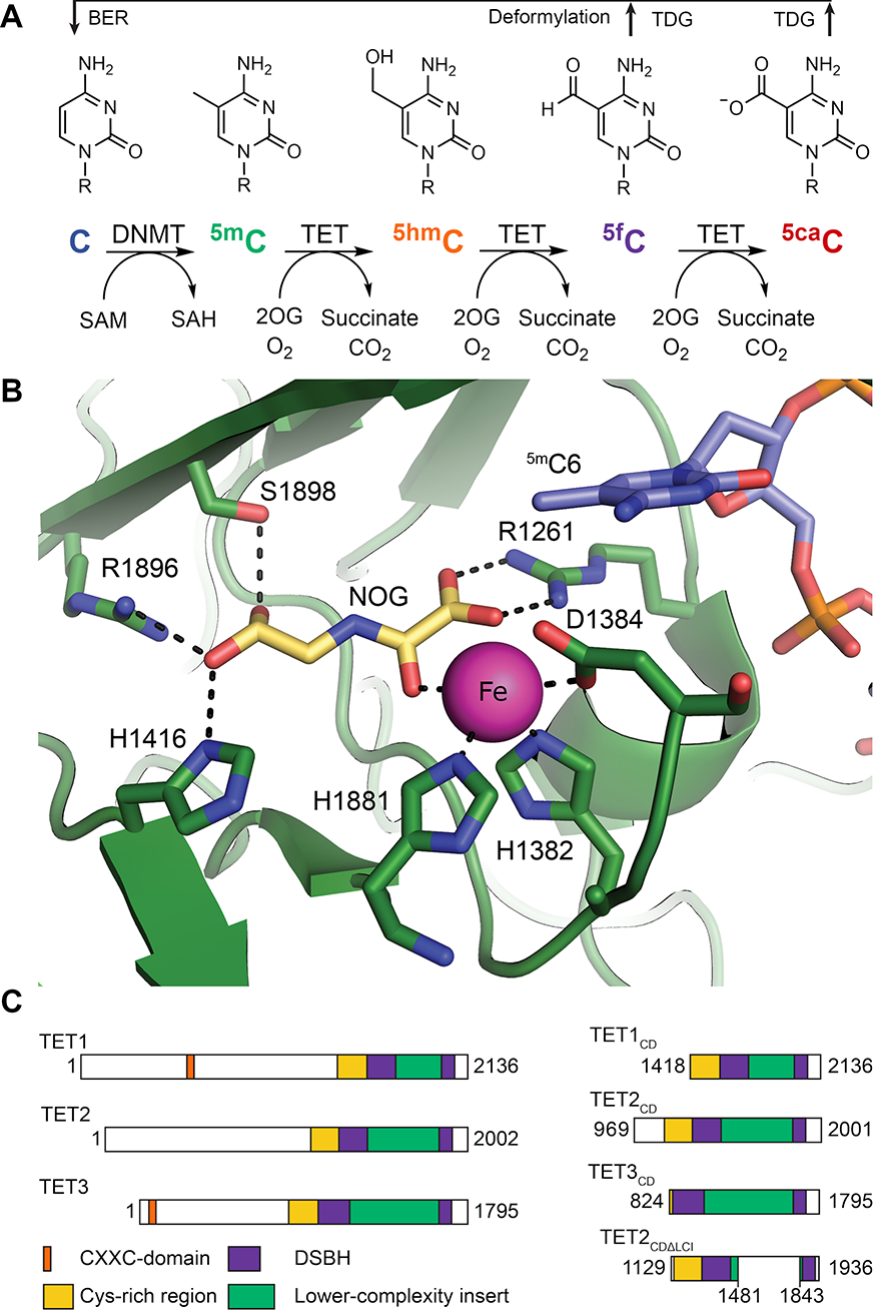
2OG oxygenase catalytic domains of TETs catalyze sequential oxidation of ^5m^C to ^5hm^C, ^5f^C, and then ^5ca^C. (A) Schematic representation of cytosine methylation, oxidation, and demethylation cycle. Cytosine is methylated by DNMTs to give ^5m^C, which undergoes sequential oxidation to ^5hm^C, ^5f^C, and ^5ca^C, as catalyzed by TETs. ^5f^C and ^5ca^C can be restored to C by the TDG-BER mechanism. (B) View of the active site of TET2 (green) complexed with ^5m^C-containing DNA (blue). ^5m^C is positioned near Fe(II) (pink) and 2OG binding sites (cofactor mimic NOG, yellow) (the figure was derived from PDB: 4NM6(19)). Dark blue represents nitrogen; red as oxygen; and orange as phosphorus. (C) TET domain structures (left) and TET constructs used in this study (right).

Eukaryotic transcription is regulated by an array of epigenetic proteins that add, recognize, or remove covalent modifications on chromatin. DNA methyl transferases (DNMTs) introduce methyl groups on cytosine to produce 5-methylcytosine (^5m^C), a relatively stable epigenetic modification that is associated with repression or enhancement of transcription, depending on its genomic location.^1^ The ten-eleven translocation enzymes (TETs) catalyze the sequential oxidation of ^5m^C to give 5-hydroxymethylcytosine (^5hm^C), 5-formylcytosine (^5f^C), and 5-carboxylcytosine (^5ca^C) (collectively 5-oxidizedcytosines, ^5ox^C)^2−4^ (Figure 1A). The presence of ^5m^C at CpG islands in promotor regions is typically associated with transcriptional repression,^5^ whereas the roles of ^5hm^C appear to be context-dependent.^6,7^^5ox^C can be intermediates in demethylation of ^5m^C, either via passive dilution as a result of cell division,^8,9^ via active thymidine DNA glycosylase–base excision repair (TDG-BER)-mediated mechanism^10^ or putatively via direct deformylation of ^5f^C^11^ (Figure 1A). ^5ox^C can have distinct and/or overlapping functions^12,13^ and are recognized by “reader” modules that bind to one or a combination of ^5ox^C marks to elicit biological responses.^14^ There is also evidence that ^5f^C and ^5hm^C may contribute to nucleosome stabilization and modulation of DNA stability.^15−18^

TET-mediated oxidation of ^5m^C is catalyzed by Fe(II) containing oxygenase domains that use O_2_ and 2-oxoglutarate (2OG) as cosubstrates (Figure 1A,B).^4^ Three human TET isozymes have been identified: TET1–3 (Figure 1C). Of the ∼60–70 human 2OG oxygenases, several subfamilies are involved in transcriptional regulation, including the JmjC histone lysine demethylases (JmjC-KDMs) and the hypoxia-inducible factor (HIF) prolyl hydroxylase domains (PHDs), the latter of which act as O_2_ sensors in responses to hypoxic stress.^20^ All characterized 2OG oxygenases contain a distorted double-stranded ß-helix (DSBH) core fold that supports Fe(II) binding by a conserved HXD/E···H triad (Figure 1B).^21^ In the TETs, two DSBH ß-strands (ß12 and ß13) are split by a characteristic low complexity insert (LCI) (Figure 1C).^19^ A cysteine-rich domain, situated adjacent to the oxygenase domain, is proposed to stabilize the DSBH fold and is necessary for TET1–3 catalysis.^19^ TET1 and TET3 contain a CXXC-domain that preferentially binds to nonmethylated DNA.^22^ TET2 lacks this domain (Figure 1C) but binds to several DNA-binding factors, including IDAX (CXXC4) which is reported to enable DNA binding in a similar manner as the CXXC-domain in TET1 and TET3.^23^ The TETs catalyze the oxidation of ^5m/ox^C substrates in DNA and RNA and thymidine in DNA^24^ with varying efficiencies depending on the context of the substrate base, including whether it is in single- or double-stranded DNA and the presence of other modifications in RNA.^25−27^

*TET* mutations are linked to diseases, inter alia hematopoietic malignancies such as chronic lymphocytic leukemia (CLL), acute myeloid leukemia (AML), and chronic myelomonocytic leukemia (CMML).^28−30^*TET2* is among the most frequently mutated genes in myeloid neoplasms and inactivating *TET2* mutants are linked to DNA hypermethylation, tumor progression, and poor patient outcome. In AML and myelodysplastic syndrome (MDS), *TET2* mutations are mostly mutually exclusive with those in isocitrate dehydrogenase (*IDH1/2*),^31^ which catalyzes decarboxylation of isocitrate to give 2OG. Mutant *IDHs* catalyze the production of the oncometabolite (2*R*)-hydroxyglutarate (*R*-2HG), which can accumulate to high levels (up to 30 mM).^32,33^ Elevated 2HG levels are proposed to inhibit 2OG oxygenases, including the TETs, in cells.^34^ The mutually exclusive nature of *IDH* and *TET2* mutations in AML is proposed to be due to functional redundancy^31^ in tumorigenesis and/or the synthetic lethality of TET1/3 inhibition by *R-*2HG in *TET2* mutant cells, which are reliant on TET1/3 for survival.^35^ TET inhibition is thus a potential strategy for treatment of AML-bearing *TET2* mutants. Genetic studies have also identified TET2 inhibition as a potential way to improve cancer immunotherapy.^36^

Potent and selective inhibitors will aid in functional studies and evaluation of the TETs as therapeutic targets. Some TET inhibitors are reported,^37−40^ including metal-chelating 2OG cofactor mimics, such as TCA cycle–related metabolites, including (*S*)- and (*R*)-2HG,^34,41−43^ IOX1,^37^ 2,4-PDCA,^41^ and TETi76;^35^ however, knowledge of their selectivity, potency, and cell activities across TET1–3 is limited. The development of TET inhibitors has likely been limited to date by a paucity of assays with sufficient sensitivity and throughput.^38^ Here, we report on the development of robust TET assays, both with isolated enzymes and in cells, which were used to screen a set of 2OG oxygenase–focused inhibitors and related compounds. We observed that several known 2OG oxygenase inhibitors also inhibit TET1–3, including some clinically used PHD inhibitors. Unexpectedly, *R-*2HG and *S*-2HG had different inhibition potencies across the TET1–3, as manifested in studies with isolated enzymes and in cells.

## Results

### Quantitative Assay for the Detection of ^5hm^C by TET-Mediated ^5m^C Oxidation

To develop a quantitative assay for kinetic studies and inhibitor screening of isolated TET1–3, the catalytic domain (CD) of recombinant human TET2_CD_ (Q969-I2002) was produced in insect cells (Sf9), and TET3_CD_ (E824-I1795) was produced in mammalian cells. TET1_CD_ (E1418-V2136), which was produced in insect (Sf9) cells, was from a commercial source (Figures 1C and S1). To monitor TET activity, an AlphaScreen assay was developed,^37^ wherein the biotinylated ^5hm^C product from a quenched TET reaction was quantitated by a ^5hm^C-antibody, using streptavidin conjugated donor and Protein-A acceptor bead pairs (Figure S2A).^37^ A standard curve with 32-base single-stranded 5′-biotinylated ^5m^C- **1**, ^5hm^C- **2,** and ^5m^C **1**/^5hm^C **2**-DNA mixture demonstrated the assay is sensitive and selective for ^5hm^C relative to ^5m^C (>10 S/N at ≥0.1 nM) with a linear range up to 1.5 nM (*R*^2^ = 0.98) (Figure S2B). Time course assays with recombinant TETs showed an increase in the ^5hm^C signal in an enzyme concentration–dependent manner; assay saturation was observed at higher enzyme concentrations, possibly due to further oxidation of ^5hm^C (Figure S3A–C). While TET1_CD_ (0.005 μM min^–1^ μM^–1^) and TET3_CD_ (0.016 μM min^–1^ μM^–1^) had comparable activities, the specific activity of TET2_CD_ was >10-fold higher (0.21 μM min^–1^ μM^–1^); thus, 10 nM protein was used for TET1/3_CD_ assays, while TET2_CD_ was sufficiently active at 1 nM (Figure S3A–C). The *K*_M_^app^ (2OG) values of TETs measured using AlphaScreen were 2.4 ± 0.3 μM (TET1_CD_) and 11.1 ± 3.5 μM (TET2_CD_) under nonsaturating substrate conditions (10 nM ^5m^C-DNA **1**, Figure S4), which are similar to reported *K*_M_ (2OG) of 15.7 ± 2.8 μM for TET2_CDΔLCI_ using a mass spectrometry (MS) method.^42^

### Inhibitor Screening against TET Enzymes Using the AlphaScreen Assay

**Figure 2:**
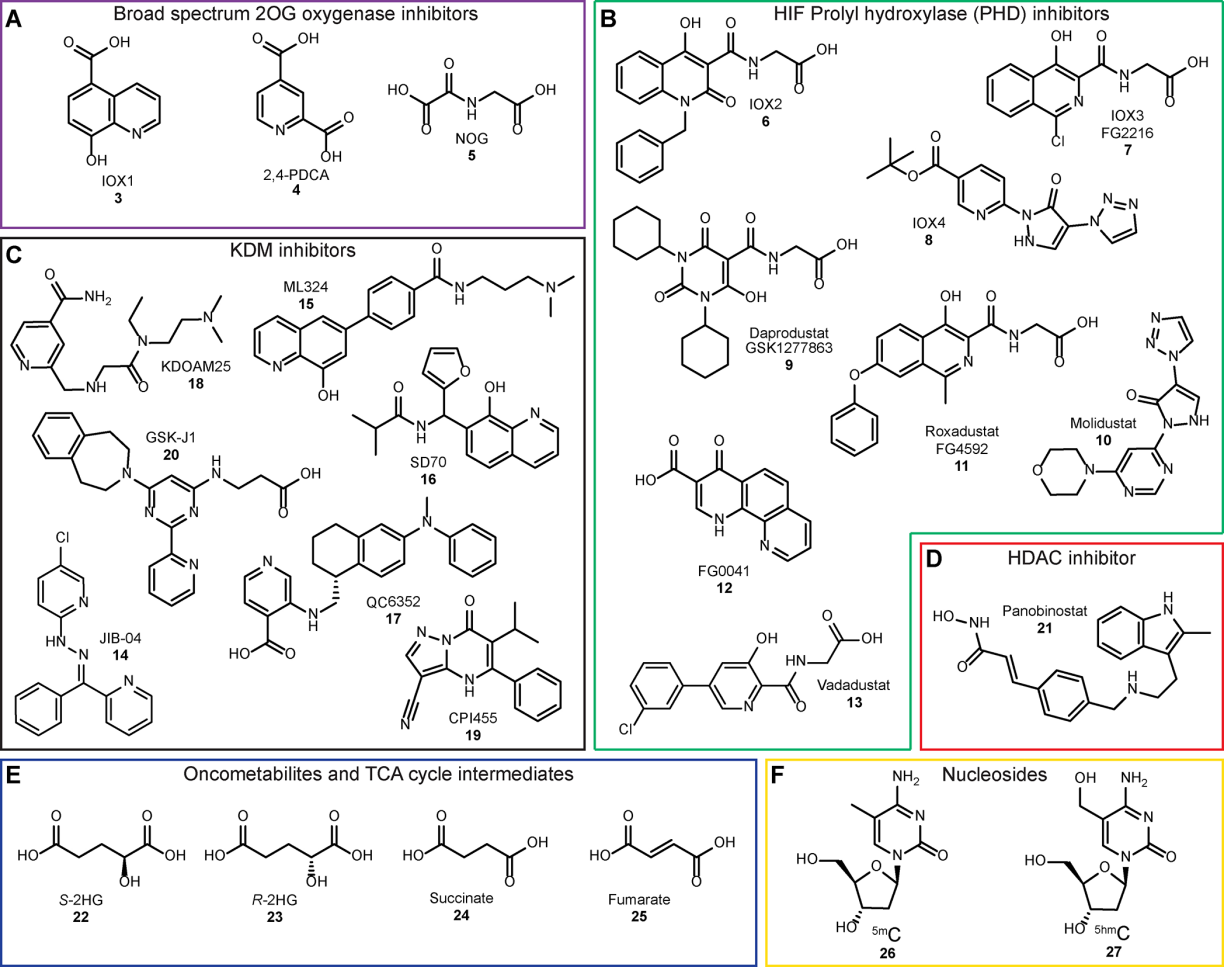
Small molecule inhibitors tested against TET1–3. The focused panel contains broad-spectrum 2OG oxygenase inhibitors (A, purple), HIF PHD inhibitors (B, green), KDM targeted inhibitors (C, black), HDAC inhibitor (D, red), TCA cycle intermediates and related compounds (E, blue), and relevant nucleosides (F, yellow).

**Figure 3:**
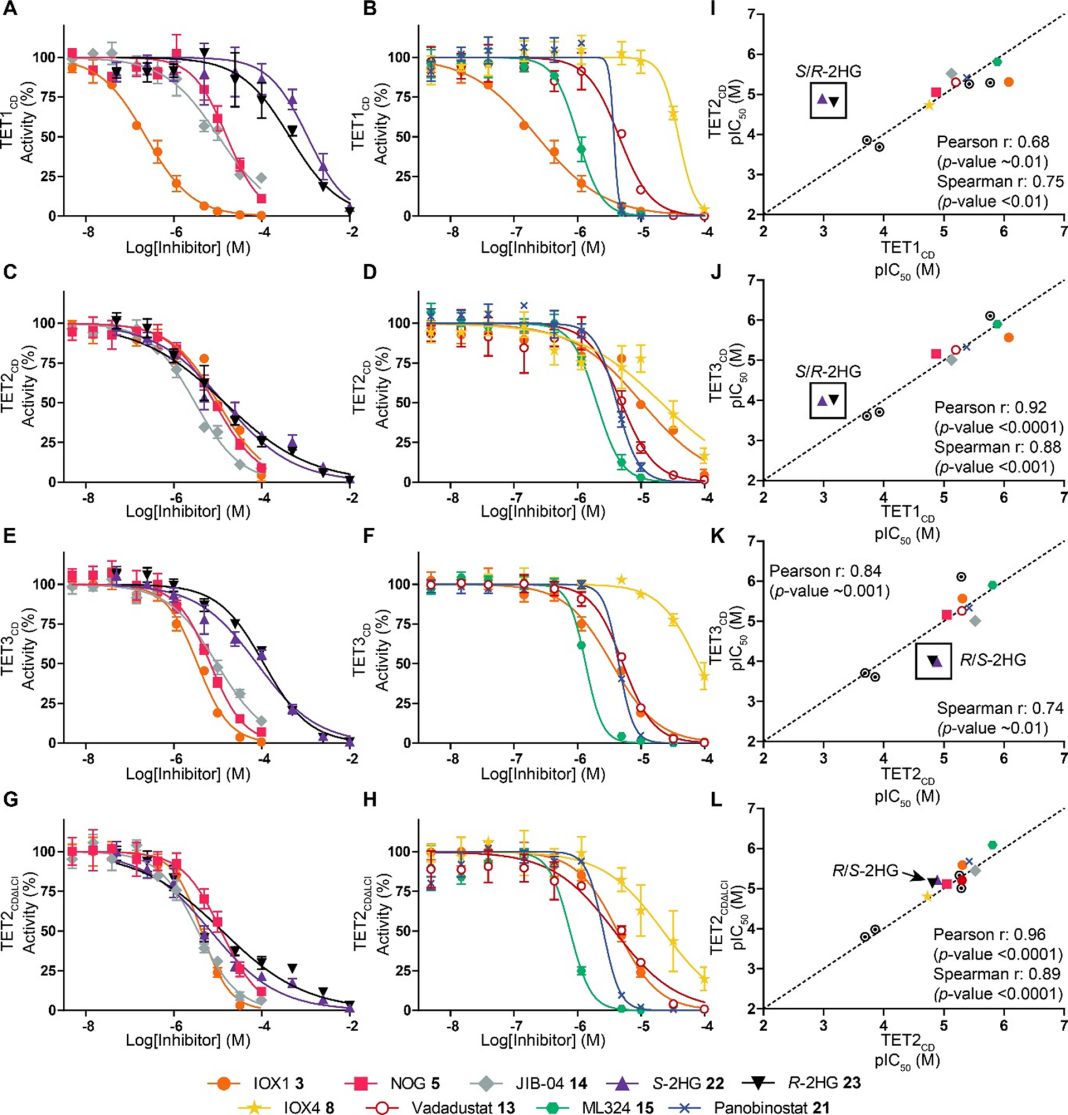
Representative AlphaScreen IC_50_ curves and correlation curves for inhibitors tested against TET1–3_CD_ and TET2_CDΔLCI_. Plots for TET1_CD_ (A,B), TET2_CD_ (C,D), TET3_CD_ (E,F), and TET2_CDΔLCI_ (G,H) of selected inhibitors are shown. IOX1 (3, orange), NOG (5, pink), JIB-04 (14, gray), *S*-2HG (22, purple), *R*-2HG (23, black), ML324 (15, green), Vadadustat (13, red), IOX4 (8, yellow) and Panobinostat (21, blue). I-L AlphaScreen pIC_50_ correlation plots for inhibitors for TET1–3_CD_, TET2_CDΔLCI_. Pearson correlation and Spearman coefficients were calculated for comparisons. Standard conditions: ^5m^C (1, 10 nM), ascorbate (100 μM), Fe(II) (10 μM), 2OG (10 μM), with TET1_CD_ (10 nM, 30 min incubation), TET2_CD_ (1 nM, 10 min incubation), TET3_CD_ (10 nM, 10 min incubation), or TET2_CDΔLCI_ (1 nM, 10 min incubation). *n* = 2–4, error given as ± StDev. IC_50_ values are displayed in Table 1; compound structures are given in Figure 2. Circled dots—other compounds from Table 1.

To investigate the selectivity of TET inhibition, a set of 2OG oxygenase–focused inhibitors, including broad-spectrum inhibitors,^44,45^ PHD-selective inhibitors,^46,47^ JmjC-KDM-selective inhibitors,^48^ (2*R*) and (2*S*)-hydroxyglutarate^33^ (*R/S*-2HG), TCA cycle metabolites and other epigenetic protein inhibitors,^49^ was tested using the AlphaScreen method^37^ (Figure 2). IOX1 **3**, a broad-spectrum 2OG oxygenase inhibitor and relatively potent inhibitor of TET1/2,^37^ was used as a positive control. As most reported 2OG oxygenase inhibitors are 2OG competitors and contain an Fe(II) chelating group,^20^ IC_50_ screens were carried out at approximate *K*_M_^app^ of 2OG (10 μM) (Figure S4) in the linear ranges using TET1 (10 nM), TET2 (1 nM), or TET3 (10 nM). Most tested compounds inhibited TET1–3 to varying degrees (Table 1 and Figure 3A–F).

**Table 1:** Results of Inhibitors Screened against the Human TETs^a^

compound		AlphaScreen plC_50_				SPE-MS pIC_50_	Cellular pEC_50_	Cellular pCC_50_
		TET1_CD_	TET2_CD_	TET3_CD_	TET2_CDΔLCI_	TET2_CDΔLCI_	TET1_CD_	TET1_CD_
IOX1	**3**	6.08 ± 0.64	5.31 ± 0.24	5.57 ± 0.19	5.59 ± 0.26	5.76 ± 0.05	4.99 ± 0.32 (12)	<3
2,4-PDCA	**4**	5.77 ± 0.79	5.29 ± 0.07	6.11 ± 0.10	5.01 ± 0.10	5.82 ± 0.09	<3^b^(4)	<3^b^
NOG	**5**	4.87 ± 0.21	5.05 ± 0.19	5.16 ± 0.01	5.12 ± 0.58	6.30 ± 0.10	3.96 ± 0.42^b^ (10)	<3^b^
IOX2	**6**	61% ± 16	32% ± 11	N.I.	33% ± 3	4.78 ± 0.02	≤3 (2)	<3
IOX3	**7**	43% ± 18	42% ± 7	47% ± 2	46% ± 21	5.29 ± 0.05	<3 (2)	<3
IOX4	**8**	4.76 ± 0.39	4.73 ± 0.03	64% ± 9	4.80 ± 0.18	4.52 ± 0.19	4.10 ± 0.32 (13)	3.01 ± 0.42
Daprodustat	**9**	63% ± 25	31% ± 6	36% ± 10	27% ± 12	4.38 ± 0.02	<3 (1)	N.D.
Molidustat	**10**	N.I.	N.I.	60% ± 3	N.I.	N.I.	∼4 (1)	<3.7
Roxadustat	**11**	25% ± 6	44% ± 13	30% ± 16	N.I.	N.D.	<3 (3)	N.D.
FG0041	**12**	5.42 ± 0.26	5.26 ± 0.53	N.D.	5.33 ± 0.01	5.25 ± 0.02	4.62 ± 0.29 (2)	<3.7
Vadadustat	**13**	5.20 ± 0.24	5.30 ± 0.01	5.26 ± 0.03	5.21 ± 0.02	5.69 ± 0.06	<3 (4)	N.D.
JIB-04	**14**	5.13 ± 0.20	5.52 ± 0.01	5.01 ± 0.12	5.45 ± 0.01	52% ± 2	6.36 ± 0.42 (4)	5.01 ± 0.66
ML324	**15**	5.89 ± 0.21	5.81 ± 0.10	5.90 ± 0.04	6.09 ± 0.04	4.74 ± 0.18	4.28 ± 0.42 (5)	4.14 ± 0.17
SD70	**16**	35% ± 29	47% ± 4	55% ± 7	54% ± 1	58% ± 14	N.D.	N.D.
QC6352	**17**	28% ± 1	30% ± 8	29% ± 2	62% ± 2	34% ± 6	N.D.	N.D.
KDOAM25	**18**	N.I.	N.I.	N.I.	30% ± 6	N.I.	<3 (2)	<3.7
CPI455	**19**	N.I.	N.I.	N.I.	N.I.	N.I.	N.D.	N.D.
GSK-J1	**20**	26% ± 9	22% ± 2	N.D.	N.I.	N.D.	4.80 ± 0.56^b^ (4)	3.94 ± 0.26^b^
Panobinostat	**21**	5.38 ± 0.30	5.42 ± 0.31	5.33 ± 0.03	5.68 ± 0.01	4.62 ± 0.11	<3^c^(4)	∼8
*S*-2HG	**22**	2.98 ± 0.05	4.89 ± 0.06	3.98 ± 0.17	5.21 ± 0.07	5.87 ± 0.52	N.I.^b^ (3 mM) (3)	<2.7^b^
*R*-2HG	**23**	3.17 ± 0.24	4.81 ± 0.03	4.02 ± 0.11	5.14 ± 0.35	5.93 ± 0.47	2.83 ± 0.09^b^ (3)	<2.7^b^
succinate	**24**	3.72 ± 0.27	3.86 ± 0.18	3.61 ± 0.05	3.98 ± 0.31	4.83 ± 0.35	2.46 ± 0.10^b^ (3)	2.27 ± 0.16^b^
fumarate	**25**	3.93 ± 0.14	3.69 ± 0.08	3.71 ± 0.03	3.80 ± 0.35	5.03 ± 0.23	3.50 ± 0.15^b^ (3)	3.57 ± 0.15^b^
^5m^C	**26**	N.D.	N.D.	N.D.	N.D.	N.I.	N.D.	N.D.
^5hm^C	**27**	N.D.	N.D.	N.D.	N.D.	N.I.	N.D.	N.D.
dimethyl-2OG	**33**	N.D.	N.D.	N.D.	N.D.	N.D.	2.17^b^	<2^b^

a pIC_50_, pEC_50_, and pCC_50_ values of small molecule inhibitors tested against human TET1-3 using AlphaScreen, SPE-MS assays or cellular IF assays. Values of enzyme assays are indicated as pIC_50_, with incomplete dose–response curves noted as % inhibition at 100 μM. Inhibition of cellular TET1 activity was measured as pEC_50_ values with independent biological replicates in parentheses (*n*). pCC_50_ values are based on the nuclear count measured by DAPI staining. N.I. indicates no inhibition at 100 μM (<20%), N.D.: not determined. Values are mean ± StDev, *n*= 2–5. See Figure 2 for compound structures and Figure S10 for prodrug structures. AlphaScreen IC_50_ not determined for nucleosides ^5m^C and ^5hm^C due to antibody assay interference.

b Ester prodrug used.

c Apparent increase of ^5hm^C levels.

The broad-spectrum inhibitors (purple box, Figure 2), such as IOX1 **3**, 2,4-pyridine-dicarboxylic acid (2,4-PDCA, **4**), and *N-*oxalylglycine (NOG, **5**), were approximately equipotent inhibitors of TET1–3, with IC_50_ values in the low micromolar range (pIC_50_ = ∼5–6). While IOX1 **3** inhibition was similar to that reported,^37^ NOG **5** inhibited TET1_CD_, TET2_CD_, and TET3_CD_ with IC_50_ values of 13, 9, and 7 μM, respectively, an order of magnitude more potent than reported for TET2_CDΔLCI_ using an MS assay (IC_50_ = 149 ± 8 μM; enzyme concentration: 5 μM).^42^ The PHDs are validated therapeutic targets, with several inhibitors for them being approved or in development for treatment of anemia associated with chronic kidney disease^47,49^ (Figure 2). Weak or no inhibition (IC_50_ ≥ 100 μM, pIC_50_ ≤ 4) of TETs was observed for Daprodustat (GSK1277863, **9**), Roxadustat (FG4592, **11**), and their analogues IOX2 **6** and IOX3 (FG2216, **7**).^47^ Molidustat (BAY85-3934, **10**) also showed little inhibition, but its analogue IOX4 **8** was a moderately potent inhibitor of TET1_CD_ and TET2_CD_ with IC_50_ values of 17 and 19 μM, respectively. FG0041 **12** and Vadadustat (AKB-6548, **13**) were the most potent TET inhibitors of the PHD inhibitors tested, with low single-digit micromolar IC_50_ values (Table 1).

JIB-04 **14** and ML324 **15** (Figure 2), which were initially reported as selective JmjC-KDM inhibitors^50^ but were subsequently shown to have wider inhibitory profiles,^51,52^ and showed here relatively potent TET1–3 inhibition (Table 1). JmjC-KDM inhibitors with higher KDM selectivity, i.e., GSK-J1 **20**,^53,54^ QC6352 **17**,^55,56^ KDOAM25 **18**,^57^ and CPI455 **19**,^58^ were either very weak inhibitors (IC_50_ > 100 μM) or did not inhibit TET1–3. SD70 **16**, a reported inhibitor of ligand and genotoxic stress-induce translocations in prostate cancer and which was later identified to inhibit KDM4C,^59^ was a weak TET inhibitor (35–55% at 100 μM SD70 **16**). The broad-spectrum histone deacetylase (HDAC) inhibitor Panobinostat **21** (red box, Figure 2), an approved drug for multiple myeloma by the U.S. Food and Drug Administration and European Medicines Agency, inhibited TET1–3 (IC_50_ = 3.8–4.7 μM) albeit with a steep hill slope for TET1_CD_ (>4); note that hydroxamic acids chelate Fe(II) and are reported to inhibit some 2OG oxygenases including JmjC-KDMs.^44,60,61^

The oncometabolite *R-*2HG (D-2HG, **23**) is reported to inhibit the TETs.^34^ Despite the weak observed inhibition using recombinant murine and human TETs (murine Tet1/2 IC_50_ = 4–5 mM^34,43^ and human TET2_CDΔLCI_ IC_50_ = 5.3 mM,^42^Table S2), it was proposed that elevated levels of *R-*2HG **23** in IDH mutant cells may be sufficient to impact on global ^5hm^C levels via TET inhibition.^34^ In the AlphaScreen assay, *S-* and *R-*2HG **22**, **23** were found to be very weak inhibitors of TET1_CD_ (IC_50_ = ∼0.8 mM, for both enantiomers, respectively). Unexpectedly, both *S-* and *R-*2HG **22**, **23** were moderate and weak inhibitors of TET2_CD_ (IC_50_ = 13–15 μM, respectively) and TET3_CD_ (IC_50_ = ∼100 μM for both 2HG enantiomers), respectively (Table 1). Thus, while the 2HG potencies differed for different TET subfamily members, *S*- and *R*-2HG **22**, **23** manifested equal potency versus the individual TET enzymes. The TCA cycle intermediates succinate **24** and fumarate **25** were weak inhibitors of TET1–3 (IC_50_ = 118–245 μM), in accord with results for murine Tet1/2.^43^

### LCI in the Catalytic Domain of TET2_CD_ Has Little Impact on the Inhibition Profile

The above-mentioned results reveal TET1–3 shares relatively similar inhibition profiles, with the exceptions of *S*- and *R*-2HG **22**, **23** (Figure 3I–K). The differential 2HG potencies are particularly evident when comparing inhibition of TET1_CD_ and TET2_CD_ by both *S*- and *R*-2HG **22**, **23**; both enantiomers are >43-fold more potent for TET2_CD_ than TET1_CD_. The apparent discrepancy in our IC_50_ values and those reported for TET2_CDΔLCI_,^42^ a TET2 construct without the LCI (Figure 1C), prompted us to investigate the effect of the LCI on TET catalysis and inhibition. Recombinant TET2_CDΔLCI_ (D1129–G1936 with S1481–N1843 replaced by a 3 × GGGGS linker) was produced in *E. coli* and purified as reported,^19^ yielding relatively high levels of purified protein (∼1.6 mg L^–1^, > 85% purity by SDS-PAGE) (Figures 1C and S1). TET2_CDΔLCI_ showed similar activity to TET2_CD_ (specific activity = 0.14 μM min^–1^ μM^–1^, *K*_M_^app^ (2OG) = 4.9 ± 0.3 μM) (Figures S3D and S4) and its inhibition profile correlated with that of TET2_CD_ (Pearson rank = 0.96, Spearman rank = 0.89) (Table 1, Figures 2 and 3G,H,L). *S*-2HG **22** and *R*-2HG **23** inhibit TET2_CDΔLCI_ with single-digit micromolar potency (*S*-2HG **22** IC_50_ = 6.0 μM and *R-*2HG **23** IC_50_ = 7.2 μM). Further kinetic studies with both TET2 enzymes using AlphaScreen assays revealed IOX1 **3** to be a 2OG competitive inhibitor (Table S1 and Figure S5), consistent with studies on other 2OG oxygenases.^45^*S*-2HG **22** and *R*-2HG **23** showed similarly potent 2OG competitive inhibition with TET2_CD_ (*S*-2HG **22***K*_*i*_ = 19 μM, *R*-2HG **23***K*_*i*_ = 23 μM) and TET2_CDΔLCI_ (*S*-2HG **22***K*_*i*_ = 6 μM, *R*-2HG **23***K*_*i*_ = 12 μM) (Table S1 and Figure S5B,C). These observations suggest that the presence of the LCI has little impact on the inhibition profile, at least under our assay conditions. TET2_CDΔLCI_ is thus a good model enzyme for kinetic studies for TET2 due to its ease of production and was used in subsequent MS assays, which have higher enzyme requirements than the AlphaScreen assay. For TET1_CD_, the *K*_i_ values of *S*-2HG **22** and *R-*2HG **23** were 269 and 246 μM, respectively (Table S1), which were notably less potent (>10-fold) than against TET2_CD/CDΔLCI_.

### Mass Spectrometry-Based Assays for the Detection of TET Activity on Double-Stranded DNA

**Figure 4:**
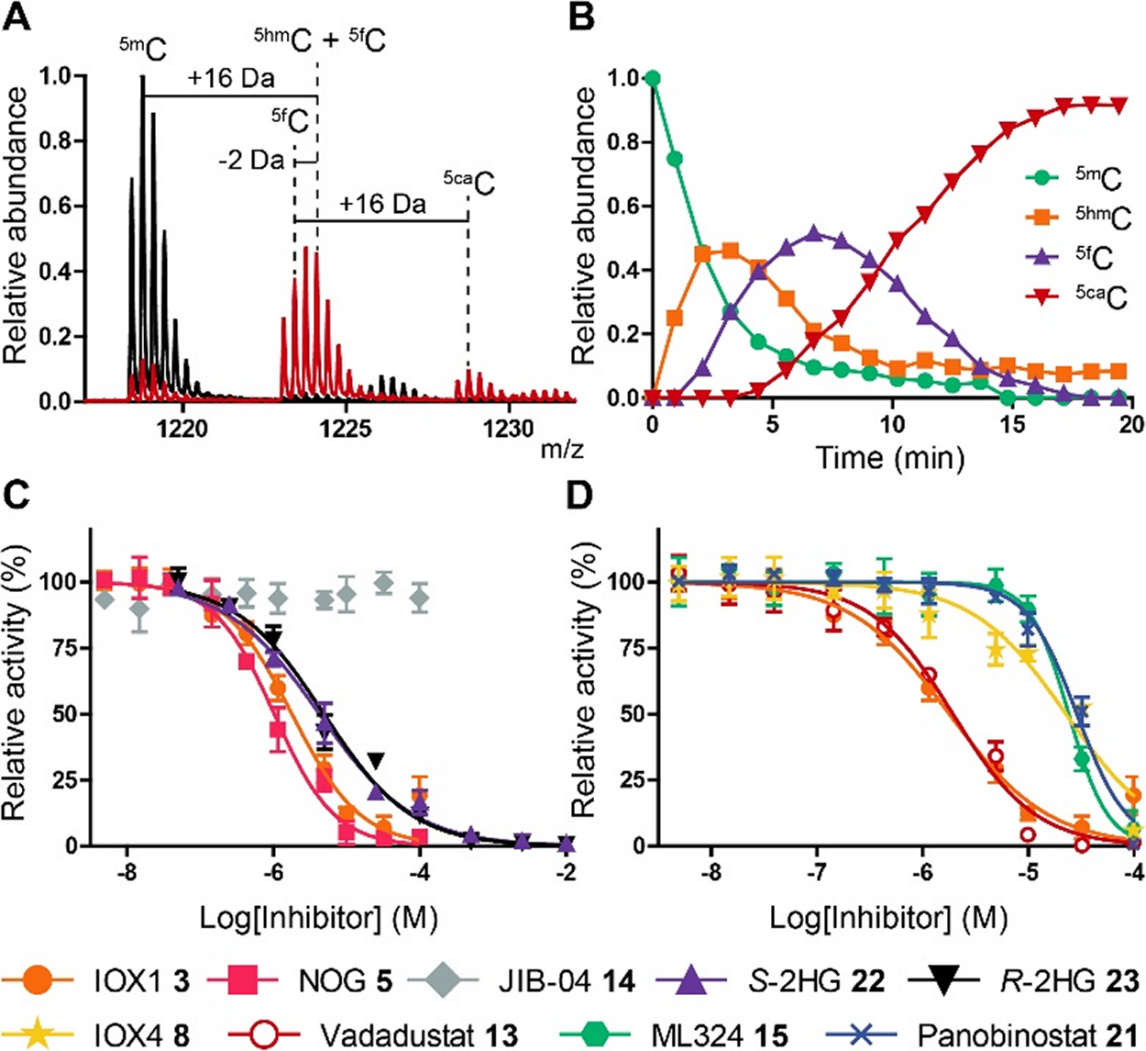
Measurement and quantitation of TET2_CDΔLCI_ catalyzed oxidation of ^5m^C to ^5hm^C, ^5f^C, and ^5ca^C using the SPE-MS assay. (A) Representative SPE-MS spectra for TET2_CDΔLCI_ (0.8 μM) catalysis in the presence of ^5m^C **28** (1.0 μM) using the [M–3H]^3–^ charge state. Overlay of spectra from 0 min (black) and 5.57 min (red) time points showing sequential oxidation of ^5m^C to ^5hm^C (+16 Da relative to ^5m^C), ^5f^C (+14 Da relative to ^5m^C), and ^5ca^C (+30 Da relative to ^5m^C). (B) Corresponding time course displaying the relative abundance of ^5m^C **28** (green), ^5hm^C (orange), ^5f^C (purple), and ^5ca^C (red). (C,D) Representative IC_50_ curves of IOX1 (**3**, orange), NOG (**5**, pink), JIB-04 (**14**, gray), *S*-2HG (**22**, purple), *R*-2HG (**23**, black), IOX4 (**8** yellow), Vadadustat (**13**, red), ML324 (**15**, green), and Panobinostat (**21**, blue) tested with TET2_CDΔLCI_ (0.4 μM) and ^5m^C-DNA **28** (2.0 μM). Assays were quenched after 10 min (∼20–30% ^5hm^C product formation at a linear range (*R*^2^: 0.99, Figure S6C)) to minimize formation of subsequent oxidative products ^5f^C and ^5ca^C. Standard conditions: ^5m^C-DNA (**28**, 2.0 μM) and TET2_CDΔLCI_ (0.4 μM), ascorbate (200 μM), Fe(II) (50 μM), 2OG (10 μM). Data are plotted as mean (*n* = 2–4) and error given as ± StDev.

While the AlphaScreen method provides a high-throughput, sensitive assay with low reagent requirements, the indirect immunobead-based detection can lead to assay interference and false positives. ssDNA, a possibly less efficient TET substrate than dsDNA,^27^ was used in AlphaScreen assays because it yielded significantly higher signals than dsDNA, possibly due to increased accessibility of the ^5hm^C-antibody to ^5hm^C-DNA. Thus, an MS assay was developed to directly measure substrate depletion/product formation using dsDNA. Reported MS methods for TET assays, including for kinetic studies, are typically based on LC-MS/MS which employ oligonucleotide hydrolysis to nucleosides for analysis.^38^ However, the LC-MS methods require higher amounts of substrate/enzymes and can require downstream processing steps. To improve sample throughput and assay resolution, we developed a method consisting of solid-phase extraction coupled to MS assay (SPE-MS), as has been applied to other 2OG oxygenases.^62^ The SPE-MS method enables fast (<12 s) sample processing, near real-time monitoring of TET-mediated ^5m^C oxidation and is based on a reported method.^63^ The SPE-MS assay was optimized with the palindromic 12-base pair ^5m^C-DNA (ACCAC^5m^CGGTGGT, **28**) with TET2_CDΔLCI_, which enabled good sensitivity and robust detection of activity. A time course for TET2_CDΔLCI_ in the presence of ^5m^C-DNA **28** resulted in the resolution of substrate and products, including all three oxidized states of cytosine (Figure 4A,B). To measure ^5hm^C and ^5f^C, which have a 2 Da mass difference and overlapping isotopic patterns, peak area extraction was carried out to estimate their quantities relative to ^5m^C and ^5ca^C (Figure 4A). ^5m^C was observed to be sequentially oxidized in a time-dependent manner to give ^5hm^C, then ^5f^C, and finally ^5ca^C (Figure 4B). After 20 min ^5ca^C-DNA was the major product observed (∼90%), followed by ^5hm^C-DNA (∼10%), under our assay conditions. No evidence for oxidation of a potential thymidine substrate to 5-hydroxymethyluracil (^5hm^U), a reported TET product,^24^ was detected. Kinetic analysis of TET2_CDΔLCI_ with saturating ^5m^C-DNA **28** (i.e., above the *K*_M_**28** of 1 μM as determined by matrix-assisted laser desorption/ionization time-of-flight (MALDI-TOF) MS,^42^Figure S6A) yielded *K*_M_^app^ (2OG) of 3.0 ± 0.6 μM (Figure S6B), similar to that observed with the AlphaScreen assay (Figure S4).

### Inhibitor Screening against TET2_CDΔLCI_ Proteins Using SPE-MS

The TET2_CDΔLCI_ inhibition profile using SPE-MS (Table 1, Figures 4C,D and 6C) was in general agreement with the AlphaScreen results (Table 1 and Figure S6D). IOX1 **3** gave a similar level of inhibition (IC_50_ = 1.7 μM) relative to AlphaScreen. Interestingly, in the SPE-MS assay NOG **5**, a near 2OG isostere, showed increased potency (ΔpIC_50_ = +1.18), while JIB-04 **14** (ΔpIC_50_^est^ = −1.43), ML324 **15** (ΔpIC_50_ = −1.35) and Panobinostat **21** (ΔpIC_50_ = −1.06) gave reduced potency. The difference in potencies observed between the two assays (Pearson correlation coefficient r: 0.29; Spearman correlation coefficient *r*: 0.15, Figure S6D) could, in part, reflect the increased Fe(II) concentration in the SPE-MS compared to the AlphaScreen assay (50 and 10 μM, respectively).^50^ SPE-MS enabled the analysis of ^5m^C **26** and ^5hm^C **27** nucleosides as potential inhibitors, but no inhibition was observed at 100 μM, indicating that the oligomeric DNA context is important in TET binding. Importantly, the SPE-MS results confirmed both *S*- and *R*-2HG **22**, **23** are relatively potent TET2_CDΔLCI_ inhibitors.

### Cellular Inhibition of TETs by Small Molecules

**Figure 5:**
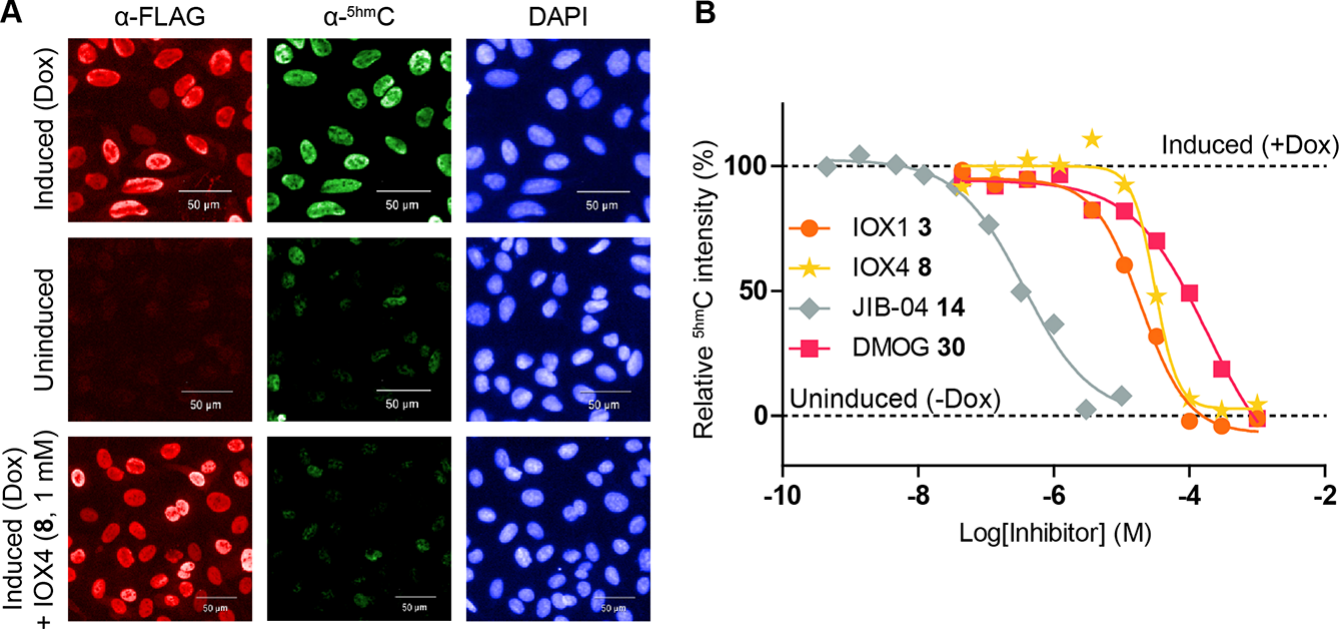
Small molecule inhibitors of TETs can reduce global ^5hm^C levels in cells. (A) Selected images of IF staining of Dox-inducible U2OS cells stably transfected with FLAG-tagged TET1_CD_. An increase in FLAG (red) and ^5hm^C (green) staining, corresponding to overexpression of catalytically active TET1_CD_, is observed only after Dox (1 mg mL^–1^)-mediated induction. DAPI nuclear staining is in blue. Reduction in the ^5hm^C level is observed while FLAG staining is maintained when cells are treated with TET inhibitors (e.g., IOX4 **8**). This trend corresponds to observations with cells overexpressing a catalytically inactive TET1_CD_ mutant (Figures S7–S9). (B) Representative EC_50_ curves for IOX1 **3**, IOX4 **8**, JIB-04 **14**, and DMOG **30** for Dox-induced U2OS cells overexpressing TET1_CD_. All tested compounds reduce ^5hm^C levels in a dose-dependent manner. The ^5hm^C levels of Dox-induced and -uninduced control cells (1% DMSO) are indicated. Data are plotted as mean and error given as ± s.e.m (*n* > 3000 cells). See Figures S9 and S11 for dosing data on TET1_CD_ MUT.

To evaluate the effect of TET inhibition in cells, we developed an immunofluorescence (IF) assay to measure changes in genomic ^5hm^C levels as a function of TET1 activity. Stable U2OS cells with doxycycline (Dox)-inducible FLAG-tag wild-type (WT) TET1_CD_ and a catalytically inactive variant (MUT, H1672A/D1674A) TET1_CD_ were generated (Figure S7). FLAG staining was observed only in Dox-induced cells and colocalized with DAPI in the nucleus, indicating *TET1*_CD_ was expressed. Dox-dependent *TET1*_CD_ expression was shown by Western blots (Figure S7A–C). Next, U2OS cells were induced with Dox for 24 h, fixed, and stained with either ^5hm^C- and FLAG-antibodies, fluorescence-conjugated secondary antibodies or DAPI. The cells were visualized and quantitated using high-content imaging (Figure 5A). Substantial staining for ^5hm^C was detected in Dox-induced WT *TET1*_CD_ overexpressing cells, while minimal background ^5hm^C levels were detected in uninduced and MUT *TET1*_CD_ overexpressing cells (Figures 5A, S8A,B and S9A), implying that the WT TET1_CD_ efficiently catalyzes oxidation of ^5m^C to ^5hm^C in cells.

Next, we assessed inhibitors (Figure 2) or prodrug equivalents (Figure S10) in U2OS cells. Compounds were dosed for 24 h with simultaneous induction of TET1_CD_ expression, and IF analysis of FLAG and ^5hm^C levels was performed (Figure 5 and Table 1). Uninduced TET1_CD_WT and induced TET1_CD_MUT were used as negative controls (Figures S9 and S11). Of the broad-spectrum inhibitors, IOX1 **3** was relatively potent against TET1_CD_ (EC_50_ = 10 μM), while dimethyloxalylglycine (DMOG, **30**, prodrug of **5**) (Figure 5B) was a weaker inhibitor (EC_50_ = 110 μM) and dimethyl 2,4-PDCA (**31**, prodrug of **4**) showed no inhibition at 1 mM. In agreement with the isolated enzyme assays, most of the PHD-selective inhibitors (IOX2 **6**, IOX3 **7**, Daprodustat **9**) were not TET1_CD_ inhibitors in cells, except for Molidustat **10** where weak inhibition (EC_50_ = ∼100 μM) was observed. IOX4 **8**, an analogue of Molidustat **10** showed similar inhibitory activity in cells (EC_50_ = 79 μM). Interestingly, Vadadustat (AKB-6548, **13**) inhibited TETs at single-digit micromolar potency in enzyme assays but exhibited no measurable inhibition in the cellular assay. FG0041 (**12**) showed both activity in both isolated enzyme and cellular assays (EC_50_ = 24 μM). Note there is no information on the relative efficiencies of cellular uptake/efflux/metabolism of these inhibitors in U2OS cells so the differences between TET inhibition on isolated enzymes and of TET1_CD_ in cells may, in part, reflect these factors.

**Figure 6:**
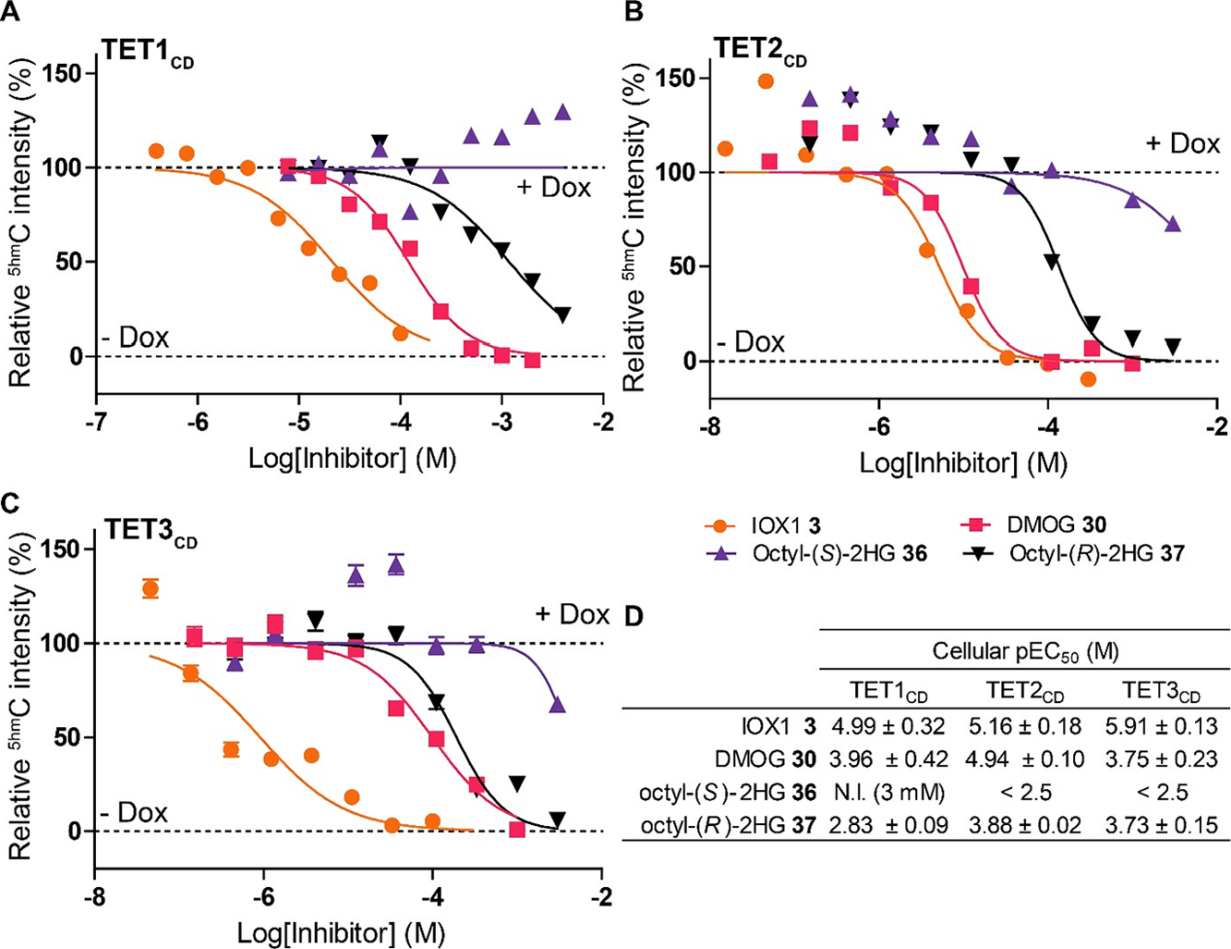
Evidence that the TET isozymes have different sensitivities to 2-hydroxyglurate enantiomers (2HGs) in cells. Representative dose–response curves for IF cell assays for U2OS cells stably expressing TET1_CD_ (A), TET2_CD_ (B), and TET3_CD_ (C) dosed with inhibitors for 24 h. Data are normalized to ^5hm^C levels of doxycyclin-induced TET-expressing cells (+Dox) and uninduced (-Dox) cells treated with 1% DMSO. Data are plotted as mean, and the error is given as ± s.e.m (*n* > 3000 cells). (D) Tabulated cellular pEC_50_ values of inhibitors in IF assays for TET1_CD_, TET2_CD_, and TET3_CD_. Data are shown as mean with error given as ± StDev. of independent biological replicates with the number of replicates in brackets. N.I.: no inhibition at 3 mM. See Figure S13 for associated FLAG staining, cell counts, and TET1_CD_ MUT data.

Out of the KDM inhibitors tested, JIB-04 **14** and ML324 **15** were the only compounds that inhibited TET1_CD_ with both the isolated enzyme and cellular assays. JIB-04 **14** was the most potent TET inhibitor in cells, with an EC_50_ of 0.44 μM, which is substantially more potent than observations with the AlphaScreen enzyme assay (IC_50_ = 7.4 μM). Although no changes in ^5hm^C levels were observed with TET1_CD_MUT, JIB-04 **14** induced a concentration-dependent decrease in cell numbers (CC_50_ = ∼10 μM); thus, nonspecific mechanisms causing toxicity cannot be ruled out. Similar trends have been observed for JIB-04 **14** treatment of JmjC KDM overexpressing HeLa cells, suggesting a likely promiscuous mechanism for JIB-04 **14**.^52^ GSK-J4 (**32**, prodrug of **20**) showed apparent cell activity at EC_50_ = 16 μM, while exhibiting no inhibition against recombinant TET1_CD_. Interestingly, increased levels of ^5hm^C were observed when cells were dosed with the HDAC inhibitor Panobinostat **21**, despite being a moderate inhibitor of isolated TET1_CD_ (IC_50_ = 4.2 μM) (Figure S12). Increased staining of ^5hm^C was observed below 11 μM (up to 1.5-fold relative to the DMSO control) with a concomitant increase in FLAG staining/TET1 expression, followed by a rapid reduction in ^5hm^C levels at >33 μM, correlating with a dose-dependent reduction in cell counts (Figures S11 and S12). Prodrugs of TCA cycle intermediates, dimethyl esters of 2OG **33**, succinic acid **34**, and fumaric acid **35**, were also tested. Dimethyl 2OG **33** and dimethyl succinate **34** were very weak potential TET1_CD_ inhibitors in cells (EC_50_ = ∼6.7 and ∼3.5 mM, respectively). Dimethyl fumarate **35** inhibited with an EC_50_ of 316 μM (Table 1), but also induced cell death at the same concentration range (CC_50_ = 270 μM). While both *S*- and *R*-2HGs have similar potencies against isolated recombinant TET1, their prodrugs showed different activities in cells. Octyl-ester *R-*2HG **37** had very weak inhibitory activity on TET1 in cells (∼50% inhibition at 1.5 mM), and no inhibition was observed for octyl-ester of *S-*2HG **36** at 3 mM (Table 1 and Figure 6A).

To investigate the differential sensitivities of TET subfamily members to 2HG observed in isolated enzymes, we generated stable U2OS cell lines with Dox-inducible expression of FLAG-tag WT TET2_CD_ and TET3_CD_. After dox-dependent TET gene expression was confirmed by Western blot and IF staining (Figures S7D,E and S8C,D), TET2_CD_ and TET3_CD_ cells were treated with IOX1 **3**, DMOG **30** or octyl-ester 2HGs **36**, **37** and analyzed by IF. IOX1 **3** was a potent inhibitor for both TET2_CD_ and TET3_CD_ (EC_50_ = 6.9 μM and 1.2 μM, respectively). DMOG **30** manifested EC_50_ values of 11 and 177 μM, for TET2_CD_ and TET3_CD_, respectively, a similar range of inhibition observed for TET1_CD_. The octyl-ester of *R-*2HG **37** showed modest, but clear, inhibition of both TET2_CD_ (EC_50_ = 131 μM) and TET3_CD_ (EC_50_ = 186 μM) in cells (Figure 6B–D). Similar to the results for TET1, the octyl-ester *S-*2HG **36** poorly inhibited TET2_CD_ or TET3_CD_ in cells (up to 3 mM octyl-ester *S-*2HG **36** tested). Thus, the trends of recombinant TET inhibition reflect the cellular inhibition for the *R-*2HG **37,** that is, *R*-2HG is a more potent inhibitor of TET2 and TET3, than TET1.

## Discussion and Conclusions

We have developed robust, sensitive, and quantitative assay platforms for the discovery of inhibitors for TET1–3. Using complementary AlphaScreen and MS-based assays, we found the 2OG *K*_M_ values for TET1/2 to be in the micromolar range, in agreement with reported studies^42^ and similar to reported values for many, but not all, 2OG oxygenases^45,64^ (Table S2). These assays were used to screen a focused set of 2OG oxygenase inhibitors against isolated catalytic domains of TET1–3, using 2OG at its *K*_M_ value and using low enzyme concentrations (nM). Consistent with reports, broad-spectrum 2OG oxygenase inhibitors, including IOX1 **3**, 2,4-PDCA **4**, and NOG **5**, were inhibitors of TET1–3. Our IC_50_ values are generally lower than those reported,^42^ reflecting the increased sensitivity of our assays that allow lower enzyme concentrations to be used (Table 1).

Under physiological conditions, intracellular 2OG concentrations (0.6–2.7 mM^65,66^) are estimated to be significantly above the calculated *K*_M_ for 2OG of the TETs (2–11 μM, Figure S4). Treatment of TET1 overexpressing cells with a prodrug of 2OG (dimethyl 2OG, **33**) showed no enhancement in ^5hm^C levels (Table 1), an observation which implies that the TET activity is not limited by 2OG availability under our cellular assay conditions. Most of the inhibitors identified in the screens with isolated TET1–3 CDs (or corresponding ester-prodrugs) showed cellular TET activity, with a dose-dependent reduction in the TET-catalyzed oxidation of ^5m^C to ^5hm^C, albeit in general with higher EC_50_ than IC_50_ values. For some compounds, cellular activity was not observed despite inhibition being observed with isolated TET(s), a discrepancy which might reflect low cell permeability, compound degradation, or rates of prodrug ester hydrolysis. Of particular interest, the PHD inhibitor Vadadustat **13**,^67^ developed for the treatment of anemia secondary to chronic kidney disease, was found to be among the most potent inhibitors of the TETs (IC_50_ = ∼5 μM) in enzyme assays, although activity was not observed in cell assays. However, it is possible that low levels of TET inhibition may contribute to the off-target effects associated with Vadadustat **13**, although it should be noted that the cellular relevance of our work is unclear at this stage.

Interestingly, the KDM inhibitors JIB-04 **14** and GSK-J1/4 **20**/**32** were the most potent cellular TET1 inhibitors identified, with submicromolar efficacy, but were less potent in the isolated TET assays. This difference may reflect effects resulting from dual KDM and TET inhibition; however, the lack of selectivity of JIB-04 **14** in cells may reflect inhibition of multiple 2OG oxygenases or effects on other relevant enzymes or variables such as iron or oxygen availability.^68^ In contrast to its inhibitory effect against the TETs, the HDAC inhibitor Panobinostat **21** appeared to increase ^5hm^C levels in cells in a concentration and TET1 activity-dependent manner. HDAC inhibition is shown to result in elevated levels of histone acetyl-lysine,^69^ an epigenetic mark associated with euchromatin. HDAC inhibition may improve the accessibility of DNA to TET1 as well as increase TET1 expression.

Most of the identified inhibitors were active versus all of TET1–3, likely in part, reflecting their 2OG competitive nature and conservation of the TET active sites. Both in isolated form and in cells, TET1–3 were inhibited by IOX1 **3** and NOG **5**, or its cell-penetrating derivative DMOG (Table 1 and Figure 6). DMOG is widely used in cellular studies and in animal models as inhibitors of 2OG oxygenases and as a hypoxia mimic. As suggested,^70^ some of the effects of DMOG may reflect inhibition of 2OG oxygenases other than the PHDs, including TET inhibition. The TCA cycle metabolites succinate **24** and fumarate **25** have also been shown to (weakly) inhibit several 2OG oxygenases, and were similarly weak (sub-mM) inhibitors of the TETs in our assays, in agreement with IC_50_ values reported for mouse Tet1/2.^43^ Exogenous treatment of succinate **24** and fumarate **25** prodrugs led to dose-dependent reductions in cellular TET1 activity and global reduction in ^5hm^C levels, albeit with concomitant cytotoxicity, so it is uncertain whether the effects are directly due to TET inhibition. Loss-of-function mutations of succinate dehydrogenase (SDH) and fumarate hydratase (FH) are found in multiple cancers^71,72^ and result in accumulation of high concentrations of succinate and fumarate, respectively, which are proposed to promote tumorigenesis; such elevated metabolite levels have been proposed to lead to reduced global ^5hm^C levels through TET inhibition.^43,73,74^

Importantly, our results provide unique and clear evidence for differential sensitivity to the oncometabolite *R*-2HG **23** for TET1–3; in isolated enzymes, TET2 is most sensitive (*K*_i_ = 12–23 μM; enzyme concentration: 1 nM), while TET3 was weakly inhibited (IC_50_ = ∼100 μM; enzyme concentration: 10 nM), and TET1 was poorly inhibited at sub-mM to mM concentrations (Figure 3 and Table 1). The observations imply stronger inhibition of TET2 by *R-* and *S-*2HG (*K*_*i*_ = 6–23 μM) than previously reported using the same protein construct (TET2_CDΔLCI_*R*-2HG and *S*-2HG IC_50_ = 5.3 mM and 12.4 mM, *K*_*i*_^calc^ = 0.9 mM and 2.1 mM, respectively) or related murine isoforms (*K*_*i*_^calc^ = 0.2–1.3 mM, Table S2). These differences may, at least in part, be due to the sensitivity of our assays. TET2 inhibition of *R*-2HG (*K*_*i*_ = 12–23 μM) is among the more potent values for inhibition of 2OG oxygenases by *R*-2HG, with KDM4A (*K*_*i*_ = 13 μM^75^) and KDM2A (IC_50_ = 106 μM^75^ and *K*_*i*_^calc^ = 74 μM) also manifesting potent inhibition. In contrast, other tested 2OG oxygenases such as PHD2 (*K*_i_ = 625 μM^76^), ABH2 (IC_50_ = 424 μM^75^ and *K*_*i*_^calc^ = 212 μM), or KDM5B (IC_50_ = 203 μM^77^ and *K*_*i*_ = 10 mM^34^) are weakly inhibited (Table S2). It should be noted that the precise assay conditions can influence potency of 2OG oxygenase inhibition.^78^ Nonetheless, in cellular assays, a prodrug form of *R*-2HG **37** showed similar trends in relative inhibition of the TET1–3 to that observed with isolated TET1–3 enzymes, with TET2 being the most sensitive isozyme (EC_50_ of TET2 (132 μM) < TET3 (186 μM) < TET1 (∼1.5 mM)). Little or no inhibition in cells was observed for the prodrug form of *S*-2HG **36** at 3 mM. The inactivity of *S*-2HG **36** may, in part, be due to poor prodrug hydrolysis and/or cellular uptake, or, less likely, differences in the intracellular dehydrogenases acting on the 2HG clearance^79^ (e.g., D2HGDH^80^ vs L2HGDH^81^). Inhibition of TETs by *R*-2HG is shown to result in aberrant ^5m^C/^5ox^C patterns in AML, and TET2 may be particularly affected due to its abundance and sensitivity. Elevated cellular *R-*2HG (and *S*-HG) levels are also found in 2-hydroxyglutaric aciduria, a rare neurometabolic disorder resulting from gain-of-function IDH2 mutations or mutations in the D2HGDH (or L2HGDH) gene.^82^ Thus, while elevated *R-*2HG levels are likely to also affect other 2OG oxygenases, the available evidence suggests that TET2 may be particularly sensitive to cellular inhibition by *R-*2HG (Table S3).

Overall, the results demonstrate that TET1–3 are tractable targets for small molecule inhibition and provide starting points for structure–activity relationship (SAR) studies to obtain selective TET inhibitors. The results highlight the importance of inhibitor cross-screening against TET1–3 and suggest that it should be possible to obtain inhibitors selective for individual TET isozymes. This is demonstrated by the results showing less potent *R-*2HG inhibition of TET1 compared to TET3 and, in particular, TET2. Future work can also focus on determining the relevance of the different sensitivities of the TETs to *R-*2HG in cancer.

## Experimental Section

### General Reagents

Chemicals were from commercial sources unless stated otherwise. 2-[4-(2-Hydroxyethyl)piperazin-1-yl]ethanesulfonic acid (HEPES, Apollo Scientific Limited, Code: BI8181), sodium chloride (Fisher Bioreagents, Cat#: BP358-10), bovine serum albumin (BSA, PerkinElmer, Stabilizer, 7.5% (DTPA-purified BSA), Cat: CR84-100), Tween 20 (Tween 20, Promega, ref#: H5151), disodium 2-oxoglutarate (Sigma, Cat#: K3752–100G), (+)-sodium L-ascorbate (Sigma, Cat#: 11140-50G), (NH_4_)_2_Fe(II)(SO_4_)_2_ (Sigma-Aldrich, cat#: 215406-100G), ethanediaminetetraacetate disodium salt dihydrate (EDTA, Sigma, cat#: E5134-500G), ^5hm^C-specific antibody (Active Motif Cat#: 39769), AlphaScreen beads (PerkinElmer, IgG Detection Kit (Protein A), Cat#: 6760617M, lot: 1571768), Complete EDTA-free Protease Inhibitor Cocktail (Roche Diagnostics Ltd.), ProxiPlate -384 (White, Shallow 384 well, Pinch bar design, part#: 6008280), and DMSO (Fisher Chemical, code: D/4120/PB08). Water, LiChroSolv for LC-MS (VWR cat#: (1.15333.2500)), acetonitrile (LiChroSolv for LC, VWR, cat#: 1.00030.2500). *N*-oxalylglycine disodium was prepared according to the reported procedure.^83,84^

### Inhibitors

IOX1, IOX2, ML324, KDMOAM25, succinate, fumarate, 2OG, dimethylpyridine-2,4-dicarboxylate, dimethyl 2-oxoglutarate, dimethyl succinate, dimethyl fumarate, and DMOG were purchased from Sigma-Aldrich, while IOX3, IOX4, Roxadustat, Vadadustat, Panobinostat, disodium 2*S/2R*-hydroxyglutarate, and FG0041 were obtained from Cayman Chemical Company. 2,4-PDCA (Alfa Aesar), Daprodustat (MedChemExpress), QC6352 (MedChemExpress), Molidustat (Selleck Chem), JIB-04 (Tocris Bioscience), GSK-J1 (Tocris Bioscience), GSK-J4 (Tocris Bioscience), SD70 (Xcess Biosciences), CPI455 (Axon Med Chem), 2′-deoxy-5-methylcytidine (TCI Chemicals), 2′-deoxy-5-(hydroxymethyl)cytidine (TCI Chemicals), and (*R*/*S*)-Octyl-α-hydroxyglutarate (Cambridge Bioscience) were purchased from other commercial sources. All purchased inhibitors were >95% pure as determined by the manufacturer. Key compound purity was verified using analytical HPLC using a UV detector (210 or 254 nm) or CAD detector (Figures S18–S28). NOG was synthesized as described in the materials and methods and has a purity >95% as determined by HPLC. All buffer components were from Sigma-Aldrich unless otherwise stated.

### AlphaScreen Inhibitor Assays

Single-stranded 32-bp DNA ([Biotin]-5′-TCG GAT GTT GTG GGT CAG **C**GC ATG ATA GTG TA-3′), where **C** is ^5m^C or ^5hm^C, was from ATDBio (Oxford, UK). The AlphaScreen General IgG detection kit was from PerkinElmer. All enzyme reactions were performed in assay buffer (50 mM HEPES pH 7.0, 150 mM NaCl, 0.1% BSA, 0.01% Tween 20). TET enzyme in assay buffer (5 μL) was dispensed into ProxiPlate wells and preincubated with inhibitors for 10 min. The DNA-cofactor solution (20 nM DNA, 200 μM sodium L-ascorbate (Asc), 20 μM (NH_4_)_2_Fe(II)(SO_4_)_2_, 20 μM disodium 2OG in buffer) was dispensed (5 μL) to initiate the enzyme reaction and incubated at room temperature. Reactions were quenched with 30 mM EDTA (pH 4.2, 5 μL). The AlphaScreen beads mixture (5 μL) (preincubated (30 min) acceptor and donor AlphaScreen beads (62.5× dilution) with ^5hm^C-specific antibody (Active Motif Cat#: 39769, 1:4000 dilution)] was added with incubation for 45 min, followed by analysis using a PerkinElmer EnVision (2104 Multilabel Reader). Data were processed using Microsoft Excel (2010, version 14.0) and GraphPad Prism 5 (v. 5.04). IC_50_ curves were calculated using GraphPad as log(inhibitor) vs normalized response–variable slope fit. AlphaScreen inhibition assays were conducted at different final protein concentrations and reaction times depending on the construct: TET1_CD_ (10 nM, 30 min), TET2_CD_ (1 nM, 10 min), TET2_CDΔLCI_ (1 nM, 10 min), and TET3_CD_ (10 nM, 10 min).

### SPE-MS Assays

TET2_CDΔLCI_ (0.8 μM) in buffer (50 mM HEPES, pH 7.0) (12.5 μL) was dispensed into inhibitor plates and preincubated for 10 min. The DNA substrate (4 μM 5′-ACC AC^**5m**^**C** GGT GGT-3′ (ATDBio, Oxford, UK), 400 μM sodium L-ascorbate, 20 μM disodium 2-oxoglutarate, and 100 μM (NH_4_)_2_Fe(II)(SO_4_)_2_ in buffer) (12.5 μL) was then dispensed across the plate to initiate the reaction. The enzyme reaction was progressed for 10 min and the reaction was quenched by dispense of 2 mM NOG (25 μL). Samples were analyzed using an Agilent RapidFire RF360 high-throughput sampling robot coupled to an Agilent 6530 accurate-mass quadrupole time-of-flight (Q-TOF) mass spectrometer (see below for details). Data were processed using Masshunter Qualitative Analysis Version B.0 7.00 and Agilent RapidFire Integrator software. IC_50_ values were calculated using GraphPad Prism 5 (v. 5.04) using the model log(inhibitor) vs normalized response–variable slope fit.

### IF Assay for ^5hm^C

Stable U2OS cell lines with tetracycline/Dox-inducible expression of TET catalytic domains (TET1_CD_ (1481–2136 aa), TET2_CD_ (1129–2002 aa), and TET3_CD_ (824–1795 aa) with N-terminal 3 × FLAG) were generated using the Flp-In-T-Rex system (Invitrogen). For TET1_CD_, the construct contained C-terminal GFP, and the catalytically inactive mutant TET1_CD_ (H1672Y and D1674A) was generated. Stable U2OS cells were maintained in DMEM media (Sigma) supplemented with TET system-approved fetal bovine serum (10%, FBS, Clontech), penicillin G (50 IU mL^–1^, Invitrogen), streptomycin (50 μg mL^–1^, Invitrogen), l-glutamine (2 mM, Sigma), blasticidin S (5 μg mL^–1^, Invitrogen), and hygromycin B (150 μg mL^–1^, Roche Applied Science). Prior to transfection, cells were seeded into clear-bottom 96-well plates (CellCarrier Ultra-96, PerkinElmer, 5000 cells per well), in DMEM media supplemented with Tet system-approved FBS (10%), penicillin G (50 IU mL^–1^), streptomycin (50 μg mL^–1^), and l-glutamine (2 mM), and allowed to adhere for 4 h at 37 °C. Cells were then simultaneously dosed with compounds (1% DMSO final concentration) and Dox HCl (1 μg mL^–1^). Cells were then incubated (24 h) at 37 °C, rinsed with phosphate-buffered saline (PBS; Gibco), then fixed in paraformaldehyde for 20 min (4%, Alfa Aesar) and permeabilized with TritonX-100 (0.5%, Sigma, 8 min) in PBS. Prior to blocking, the fixed cells were treated with HCl_(aq)_ (2 N) for 30 min, followed by the removal and neutralization of excess acid on cells with 100 mM Tris HCl (pH 8.0) (Sigma) for 15 min. Cells were then blocked with FBS (3%) in PBS for 30 min, and incubated with a primary antibody solution in FBS (3%) in PBS for 16 h at 4 °C (anti-^5hm^C rabbit polyclonal antibody (Active Motif cat#: 39769, 1:500 dilution), anti-FLAG mouse monoclonal antibody (Sigma F1804, 1:1000 dilution)). The cells were washed with PBS, then incubated with a secondary antibody in FBS (3%) in PBS for 1 h (antirabbit Alexa-647 conjugate and antimouse Alexa-568 conjugate, Life Technologies, 1:500 dilution). Cell nuclei were stained with 4′,6-diamidino-2-phenylindole (DAPI, Invitrogen). Cell imaging was performed using Operetta CLS High-Content Analysis (PerkinElmer) or a Cell Discoverer 7 high-throughput (Zeiss) systems. Images were analyzed using Harmony high-content imaging and analysis software (PerkinElmer). ^5hm^C staining intensities (Alexa Fluor 647) of FLAG-TET expressing cells postcompound treatment were determined (mean fluorescence, s.e.m., and N). Nonlinear regression (four parameters) with constraints (Dox-induced and uninduced DMSO treated WT cells as top and bottom respectively) was used to calculate pIC_50_ (−log (IC_50_/M)) using Prism 7 (Ver 7.01, Graphpad Software, Inc.). Data are given as mean pIC_50_ ± s.e.m. of at least three independent biological replicates. pCC_50_ values calculated based on the cell number (DAPI nuclear staining) within a set of analyzed fields.

### Antibodies Used in the Study

For Western blots, the following primary antibodies were diluted in BSA (5%) in 1× PBS buffer: monoclonal ANTI-FLAG M2 antibody produced in mouse (1:1000, F1804, Merck); anti-β-actin antibody, mouse monoclonal (1:2000, A1978, Merck). HRP goat antirabbit IgG antibody (peroxidase) (1:3000, PI-1000, Vectorlabs), and peroxidase antimouse IgG (H+L) (affinity purified) (1:3000, PI-2000, Vectorlabs) were used as secondary antibodies.

For IF assays, the following primary antibodies were diluted in blocking buffer (FBS 3% in 1× PBS): the 5-hydroxymethylcytosine (^5hm^C) antibody (pAb) (1:500, Active Motif cat#: 39769) and monoclonal ANTI-FLAG M2 antibody produced in mouse (1:500, F1804, Merck). Goat antimouse Alexa Fluor 568 (1:500, A-11031, Invitrogen) and Goat antirabbit Alexa Fluor 647 (1:500, A-21236, Invitrogen) were used as secondary antibodies.

### Recombinant Protein Production

#### TET1_CD_

Recombinant TET1_CD_ (L1418-V2136) with an N-terminal 3 × Flag-tag produced in Sf9 cells was purchased from Epigentek (Cat#: E12002-1). The purity was confirmed by SDS-PAGE (Figure S1).

#### TET2_CD_

Human TET2 (Q969-Y2001) was cloned by ligation-independent cloning into baculovirus transfer vector pFB-CT10HF-LIC (Addgene plasmid #39191). Bacmid DNA was generated by Tn7 recombination in the DH10Bac cell line and transfected into Sf9 cells with the JetPrime reagent (Polyplus). P2 virus was used to infect 6 L of Sf9 cells in Insect-XPRESS medium (Lonza) and TET2_CD_ -His_10_-FLAG protein was produced over 72 h at 27 °C with shaking at 100 rpm. The cells were harvested and suspended in buffer (10 mM imidazole, 0.5 mM tris(2-carboxyethyl)phosphine (TCEP), 500 mM NaCl, 5% glycerol (v/v)] in 100 mM HEPES (pH 7.4)), and protease inhibitors set III (Calbiochem) was added. Cells were lysed using a Dounce homogenizer; the clarified lysates were incubated with Nickel-Sepharose 6 FF (GE Healthcare) for 1 h at 4 °C with rotation. After gravity column transfer, the resin was successively washed with buffer containing increasing imidazole concentrations (50, 70, and 100 mM, and the protein was eluted with buffer containing 250 mM imidazole. Eluted fractions containing purified protein of the desired mass (as judged by SDS-PAGE assay) were concentrated, injected onto a size exclusion Superdex 200 16/60 (GE healthcare) column, and eluted in gel filtration buffer (0.5 mM TCEP, 5% (v/v) glycerol, 500 mM NaCl in 20 mM HEPES (pH 7.4)). Fractions containing TET2_CD_-His_10_-FLAG protein of the correct molecular weight as determined using SDS-PAGE (Figure S1), concentrated, and used for assay development and inhibitor screening.

#### TET3_CD_

The TET3 gene (E824–I1795) was cloned into pHTBV1.1-CT10H–SIII-LIC (C-terminal His_10_-Twin-Strep-TEV) vector. The bacmid containing the TET3 insert (E824–I1795) was used to transfect Sf9 cells (SF9 *Spodoptera frugiperda* cells for virus amplification, Expi293F cells for recombinant protein production) to produce baculovirus for infection. The virus was amplified by growing Sf9 cells (Insect EXPRESS medium from Lonza, 2 × 10^6^ cells mL^–1^) incubated in shaker flasks. Cells were shaken at 90 rpm at 27 °C for 60 h. The cell suspensions were centrifuged (15 min, 800 × *g*, 4 °C); the supernatant containing the amplified TET3 virus was stored at 4 °C. Expi293F GnTI- cell cultures (1 L, 2 × 10^6^ cells mL^–1^) in Freestyle 293 Expression medium (ThermoFisher Scientific) were infected with the baculovirus in the presence of sodium butyrate (5 mM) in a roller bottle (2 L). Cells were shaken in a humidity-controlled incubator for 72 h at 37 °C under a partial CO_2_ (8%) atmosphere. The cell suspensions were harvested by centrifugation (15 min, 800 × *g*, 4 °C) and the cell pellet was resuspended in PBS buffer. After centrifugation (15 min, 800 × *g*, 4 °C), the pellets were stored at −20 °C. Cells were suspended in lysis buffer (50 mM HEPES (pH 7.4), 200 mM NaCl, 20 mM Imidazole, 5% glycerol, 0.5 mM TCEP) and lysed by sonication on ice (2 min, amplitude 35%). The cell lysates were cleared by centrifugation (60 min, 36,000 × *g*, 4 °C). The supernatant was combined with Ni Sepharose (GE Healthcare, 7.5 mL) and loaded onto a gravity flow column. After extensive washing with lysis buffer, the TET3_CD_ His10-tagged protein was eluted using the lysis buffer containing 300 mM imidazole. The eluted fractions were further purified using an AKTA Xpress system combined with an S200 gel filtration column (GE Healthcare) equilibrated with the gel filtration buffer (150 mM NaCl, 0.5 mM TCEP, 5% glycerol in 20 mM HEPES (pH 7.4)). The protein yield was 0.6 mg from 3 L of culture, and the purity was confirmed by SDS-PAGE (Figure S1). The purified protein was stored at 0.2 mg mL^–1^.

#### TET2_CDΔLCI_

A construct encoding for N-terminally His-tagged human TET2 (D1129-G1936 with residues Y1481-N1843 replaced by a 15-residue GS-linker GGGGSGGGGSGGGGS) in the pET-28b vector was kindly provided by the Yanhui Xu laboratory.^25^ The plasmid was transformed and expressed in *Escherichia coli* BL21(DE3) cells. A 6× 10 mL overnight culture was used to inoculate 6 L of Terrific Broth media containing kanamycin (100 μg mL^–1^). Cultures were grown at 37 °C until the OD_600_ reached ∼1.0 after which time, the temperature was adjusted to 18 °C; expression culture was induced with 0.5 mM IPTG and the cells were incubated for 18 h. Cells were harvested by centrifugation (5000 rpm, 30 min, 4 °C), suspended in lysis buffer (500 mM NaCl, 20 mM imidazole, 0.5 mM TCEP, 5% (v/v) glycerol with protease inhibitor cocktail (1:2000, EDTA-Free Protease Inhibitor Cocktail, Roche Diagnostics Ltd.) in 50 mM HEPES (pH 7.4), and lysed by sonication at 4 °C. The lysates were cleared by centrifugation (17,000 rpm, 1 h, 4 °C), and loaded onto a Ni NTA column. After extensively rinsing with lysis buffer, the His_6_- tagged TET2_CDΔLCI_ protein was eluted using the lysis buffer containing 300 mM imidazole. The eluted fractions were further purified using an AKTA Xpress system combined with an S200 gel filtration column equilibrated in the gel filtration buffer (150 mM NaCl, 0.5 mM TCEP, and 5% (v/v) glycerol in 20 mM HEPES (pH 7.4)). The elution volume (92 mL) indicated the protein is monomeric in solution. The final yield was ∼10 mg of His_6_-TET2_CDΔLCI_ from 6 L culture. The purity was confirmed by SDS-PAGE (Figure S1).

### Preparation of Inhibitor Screening Plates for AlphaScreen or SPE-MS Assays

Inhibitors were dry dispensed using an Echo 550 Acoustic dispenser (Labcyte) from inhibitor stock solutions in prepared in DMSO (10 mM). Increments of inhibitor concentrations were made in steps of 2.5 nL and, if necessary, backfilled up to 100 nL (AlphaScreen) or 250 nL (SPE-MS) of total DMSO volume on ProxiPlate (PerkinElmer for AlphaScreen) or PP microplate 384 well (Greiner bio-one, SPE-MS). Intermediate dilutions series were made robotically using a combination of Echo dispensing and MultiDrop (Thermo Scientific, MultiDrop Combi). Every screening plate contained a DMSO control, prequench negative control, and IOX1^45^ as a positive control reference inhibitor. Solutions containing *R* or *S*-2HG, succinate, or fumarate are from purified water source solutions with the solution adjusted to a pH of 7.0. Inhibitor plates containing water-based solutions were allowed to dry (ambient conditions) until all water was evaporated prior to conducting the assay.

### SPE-MS Assay Configuration

Quenched reaction plates (50 μL per well) were transferred to the RapidFire RF360 and samples aspirated under vacuum through a sample loop (10 μL, 400 ms) and loaded onto a SPE cartridge (Agilent, Type A C4, G9203-80103). The C4 SPE was washed with solvent A (6 mM octylammonium acetate in LCMS grade water, 4500 ms, 1.5 mL min^–1^), and the DNA was eluted from the SPE cartridge with solvent B (80% (v/v) acetonitrile in LCMS water, 4500 ms, 1.25 mL min^–1^) into the Agilent QTOF-6530 mass spectrometer. The SPE cartridge was re-equilibrated (solvent A, 500 ms, 1.25 mL min^–1^) prior to the next injection. The cartridge was sequentially washed four times with alternating cycles of water and acetonitrile to remove residual DNA and contaminants between each sample injection. Solid octylammonium acetate (Alfa Aesar) was prepared as described.^86^ The mass spectrometer was operated in negative ESI mode with a nebulizer pressure (40 PSIG); gas temperature (350 °C); drying gas flow rate: (9 L min^–1^); fragmentor voltage (135 V); OCT1 RF Vpp (750 V); and skimmer (65 V).

### Cloning of TET1_CD_, TET2_CD_, and TET3_CD_

DNA sequences encoding for the catalytic domains of WT *TET1*_*CD*_ (1418–2136 aa), *TET2*_*CD*_ (1129–2002 aa), *TET3*_*CD*_ (824–1795 aa), and catalytically inactive mutant *TET1*_*CD*_*MUT* (1418–2136 aa with H1672Y and D1674A) were cloned into pcDNA5–3 × FLAG-FRT/TO and cotransfected with pOG44 Flp-recombinase vector (V600520, Thermo Fisher) in U2OS-TREx cells. Posttransfection (48 h), hygromycin B (100 μg μL) was used for selection; the cells were maintained in Dulbecco’s Modified Eagle Medium (DMEM) supplemented with 10% (v/v) tetracycline-free FBS (TF-FBS), 2 mM l-glutamine, 10 U/mL penicillin/streptomycin. Single-cell colonies were grown and tested for Dox-inducible expression by Western blot analysis (Figure S7). Successful colonies were expanded and frozen. The cells were tested routinely for mycoplasma contamination.

### Synthesis of *N*-Oxalylglycine Diethylate 38^84^

A solution of ethyl 2-aminoacetate hydrochloride (HCl·H-Gly-OEt) (333 mg, 2.4 mmol, 1.1 equiv) in CH_2_Cl_2_ (6.0 mL) under an argon atmosphere was cooled to 0 °C with stirring. Diisopropylethylamine (750 μL, 4.3 mmol, 2.0 equiv) was added, followed by ethyloxalyl chloride (250 μL, 2.2 mmol, 1.0 equiv). The reaction was allowed to warm to room temperature with stirring. After 6 h, the mixture was cooled to 0 °C and quenched by the addition of sat. solution of NH_4_Cl (∼10 mL). The mixture was extracted with ethyl acetate (3 × 30 mL); the combined organic phases were dried over MgSO_4_, filtered and the supernatant was concentrated in vacuo. The crude material was purified by silica gel chromatography using a linear gradient of cyclohexane to ethyl acetate (0–100%) to give a colorless oil (446 mg, 2.2 mmol, 91%). ^1^H NMR 400 MHz (CDCl_3_) δ (ppm): 7.63 (s, 1 H, NH), 4.31 (q, 2 H, *J* = 7.5 Hz, CH_2_CH_3_), 4.19 (q, 2 H, *J* = 7.5 Hz, CH_2_CH_3_), 4.07 (d, 2 H, *J* = 4.5 Hz, NHCH_2_), 1.33 (t, 3 H, *J* = 7.5 Hz, CH_3_), 1.24 (t, 3 H, *J* = 7.5 Hz, CH_3_). ^13^C NMR 101 MHz (CDCl_3_) δ (ppm): 168.7, 160.0, 157.7, 63.3, 61.9, 41.5, 14.1, and 14.0. The obtained NMR data (Figures S14 and S15) are in agreement with reported values.^84^

### Synthesis of NOG Disodium Salt 5^83^

To a solution of diethyl NOG (102 mg, 0.50 mmol, 1.0 equiv) in THF/H_2_O (3.0 mL, 1:1), NaOH (40 mg, 1.0 mmol, 2.0 equiv) was added; the reaction mixture was stirred overnight at room temperature. Volatiles were removed in vacuo and the crude residue was coevaporated twice with ethanol. The crude material was triturated with a small amount of methanol; the so-obtained precipitate was isolated by filtration to yield a white amorphous solid (77 mg, 81%). ^1^H NMR 400 MHz (D_2_O) δ (ppm): 3.79 (s, 2 H, CH_2_).^83^^13^C NMR 126 MHz (D_2_O) δ (ppm): 176.4, 166.0, 164.8, 43.2.^83^ ESI-MS as [M + H]^+^, calc: 148.0; observed: 148.1. The obtained NMR data (Figures S16 and S17) are in agreement with reported values.

## Supporting Information Available

Figures illustrating protein, biochemical and cellular data, details of HPLC and NMR spectra for compounds (PDF)
Molecular formula strings and the associated biochemical and biological data (CSV)

## Abbreviations Used

2OG: 2-oxoglutarate
^5m^C: 5-methylcytosine
^5hm^C: 5-hydroxymethylcytosine
^5f^C: 5-formylcytosine
^5ca^C: 5-carboxylcytosine
^5ox^C: 5-oxidizedcytosines
Asc: L-ascorbate
BER: Base excision repair
CD: Catalytic domain
CLL: Chronic lymphocytic leukemia
CMML: Chronic myelomonocytic leukemia
D2HGDH: D-2-hydroxyglutarate dehydrogenase
DAPI: 4′,6-Diamidino-2-phenylindole dihydrochloride
DMEM: Dulbecco’s Modified Eagle’s Medium
DMOG: Dimethyloxalylglycine
DNMT: DNA methyl transferase
Dox: Doxycycline
DSBH: Double-stranded ß-helix
dsDNA: Double-stranded DNA
*E. coli*: *Escherichia coli*
EMA: European Medicines Agency
FBS: fetal bovine serum
FH: fumerate hydratase
HDAC: histone deacetylases
HEPES: 4-(2-hydroxyethyl)piperazine-1-ethanesulfonic acid
HIF: hypoxia-inducible factor
IDH: isocitrate dehydrogenase
IF: immunofluorescence
JmjC: JumonjiC
L2HGDH: L-2-hydroxyglutarate dehydrogenase
LCI: low complexity insert
KDM: lysine-specific histone demethylase
MDS: myelodysplastic syndrome
MUT: mutant
NOG: *N*-oxalylglycine
N.D.: not determined
N.I.: not inhibitory
PHD: prolyl hydroxylase domain
R-2HG: R-2-hydroxyglutarate
S-2HG: S-2-hydroxyglutarate
SDH: succinate dehydrogenase
s.e.m: standard error of the mean
SPE-MS: solid-phase extraction – mass spectrometry
ssDNA: single stranded DNA
TCA: tricarboxylic acid cycle
TDG: thymidine DNA glycosylase
TET: ten-eleven translocation
WT: wild-type

## References

[ref1] Greenberg M. V. C.; Bourc’his D. The Diverse Roles of DNA Methylation in Mammalian Development and Disease. Nat. Rev. Mol. Cell Biol. 2019, 20 (10), 590–607. 10.1038/s41580-019-0159-6. 31399642

[ref2] Tahiliani M.; Koh K. P.; Shen Y.; Pastor W. A.; Bandukwala H.; Brudno Y.; Agarwal S.; Iyer L. M.; Liu D. R.; Aravind L.; Rao A. Conversion of 5-Methylcytosine to 5-Hydroxymethylcytosine in Mammalian DNA by MLL Partner TET1. Science 2009, 324 (5929), 930–935. 10.1126/science.1170116. 19372391 PMC2715015

[ref3] Kriaucionis S.; Heintz N. The Nuclear DNA Base 5-Hydroxymethylcytosine Is Present in Purkinje Neurons and the Brain. Science 2009, 324 (5929), 929–930. 10.1126/science.1169786. 19372393 PMC3263819

[ref4] Ito S.; Shen L.; Dai Q.; Wu S. C.; Collins L. B.; Swenberg J. A.; He C.; Zhang Y. Tet Proteins Can Convert 5-Methylcytosine to 5-Formylcytosine and 5-Carboxylcytosine. Science 2011, 333 (6047), 1300–1303. 10.1126/science.1210597. 21778364 PMC3495246

[ref5] Cedar H.; Bergman Y. Linking DNA Methylation and Histone Modification: Patterns and Paradigms. Nat. Rev. Genet. 2009, 10 (5), 295–304. 10.1038/nrg2540. 19308066

[ref6] Rasmussen K. D.; Helin K. Role of TET Enzymes in DNA Methylation, Development, and Cancer. Genes Dev. 2016, 30 (7), 733–750. 10.1101/gad.276568.115. 27036965 PMC4826392

[ref7] Thalhammer A.; Hansen A. S.; El-Sagheer A. H.; Brown T.; Schofield C. J. Hydroxylation of Methylated CpG Dinucleotides Reverses Stabilisation of DNA Duplexes by Cytosine 5-Methylation. Chem. Commun. 2011, 47 (18), 5325–5327. 10.1039/c0cc05671e. 21451870

[ref8] Oswald J.; Engemann S.; Lane N.; Mayer W.; Olek A.; Fundele R.; Dean W.; Reik W.; Walter J. Active Demethylation of the Paternal Genome in the Mouse Zygote. Curr. Biol. 2000, 10 (8), 475–478. 10.1016/S0960-9822(00)00448-6. 10801417

[ref9] Rougier N.; Bourchis D.; Molina Gomes D.; Niveleau A.; Plachot M.; Pàldi A.; Viegas-Péquignot E. Chromosome Methylation Patterns during Mammalian Preimplantation Development. Genes Dev. 1998, 12 (14), 2108–2113. 10.1101/gad.12.14.2108. 9679055 PMC317005

[ref10] Wu X.; Zhang Y. TET-Mediated Active DNA Demethylation: Mechanism Function and Beyond. Nat. Rev. Genet. 2017, 18 (9), 517–534. 10.1038/nrg.2017.33. 28555658

[ref11] Iwan K.; Rahimoff R.; Kirchner A.; Spada F.; Schröder A. S.; Kosmatchev O.; Ferizaj S.; Steinbacher J.; Parsa E.; Müller M.; Carell T. 5-Formylcytosine to Cytosine Conversion by C-C Bond Cleavage in Vivo. Nat. Chem. Biol. 2018, 14 (1), 72–78. 10.1038/nchembio.2531. 29176672

[ref12] Bachman M.; Uribe-Lewis S.; Yang X.; Williams M.; Murrell A.; Balasubramanian S. 5-Hydroxymethylcytosine Is a Predominantly Stable DNA Modification. Nat. Chem. 2014, 6 (12), 1049–1055. 10.1038/nchem.2064. 25411882 PMC4382525

[ref13] Bachman M.; Uribe-Lewis S.; Yang X.; Burgess H. E.; Iurlaro M.; Reik W.; Murrell A.; Balasubramanian S. 5-Formylcytosine Can Be a Stable DNA Modification in Mammals. Nat. Chem. Biol. 2015, 11 (8), 555–557. 10.1038/nchembio.1848. 26098680 PMC5486442

[ref14] Ren R.; Horton J. R.; Zhang X.; Blumenthal R. M.; Cheng X. Detecting and Interpreting DNA Methylation Marks. Curr. Opin. Struct. Biol. 2018, 53, 88–99. 10.1016/j.sbi.2018.06.004. 30031306 PMC6322410

[ref15] Raiber E.-A.; Portella G.; Martínez Cuesta S.; Hardisty R.; Murat P.; Li Z.; Iurlaro M.; Dean W.; Spindel J.; Beraldi D.; Liu Z.; Dawson M. A.; Reik W.; Balasubramanian S. 5-Formylcytosine Organizes Nucleosomes and Forms Schiff Base Interactions with Histones in Mouse Embryonic Stem Cells. Nat. Chem. 2018, 10 (12), 1258–1266. 10.1038/s41557-018-0149-x. 30349137

[ref16] Raiber E.-A.; Murat P.; Chirgadze D. Y.; Beraldi D.; Luisi B. F.; Balasubramanian S. 5-Formylcytosine Alters the Structure of the DNA Double Helix. Nat. Struct. Mol. Biol. 2015, 22 (1), 44–49. 10.1038/nsmb.2936. 25504322 PMC4287393

[ref17] Hardwick J. S.; Ptchelkine D.; El-Sagheer A. H.; Tear I.; Singleton D.; Phillips S. E. V; Lane A. N.; Brown T. 5-Formylcytosine Does Not Change the Global Structure of DNA. Nat. Struct. Mol. Biol. 2017, 24 (6), 544–552. 10.1038/nsmb.3411. 28504696 PMC5747368

[ref18] Lercher L.; McDonough M. A.; El-Sagheer A. H.; Thalhammer A.; Kriaucionis S.; Brown T.; Schofield C. J. Structural Insights into How 5-Hydroxymethylation Influences Transcription Factor Binding. Chem. Commun. 2014, 50 (15), 1794–1796. 10.1039/C3CC48151D. 24287551

[ref19] Hu L.; Li Z.; Cheng J.; Rao Q.; Gong W.; Liu M.; Shi Y. G.; Zhu J.; Wang P.; Xu Y. Crystal Structure of TET2-DNA Complex: Insight into TET-Mediated 5mC Oxidation. Cell 2013, 155 (7), 1545–1555. 10.1016/j.cell.2013.11.020. 24315485

[ref20] Rose N. R.; McDonough M. A.; King O. N. F.; Kawamura A.; Schofield C. J. Inhibition of 2-Oxoglutarate Dependent Oxygenases. Chem. Soc. Rev. 2011, 40 (8), 4364–4397. 10.1039/c0cs00203h. 21390379

[ref21] Brasnett A.; Pfeffer I.; Brewitz L.; Chowdhury R.; Nakashima Y.; Tumber A.; McDonough M. A.; Schofield C. J. Human Oxygenase Variants Employing a Single Protein FeII Ligand Are Catalytically Active. Angew. Chemie Int. Ed. 2021, 60 (26), 14657–14663. 10.1002/anie.202103711. PMC8252765 33887099

[ref22] Xu C.; Liu K.; Lei M.; Yang A.; Li Y.; Hughes T. R.; Min J. DNA Sequence Recognition of Human CXXC Domains and Their Structural Determinants. Structure 2018, 26 (1), 85–95. 10.1016/j.str.2017.11.022. 29276034

[ref23] Ko M.; An J.; Bandukwala H. S.; Chavez L.; Äijö T.; Pastor W. A.; Segal M. F.; Li H.; Koh K. P.; Lähdesmäki H.; Hogan P. G.; Aravind L.; Rao A. Modulation of TET2 Expression and 5-Methylcytosine Oxidation by the CXXC Domain Protein IDAX. Nature 2013, 497 (7447), 122–126. 10.1038/nature12052. 23563267 PMC3643997

[ref24] Pfaffeneder T.; Spada F.; Wagner M.; Brandmayr C.; Laube S. K.; Eisen D.; Truss M.; Steinbacher J.; Hackner B.; Kotljarova O.; Schuermann D.; Michalakis S.; Kosmatchev O.; Schiesser S.; Steigenberger B.; Raddaoui N.; Kashiwazaki G.; Müller U.; Spruijt C. G.; Vermeulen M.; Leonhardt H.; Schär P.; Müller M.; Carell T. Tet Oxidizes Thymine to 5-Hydroxymethyluracil in Mouse Embryonic Stem Cell DNA. Nat. Chem. Biol. 2014, 10 (7), 574–581. 10.1038/nchembio.1532. 24838012

[ref25] Hu L.; Lu J.; Cheng J.; Rao Q.; Li Z.; Hou H.; Lou Z.; Zhang L.; Li W.; Gong W.; Liu M.; Sun C.; Yin X.; Li J.; Tan X.; Wang P.; Wang Y.; Fang D.; Cui Q.; Yang P.; He C.; Jiang H.; Luo C.; Xu Y. Structural Insight into Substrate Preference for TET-Mediated Oxidation. Nature 2015, 527 (7576), 118–122. 10.1038/nature15713. 26524525

[ref26] Schröder A. S.; Parsa E.; Iwan K.; Traube F. R.; Wallner M.; Serdjukow S.; Carell T. 2′-(R)-Fluorinated MC, HmC, FC and CaC Triphosphates Are Substrates for DNA Polymerases and TET-Enzymes. Chem. Commun. 2016, 52 (100), 14361–14364. 10.1039/C6CC07517G. 27905578

[ref27] DeNizio J. E.; Liu M. Y.; Leddin E. M.; Cisneros G. A.; Kohli R. M. Selectivity and Promiscuity in TET-Mediated Oxidation of 5-Methylcytosine in DNA and RNA. Biochemistry 2019, 58 (5), 411–421. 10.1021/acs.biochem.8b00912. 30387995 PMC6363868

[ref28] Delhommeau F.; Dupont S.; Della Valle V.; James C.; Trannoy S.; Massé A.; Kosmider O.; Le Couedic J. P.; Robert F.; Alberdi A.; Lécluse Y.; Plo I.; Dreyfus F. J.; Marzac C.; Casadevall N.; Lacombe C.; Romana S. P.; Dessen P.; Soulier J.; Viguié F.; Fontenay M.; Vainchenker W.; Bernard O. A. Mutation in TET2 in Myeloid Cancers. N. Engl. J. Med. 2009, 360 (22), 2289–2301. 10.1056/NEJMoa0810069. 19474426

[ref29] Huang H.; Jiang X.; Li Z.; Li Y.; Song C.-X.; He C.; Sun M.; Chen P.; Gurbuxani S.; Wang J.; Hong G.-M.; Elkahloun A. G.; Arnovitz S.; Wang J.; Szulwach K.; Lin L.; Street C.; Wunderlich M.; Dawlaty M.; Neilly M. B.; Jaenisch R.; Yang F.-C.; Mulloy J. C.; Jin P.; Liu P. P.; Rowley J. D.; Xu M.; He C.; Chen J. TET1 Plays an Essential Oncogenic Role in MLL-Rearranged Leukemia. Proc. Natl. Acad. Sci. U. S. A. 2013, 110 (29), 11994–11999. 10.1073/pnas.1310656110. 23818607 PMC3718141

[ref30] Weissmann S.; Alpermann T.; Grossmann V.; Kowarsch A.; Nadarajah N.; Eder C.; Dicker F.; Fasan A.; Haferlach C.; Haferlach T.; Kern W.; Schnittger S.; Kohlmann A. Landscape of TET2 Mutations in Acute Myeloid Leukemia. Leukemia 2012, 26 (5), 934–942. 10.1038/leu.2011.326. 22116554

[ref31] Figueroa M. E.; Abdel-Wahab O.; Lu C.; Ward P. S.; Patel J.; Shih A.; Li Y.; Bhagwat N.; Vasanthakumar A.; Fernandez H. F.; Tallman M. S.; Sun Z.; Wolniak K.; Peeters J. K.; Liu W.; Choe S. E.; Fantin V. R.; Paietta E.; Löwenberg B.; Licht J. D.; Godley L. A.; Delwel R.; Valk P. J. M.; Thompson C. B.; Levine R. L.; Melnick A. Leukemic IDH1 and IDH2 Mutations Result in a Hypermethylation Phenotype, Disrupt TET2 Function, and Impair Hematopoietic Differentiation. Cancer Cell 2010, 18 (6), 553–567. 10.1016/j.ccr.2010.11.015. 21130701 PMC4105845

[ref32] Choi C.; Ganji S. K.; DeBerardinis R. J.; Hatanpaa K. J.; Rakheja D.; Kovacs Z.; Yang X. L.; Mashimo T.; Raisanen J. M.; Marin-Valencia I.; Pascual J. M.; Madden C. J.; Mickey B. E.; Malloy C. R.; Bachoo R. M.; Maher E. A. 2-Hydroxyglutarate Detection by Magnetic Resonance Spectroscopy in IDH-Mutated Patients with Gliomas. Nat. Med. 2012, 18 (4), 624–629. 10.1038/nm.2682. 22281806 PMC3615719

[ref33] Dang L.; White D. W.; Gross S.; Bennett B. D.; Bittinger M. A.; Driggers E. M.; Fantin V. R.; Jang H. G.; Jin S.; Keenan M. C.; Marks K. M.; Prins R. M.; Ward P. S.; Yen K. E.; Liau L. M.; Rabinowitz J. D.; Cantley L. C.; Thompson C. B.; Vander Heiden M. G.; Su S. M. Cancer-Associated IDH1 Mutations Produce 2-Hydroxyglutarate. Nature 2009, 462 (7274), 739–744. 10.1038/nature08617. 19935646 PMC2818760

[ref34] Xu W.; Yang H.; Liu Y.; Yang Y.; Wang P. P.; Kim S.-H.; Ito S.; Yang C.; Wang P. P.; Xiao M.-T.; Liu L.; Jiang W.; Liu J.; Zhang J.; Wang B.; Frye S.; Zhang Y.; Xu Y.; Lei Q.; Guan K.-L.; Zhao S.; Xiong Y. Oncometabolite 2-Hydroxyglutarate Is a Competitive Inhibitor of α-Ketoglutarate-Dependent Dioxygenases. Cancer Cell 2011, 19 (1), 17–30. 10.1016/j.ccr.2010.12.014. 21251613 PMC3229304

[ref35] Guan Y.; Tiwari A. D.; Phillips J. G.; Hasipek M.; Grabowski D. R.; Pagliuca S.; Gopal P.; Kerr C. M.; Adema V.; Radivoyevitch T.; Parker Y.; Lindner D. J.; Meggendorfer M.; Abazeed M.; Sekeres M. A.; Mian O. Y.; Haferlach T.; Maciejewski J. P.; Jha B. K. A Therapeutic Strategy for Preferential Targeting of TET2-Mutant and TET Dioxygenase-Deficient Cells in Myeloid Neoplasms. Blood cancer Discovery 2021, 2 (2), 146–161. 10.1158/2643-3230.BCD-20-0173. 33681816 PMC7935131

[ref36] Fraietta J. A.; Nobles C. L.; Sammons M. A.; Lundh S.; Carty S. A.; Reich T. J.; Cogdill A. P.; Morrissette J. J. D.; DeNizio J. E.; Reddy S.; Hwang Y.; Gohil M.; Kulikovskaya I.; Nazimuddin F.; Gupta M.; Chen F.; Everett J. K.; Alexander K. A.; Lin-Shiao E.; Gee M. H.; Liu X.; Young R. M.; Ambrose D.; Wang Y.; Xu J.; Jordan M. S.; Marcucci K. T.; Levine B. L.; Garcia K. C.; Zhao Y.; Kalos M.; Porter D. L.; Kohli R. M.; Lacey S. F.; Berger S. L.; Bushman F. D.; June C. H.; Melenhorst J. J. Disruption of TET2 Promotes the Therapeutic Efficacy of CD19-Targeted T Cells. Nature 2018, 558 (7709), 307–312. 10.1038/s41586-018-0178-z. 29849141 PMC6320248

[ref37] Nishio K.; Belle R.; Katoh T.; Kawamura A.; Sengoku T.; Hanada K.; Ohsawa N.; Shirouzu M.; Yokoyama S.; Suga H. Thioether Macrocyclic Peptides Selected against TET1 Compact Catalytic Domain Inhibit TET1 Catalytic Activity. ChemBioChem. 2018, 19 (9), 979–985. 10.1002/cbic.201800047. 29665240

[ref38] Belle R.; Kawamura A.; Arimondo P. B. Chemical Compounds Targeting DNA Methylation and Hydroxymethylation. In Topics in Medicinal Chemistry; Mai A., Ed.; Springer Nature: 2020; Vol. 33, pp 255–286, 10.1007/7355_2019_76.

[ref39] Chua G. N. L.; Wassarman K. L.; Sun H.; Alp J. A.; Jarczyk E. I.; Kuzio N. J.; Bennett M. J.; Malachowsky B. G.; Kruse M.; Kennedy A. J. Cytosine-Based TET Enzyme Inhibitors. ACS Med. Chem. Lett. 2019, 10 (2), 180–185. 10.1021/acsmedchemlett.8b00474. 30783500 PMC6378777

[ref40] Singh A. K.; Zhao B.; Liu X.; Wang X.; Li H.; Qin H.; Wu X.; Ma Y.; Horne D.; Yu X. Selective Targeting of TET Catalytic Domain Promotes Somatic Cell Reprogramming. Proc. Natl. Acad. Sci. U. S. A. 2020, 117 (7), 3621–3626. 10.1073/pnas.1910702117. 32024762 PMC7035619

[ref41] Marholz L. J.; Wang W.; Zheng Y.; Wang X. A Fluorescence Polarization Biophysical Assay for the Naegleria DNA Hydroxylase Tet1. ACS Med. Chem. Lett. 2016, 7 (2), 167–171. 10.1021/acsmedchemlett.5b00366. 27980707 PMC5141568

[ref42] Sudhamalla B.; Dey D.; Breski M.; Islam K. A Rapid Mass Spectrometric Method for the Measurement of Catalytic Activity of Ten-Eleven Translocation Enzymes. Anal. Biochem. 2017, 534, 28–35. 10.1016/j.ab.2017.06.011. 28647531 PMC5781229

[ref43] Laukka T.; Mariani C. J.; Ihantola T.; Cao J. Z.; Hokkanen J.; Kaelin W. G.; Godley L. A.; Koivunen P. Fumarate and Succinate Regulate Expression of Hypoxia-Inducible Genes via TET Enzymes. J. Biol. Chem. 2016, 291 (8), 4256–4265. 10.1074/jbc.M115.688762. 26703470 PMC4759199

[ref44] Rose N. R.; Ng S. S.; Mecinović J.; Liénard B. M. R.; Bello S. H.; Sun Z.; McDonough M. A.; Oppermann U.; Schofield C. J. Inhibitor Scaffolds for 2-Oxoglutarate-Dependent Histone Lysine Demethylases. J. Med. Chem. 2008, 51 (22), 7053–7056. 10.1021/jm800936s. 18942826

[ref45] Hopkinson R. J.; Tumber A.; Yapp C.; Chowdhury R.; Aik W. S.; Che K. H.; Li X. S.; Kristensen J. B. L.; King O. N. F.; Chan M. C.; Yeoh K. K.; Choi H.; Walport L. J.; Thinnes C. C.; Bush J. T.; Lejeune C.; Rydzik A. M.; Rose N. R.; Bagg E. A.; McDonough M. A.; Krojer T. J.; Yue W. W.; Ng S. S.; Olsen L.; Brennan P. E.; Oppermann U.; Müller S.; Klose R. J.; Ratcliffe P. J.; Schofield C. J.; Kawamura A. 5-Carboxy-8-Hydroxyquinoline Is a Broad Spectrum 2-Oxoglutarate Oxygenase Inhibitor Which Causes Iron Translocation. Chem. Sci. 2013, 4 (8), 3110–3117. 10.1039/c3sc51122g. 26682036 PMC4678600

[ref46] Chan M. C.; Holt-Martyn J. P.; Schofield C. J.; Ratcliffe P. J. Pharmacological Targeting of the HIF Hydroxylases - A New Field in Medicine Development. Mol. Aspects Med. 2016, 47–48, 54–75. 10.1016/j.mam.2016.01.001. 26791432

[ref47] Yeh T. L.; Leissing T. M.; Abboud M. I.; Thinnes C. C.; Atasoylu O.; Holt-Martyn J. P.; Zhang D.; Tumber A.; Lippl K.; Lohans C. T.; Leung I. K. H.; Morcrette H.; Clifton I. J.; Claridge T. D. W.; Kawamura A.; Flashman E.; Lu X.; Ratcliffe P. J.; Chowdhury R.; Pugh C. W.; Schofield C. J. Molecular and Cellular Mechanisms of HIF Prolyl Hydroxylase Inhibitors in Clinical Trials. Chem. Sci. 2017, 8 (11), 7651–7668. 10.1039/C7SC02103H. 29435217 PMC5802278

[ref48] McAllister T. E.; England K. S.; Hopkinson R. J.; Brennan P. E.; Kawamura A.; Schofield C. J. Recent Progress in Histone Demethylase Inhibitors. J. Med. Chem. 2016, 59 (4), 1308–1329. 10.1021/acs.jmedchem.5b01758. 26710088

[ref49] Hirota K. HIF-α Prolyl Hydroxylase Inhibitors and Their Implications for Biomedicine: A Comprehensive Review. Biomedicines 2021, 9 (5), 468 10.3390/biomedicines9050468. 33923349 PMC8146675

[ref50] Wang L.; Chang J.; Varghese D.; Dellinger M.; Kumar S.; Best A. M.; Ruiz J.; Bruick R.; Peña-Llopis S.; Xu J.; Babinski D. J.; Frantz D. E.; Brekken R. A.; Quinn A. M.; Simeonov A.; Easmon J.; Martinez E. D. A Small Molecule Modulates Jumonji Histone Demethylase Activity and Selectively Inhibits Cancer Growth. Nat. Commun. 2013, 4 (1), 2035 10.1038/ncomms3035. 23792809 PMC3724450

[ref51] Dobrynin G.; McAllister T. E.; Leszczynska K. B.; Ramachandran S.; Krieg A. J.; Kawamura A.; Hammond E. M. KDM4A Regulates HIF-1 Levels through H3K9me3. Sci. Rep. 2017, 7 (1), 11094 10.1038/s41598-017-11658-3. 28894274 PMC5593970

[ref52] Hatch S. B.; Yapp C.; Montenegro R. C.; Savitsky P.; Gamble V.; Tumber A.; Ruda G. F.; Bavetsias V.; Fedorov O.; Atrash B.; Raynaud F.; Lanigan R.; Carmichael L.; Tomlin K.; Burke R.; Westaway S. M.; Brown J. A.; Prinjha R. K.; Martinez E. D.; Oppermann U.; Schofield C. J.; Bountra C.; Kawamura A.; Blagg J.; Brennan P. E.; Rossanese O.; Müller S. Assessing Histone Demethylase Inhibitors in Cells: Lessons Learned. Epigenetics Chromatin 2017, 10 (1), 9 10.1186/s13072-017-0116-6. 28265301 PMC5333395

[ref53] Kruidenier L.; Chung C. W.; Cheng Z.; Liddle J.; Che K.; Joberty G.; Bantscheff M.; Bountra C.; Bridges A.; Diallo H.; Eberhard D.; Hutchinson S.; Jones E.; Katso R.; Leveridge M.; Mander P. K.; Mosley J.; Ramirez-Molina C.; Rowland P.; Schofield C. J.; Sheppard R. J.; Smith J. E.; Swales C.; Tanner R.; Thomas P.; Tumber A.; Drewes G.; Oppermann U.; Patel D. J.; Lee K.; Wilson D. M. A Selective Jumonji H3K27 Demethylase Inhibitor Modulates the Proinflammatory Macrophage Response. Nature 2012, 488 (7411), 404–408. 10.1038/nature11262. 22842901 PMC4691848

[ref54] Heinemann B.; Nielsen J. M.; Hudlebusch H. R.; Lees M. J.; Larsen D. V.; Boesen T.; Labelle M.; Gerlach L. O.; Birk P.; Helin K. Inhibition of Demethylases by GSK-J1/J4. Nature 2014, 514 (7520), E1–E2. 10.1038/nature13688. 25279926

[ref55] Metzger E.; Stepputtis S. S.; Strietz J.; Preca B. T.; Urban S.; Willmann D.; Allen A.; Zenk F.; Iovino N.; Bronsert P.; Proske A.; Follo M.; Boerries M.; Stickeler E.; Xu J.; Wallace M. B.; Stafford J. A.; Kanouni T.; Maurer J.; Schüle R. KDM4 Inhibition Targets Breast Cancer Stem–like Cells. Cancer Res. 2017, 77 (21), 5900–5912. 10.1158/0008-5472.CAN-17-1754. 28883001

[ref56] Chen Y. K.; Bonaldi T.; Cuomo A.; Del Rosario J. R.; Hosfield D. J.; Kanouni T.; Kao S.; Lai C.; Lobo N. A.; Matuszkiewicz J.; McGeehan A.; O’Connell S. M.; Shi L.; Stafford J. A.; Stansfield R. K.; Veal J. M.; Weiss M. S.; Yuen N. Y.; Wallace M. B. Design of KDM4 Inhibitors with Antiproliferative Effects in Cancer Models. ACS Med. Chem. Lett. 2017, 8 (8), 869–874. 10.1021/acsmedchemlett.7b00220. 28835804 PMC5554903

[ref57] Tumber A.; Nuzzi A.; Hookway E. S.; Hatch S. B.; Velupillai S.; Johansson C.; Kawamura A.; Savitsky P.; Yapp C.; Szykowska A.; Wu N.; Bountra C.; Strain-Damerell C.; Burgess-Brown N. A.; Ruda G. F.; Fedorov O.; Munro S.; England K. S.; Nowak R. P.; Schofield C. J.; La Thangue N. B.; Pawlyn C.; Davies F.; Morgan G.; Athanasou N.; Müller S.; Oppermann U.; Brennan P. E. Potent and Selective KDM5 Inhibitor Stops Cellular Demethylation of H3K4me3 at Transcription Start Sites and Proliferation of MM1S Myeloma Cells. Cell Chem. Biol. 2017, 24 (3), 371–380. 10.1016/j.chembiol.2017.02.006. 28262558 PMC5361737

[ref58] Vinogradova M.; Gehling V. S.; Gustafson A.; Arora S.; Tindell C. A.; Wilson C.; Williamson K. E.; Guler G. D.; Gangurde P.; Manieri W.; Busby J.; Flynn E. M.; Lan F.; Kim H. J.; Odate S.; Cochran A. G.; Liu Y.; Wongchenko M.; Yang Y.; Cheung T. K.; Maile T. M.; Lau T.; Costa M.; Hegde G. V.; Jackson E.; Pitti R.; Arnott D.; Bailey C.; Bellon S.; Cummings R. T.; Albrecht B. K.; Harmange J. C.; Kiefer J. R.; Trojer P.; Classon M. An Inhibitor of KDM5 Demethylases Reduces Survival of Drug-Tolerant Cancer Cells. Nat. Chem. Biol. 2016, 12 (7), 531–538. 10.1038/nchembio.2085. 27214401

[ref59] Jin C.; Yang L.; Xie M.; Lin C.; Merkurjev D.; Yang J. C.; Tanasa B.; Oh S.; Zhang J.; Ohgi K. A.; Zhou H.; Li W.; Evans C. P.; Ding S.; Rosenfeld M. G. Chem-Seq Permits Identification of Genomic Targets of Drugs against Androgen Receptor Regulation Selected by Functional Phenotypic Screens. Proc. Natl. Acad. Sci. U. S. A. 2014, 111 (25), 9235–9240. 10.1073/pnas.1404303111. 24928520 PMC4078819

[ref60] Hamada S.; Suzuki T.; Mino K.; Koseki K.; Oehme F.; Flamme I.; Ozasa H.; Itoh Y.; Ogasawara D.; Komaarashi H.; Kato A.; Tsumoto H.; Nakagawa H.; Hasegawa M.; Sasaki R.; Mizukami T.; Miyata N. Design, Synthesis, Enzyme-Inhibitory Activity, and Effect on Human Cancer Cells of a Novel Series of Jumonji Domain-Containing Protein 2 Histone Demethylase Inhibitors. J. Med. Chem. 2010, 53 (15), 5629–5638. 10.1021/jm1003655. 20684604

[ref61] Suzuki T.; Ozasa H.; Itoh Y.; Zhan P.; Sawada H.; Mino K.; Walport L.; Ohkubo R.; Kawamura A.; Yonezawa M.; Tsukada Y.; Tumber A.; Nakagawa H.; Hasegawa M.; Sasaki R.; Mizukami T.; Schofield C. J.; Miyata N. Identification of the KDM2/7 Histone Lysine Demethylase Subfamily Inhibitor and Its Antiproliferative Activity. J. Med. Chem. 2013, 56 (18), 7222–7231. 10.1021/jm400624b. 23964788 PMC3929130

[ref62] Belle R.; Al Temimi A. H. K.; Kumar K.; Pieters B. J. G. E.; Tumber A.; Dunford J. E.; Johansson C.; Oppermann U.; Brown T.; Schofield C. J.; Hopkinson R. J.; Paton R. S.; Kawamura A.; Mecinović J. Investigating D-Lysine Stereochemistry for Epigenetic Methylation Demethylation and Recognition. Chem. Commun. 2017, 53 (99), 13264–13267. 10.1039/C7CC08028J. PMC6345366 29186216

[ref63] Vas G.; Conkrite K.; Amidon W.; Qian Y.; Bánki K.; Perl A. Study of Transaldolase Deficiency in Urine Samples by Capillary LC-MS/MS. J. Mass Spectrom. 2006, 41 (4), 463–469. 10.1002/jms.1004. 16470722 PMC3127395

[ref64] Laukka T.; Myllykoski M.; Looper R. E.; Koivunen P. Cancer-Associated 2-Oxoglutarate Analogues Modify Histone Methylation by Inhibiting Histone Lysine Demethylases. J. Mol. Biol. 2018, 430 (18), 3081–3092. 10.1016/j.jmb.2018.06.048. 29981745

[ref65] Thirstrup K.; Christensen S.; Mo̷ller H. A.; Ritzén A.; Bergström A. L.; Sager T. N.; Jensen H. S. Endogenous 2-Oxoglutarate Levels Impact Potencies of Competitive HIF Prolyl Hydroxylase Inhibitors. Pharmacol. Res. 2011, 64 (3), 268–273. 10.1016/J.PHRS.2011.03.017. 21504793

[ref66] Siess E. A.; Brocks D. G.; Lattke H. K.; Wieland O. H. Effect of Glucagon on Metabolite Compartmentation in Isolated Rat Liver Cells during Gluconeogenesis from Lactate. Biochem. J. 1977, 166 (2), 225–235. 10.1042/bj1660225. 199159 PMC1164999

[ref67] Gupta N.; Wish J. B. Hypoxia-Inducible Factor Prolyl Hydroxylase Inhibitors: A Potential New Treatment for Anemia in Patients With CKD. Am. J. Kidney Dis. 2017, 69 (6), 815–826. 10.1053/j.ajkd.2016.12.011. 28242135

[ref68] Thinnes C. C.; Tumber A.; Yapp C.; Scozzafava G.; Yeh T.; Chan M. C.; Tran T. A.; Hsu K.; Tarhonskaya H.; Walport L. J.; Wilkins S. E.; Martinez E. D.; Müller S.; Pugh C. W.; Ratcliffe P. J.; Brennan P. E.; Kawamura A.; Schofield C. J. Betti Reaction Enables Efficient Synthesis of 8-Hydroxyquinoline Inhibitors of 2-Oxoglutarate Oxygenases. Chem. Commun. 2015, 51 (84), 15458–15461. 10.1039/C5CC06095H. 26345662

[ref69] Zentner G. E.; Henikoff S. Regulation of Nucleosome Dynamics by Histone Modifications. Nat. Struct. Mol. Biol. 2013, 20 (3), 259–266. 10.1038/nsmb.2470. 23463310

[ref70] Islam M. S.; Thinnes C. C.; Holt-Martyn J. P.; Chowdhury R.; McDonough M. A.; Schofield C. J. Inhibition of JMJD6 by 2-Oxoglutarate Mimics. ChemMedChem. 2022, 17 (1), e202100398 10.1002/cmdc.202100398. 34581506 PMC9299220

[ref71] Tomlinson I. P. M.; Alam N. A.; Rowan A. J.; Barclay E.; Jaeger E. E. M.; Kelsell D.; Leigh I.; Gorman P.; Lamlum H.; Rahman S.; Roylance R. R.; Olpin S.; Bevan S.; Barker K.; Hearle N.; Houlston R. S.; Kiuru M.; Lehtonen R.; Karhu A.; Vilkki S.; Laiho P.; Eklund C.; Vierimaa O.; Aittomäki K.; Hietala M.; Sistonen P.; Paetau A.; Salovaara R.; Herva R.; Launonen V.; Aaltonen L. A. Germline Mutations in FH Predispose to Dominantly Inherited Uterine Fibroids, Skin Leiomyomata and Papillary Renal Cell Cancer the Multiple Leiomyoma Consortium. Nat. Genet. 2002, 30 (4), 406–410. 10.1038/ng849. 11865300

[ref72] Nowicki S.; Gottlieb E. Oncometabolites: Tailoring Our Genes. FEBS J. 2015, 282 (15), 2796–2805. 10.1111/febs.13295. 25864878 PMC4676302

[ref73] Hoekstra A. S.; de Graaff M. A.; Briaire-de Bruijn I. H.; Ras C.; Seifar R. M.; van Minderhout I.; Cornelisse C. J.; Hogendoorn P. C. W.; Breuning M. H.; Suijker J.; Korpershoek E.; Kunst H. P. M.; Frizzell N.; Devilee P.; Bayley J. P.; Bovée J. V. M. G. Inactivation of SDH and FH Cause Loss of 5hmC and Increased H3K9me3 in Paraganglioma/Pheochromocytoma and Smooth Muscle Tumors. Oncotarget 2015, 6 (36), 38777–38788. 10.18632/oncotarget.6091. 26472283 PMC4770736

[ref74] Xiao M.; Yang H.; Xu W.; Ma S.; Lin H.; Zhu H.; Liu L.; Liu Y.; Yang C.; Xu Y.; Zhao S.; Ye D.; Xiong Y.; Guan K. L. Inhibition of α-KG-Dependent Histone and DNA Demethylases by Fumarate and Succinate That Are Accumulated in Mutations of FH and SDH Tumor Suppressors. Genes Dev. 2012, 26 (12), 1326–1338. 10.1101/gad.191056.112. 22677546 PMC3387660

[ref75] Chowdhury R.; Yeoh K. K.; Tian Y. M.; Hillringhaus L.; Bagg E. A.; Rose N. R.; Leung I. K. H.; Li X. S.; Woon E. C. Y.; Yang M.; McDonough M. A.; King O. N.; Clifton I. J.; Klose R. J.; Claridge T. D. W.; Ratcliffe P. J.; Schofield C. J.; Kawamura A. The Oncometabolite 2-Hydroxyglutarate Inhibits Histone Lysine Demethylases. EMBO Rep. 2011, 12 (5), 463–469. 10.1038/embor.2011.43. 21460794 PMC3090014

[ref76] Koivunen P.; Lee S.; Duncan C. G.; Lopez G.; Lu G.; Ramkissoon S.; Losman J. A.; Joensuu P.; Bergmann U.; Gross S.; Travins J.; Weiss S.; Looper R.; Ligon K. L.; Verhaak R. G. W.; Yan H.; Kaelin W. G. Transformation by the (R)-Enantiomer of 2-Hydroxyglutarate Linked to EGLN Activation. Nature 2012, 483 (7390), 484–488. 10.1038/nature10898. 22343896 PMC3656605

[ref77] Tarhonskaya H.; Nowak R. P.; Johansson C.; Szykowska A.; Tumber A.; Hancock R. L.; Lang P.; Flashman E.; Oppermann U.; Schofield C. J.; Kawamura A. Studies on the Interaction of the Histone Demethylase KDM5B with Tricarboxylic Acid Cycle Intermediates. J. Mol. Biol. 2017, 429 (19), 2895–2906. 10.1016/j.jmb.2017.08.007. 28827149 PMC5636616

[ref78] Tarhonskaya H.; Rydzik A. M.; Leung I. K. H.; Loik N. D.; Chan M. C.; Kawamura A.; McCullagh J. S. O.; Claridge T. D. W.; Flashman E.; Schofield C. J. Non-Enzymatic Chemistry Enables 2-Hydroxyglutarate-Mediated Activation of 2-Oxoglutarate Oxygenases. Nat. Commun. 2014, 5 (1), 1–10. 10.1038/ncomms4423. PMC3959194 24594748

[ref79] Du X.; Hu H. The Roles of 2-Hydroxyglutarate. Front. Cell Dev. Biol. 2021, 9, 651317 10.3389/fcell.2021.651317. 33842477 PMC8033037

[ref80] Achouri Y.; Noël G.; Vertommen D.; Rider M. H.; Veiga-Da-Cunha M.; Van Schaftingen E. Identification of a Dehydrogenase Acting on D-2-Hydroxyglutarate. Biochem. J. 2004, 381 (1), 35–42. 10.1042/BJ20031933. 15070399 PMC1133759

[ref81] Rzem R.; Veiga-Da-Cunha M.; Noël G.; Goffette S.; Nassogne M. C.; Tabarki B.; Schöller C.; Marquardt T.; Vikkula M.; Van Schaftingen E. A Gene Encoding a Putative FAD-Dependent L-2-Hydroxyglutarate Dehydrogenase Is Mutated in L-2-Hydroxyglutaric Aciduria. Proc. Natl. Acad. Sci. U. S. A. 2004, 101 (48), 16849–16854. 10.1073/pnas.0404840101. 15548604 PMC534725

[ref82] Kranendijk M.; Struys E. A.; Salomons G. S.; Van Der Knaap M. S.; Jakobs C. Progress in Understanding 2-Hydroxyglutaric Acidurias. J. Inherit. Metab. Dis. 2012, 35 (4), 571–587. 10.1007/s10545-012-9462-5. 22391998 PMC3388262

[ref83] Tarhonskaya H.; Szöllössi A.; Leung I. K. H.; Bush J. T.; Henry L.; Chowdhury R.; Iqbal A.; Claridge T. D. W.; Schofield C. J.; Flashman E. Studies on Deacetoxycephalosporin C Synthase Support a Consensus Mechanism for 2-Oxoglutarate Dependent Oxygenases. Biochemistry 2014, 53 (15), 2483–2493. 10.1021/bi500086p. 24684493

[ref84] Fleitz F. J.; Lyle T. A.; Zheng N.; Armstrong J. D.; Volante R. P. Kilogram Scale Synthesis of the Pyrazinone Acetic Acid Core of an Orally Efficacious Thrombin Inhibitor. Synth. Commun. 2000, 30 (17), 3171–3180. 10.1080/00397910008086927.

[ref86] Huck J. H. J.; Struys E. A.; Verhoeven N. M.; Jakobs C.; Van Der Knaap M. S. Profiling of Pentose Phosphate Pathway Intermediates in Blood Spots by Tandem Mass Spectrometry: Application to Transaldolase Deficiency. Clin. Chem. 2003, 49 (8), 1375–1380. 10.1373/49.8.1375. 12881455

