# Supplementary material for: Focused Screening Identifies Different Sensitivities of Human TET Oxygenases to the Oncometabolite 2-Hydroxyglutarate
Source: J Med Chem. 2024 Jan 31;67(6):4525–40. doi: 10.1021/acs.jmedchem.3c01820 (PMC10983004; doi:10.1021/acs.jmedchem.3c01820)
Supplement: Supplementary file 1 — jm3c01820_si_001.pdf [file jm3c01820_si_001.pdf]

# Supporting Information

## Focused Screening Identifies Different Sensitivities of Human TET Oxygenases to the Oncometabolite 2-Hydroxyglutarate

Roman Belle[a,b], Hilal Saraç[a,b,c†], Eidarus Salah[a,d†], Bhaskar Bhushan[a,c†], Aleksandra Szykowska[d], Grace Roper[a,b], Anthony Tumber[a,f], Skirmantas Kriaucionis[e], Nicola Burgess-Brown[d], Christopher J. Schofield[a,f], Tom Brown[a], and Akane Kawamura[a,b,c*]

[a] Chemistry Research Laboratory, Department of Chemistry, University of Oxford, 12 Mansfield Road, OX1 3TA, Oxford, United Kingdom.
[b] Chemistry – School of Natural and Environmental Sciences, Bedson Building, Newcastle University, NE1 7RU, Newcastle upon Tyne, United Kingdom.
[c] Radcliffe Department of Medicine, Division of Cardiovascular Medicine, University of Oxford, Wellcome Trust Centre for Human Genetics, Roosevelt Drive, OX3 7BN, Oxford, United Kingdom.
[d] Centre for Medicines Discovery, University of Oxford, Old Road Campus Research Building, Roosevelt Drive, OX3 7DQ, Oxford, United Kingdom.
[e] Ludwig Institute for Cancer Research, Nuffield Department of Medicine, University of Oxford, Old Road Campus Research Building, Roosevelt Drive, OX3 7DQ, Oxford, United Kingdom.
[f] Ineos Oxford Institute for Antimicrobial Research, Department of Chemistry, University of Oxford, 12 Mansfield Road, OX1 3TA, Oxford, United Kingdom.

* Corresponding author; **Email**: akane.kawamura@newcastle.ac.uk.

† These authors contributed equally.

| **Table of Contents** | **Page** |
|---|---|

Figure S1. SDS-PAGE analysis of purified recombinant TET proteins used in this study. S4

Figure S2. AlphaScreen assay for measuring activity of TET enzymes. S5

Figure S3. Time-course of TET1$_{CD}$ (A), TET2$_{CD}$ (B), TET3$_{CD}$ (C), and TET2$_{CD\Delta LCI}$ (D) catalysed oxidation of $^{5m}$C 1 to $^{5hm}$C 2 measured by AlphaScreen. S6

Figure S4. Kinetic analysis of TET enzymes using the AlphaScreen assay. S7

Figure S5. $K_i$ determination of IOX1 3 (A), *S*-2HG 22 (B), *R*-2HG 23 (C) using the AlphaScreen assay displaying kinetic curves as AlphaScreen signal (counts) as function of the 2OG concentration (2–75 µM). S8

Figure S6. Kinetic analysis of TET2 using mass spectrometry assays. S9

Figure S7. Western blot analysis of stable U2OS cell lines overexpressing 3×FLAG-TET$_{CD}$1–3. S10

Figure S8. Representative immunofluorescence images of stable U2OS cells expressing Doxycycline (Dox)-inducible *N*-terminal 3×FLAG-tagged wild-type (WT) and mutant (mut) TET catalytic domains. S11

Figure S9. Quantitative analysis of global $^{5hm}$C levels of stably transfected TET$_{CD}$ expressing U2OS cells using high-content immunofluorescence imaging. S12

Figure S10. Structures of prodrugs used in cellular assays in this study. S13

Figure S11. High-content immunofluorescence imaging of stably transfected FLAG-TET1$_{CD}$ expressing U2OS cells dosed with small molecules. S14

Figure S12. Panobinostat 21 increases the global $^{5hm}$C levels in a concentration dependent manner. S15

Figure S13. High-content immunofluorescence imaging of stably transfected FLAG-TET$_{CD}$ expressing U2OS cells dosed with small molecules. S16

Table S1. Kinetic parameters for inhibition of recombinant TETs by IOX1 3 and 2HGs 22,23. S17

Table S2. Inhibition data for *R*-2HG and *S*-2HG with isolated 2OG dependent dioxygenases. S18

Table S3. Summary of cellular inhibition data for *R*-2HG and *S*-2HG with 2OG dependent dioxygenases. S19

Figure S14: $^{1}$H-NMR (400 MHz) spectrum of diethyloxalylglycine 38. S20

Figure S15: $^{13}$C-NMR (101 MHz) spectrum of diethyloxalylglycine 38. S20

Figure S16: $^{1}$H-NMR (400 MHz) spectrum of *N*-oxalylglycine 5; data are consistent with the literature. S21

Figure S17: $^{13}$C-NMR (126 MHz) spectrum of *N*-oxalylglycine 5. S21

Figure S18: HPLC trace of IOX1 3 using a UV-detector (254 nm). S22

Figure S19: HPLC trace of NOG 5 using a UV-detector (210 nm). S22

Figure S20: HPLC trace of IOX4 8 using a UV-detector (254 nm). S22

Figure S21: HPLC trace of FG-0041 12 using a UV-detector (254 nm). S23

Figure S22: HPLC trace of Vadadustat 13 using a UV-detector (254 nm). S23

Figure S23: HPLC trace of JIB-04 14 using a UV-detector (254 nm). S23

Figure S24: HPLC trace of Panobinostat 21 using a UV-detector (254 nm). S24

Figure S25: HPLC trace of disodium *S*-2-hydroxyglutarate 22 using a CAD-detector. S25

Figure S26: HPLC trace of disodium *R*-2-hydroxyglutarate 23 using a CAD-detector. S25

Figure S27: HPLC trace of (*S*)-Octyl-α-hydroxyglutaric acid 36 using a CAD-detector. S26

Figure S28: HPLC trace of (*R*)-Octyl-α-hydroxyglutaric acid 37 using a CAD-detector. S26

References S27-28

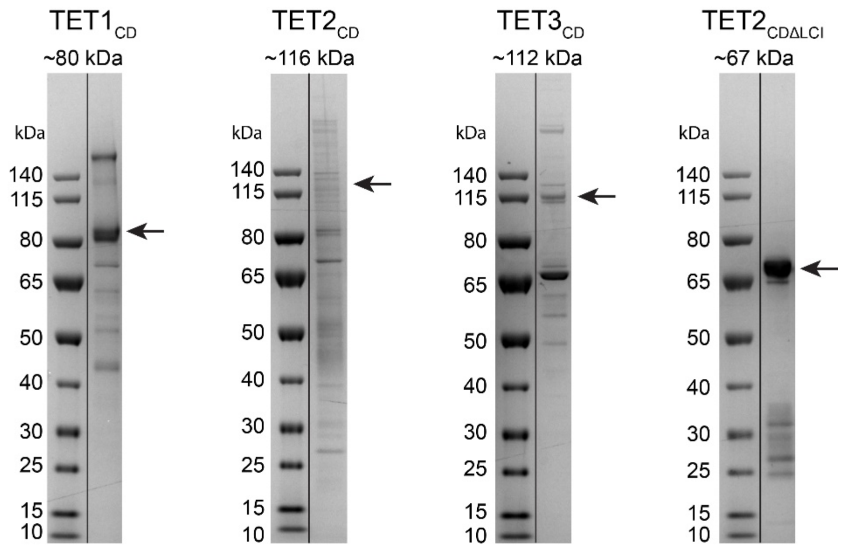


**Figure S1. SDS-PAGE analysis of partially purified recombinant TET proteins used in this study.** SDS-PAGE gels for purified 3×FLAG-TET1$_{CD}$ (~80 kDa), TET2$_{CD}$-His$_{10}$-FLAG (~116 kDa), His$_{10}$-TET3$_{CD}$ (~112 kDa), and His$_{6}$-TET2$_{CD\Delta LCI}$ (~67 kDa) are shown.

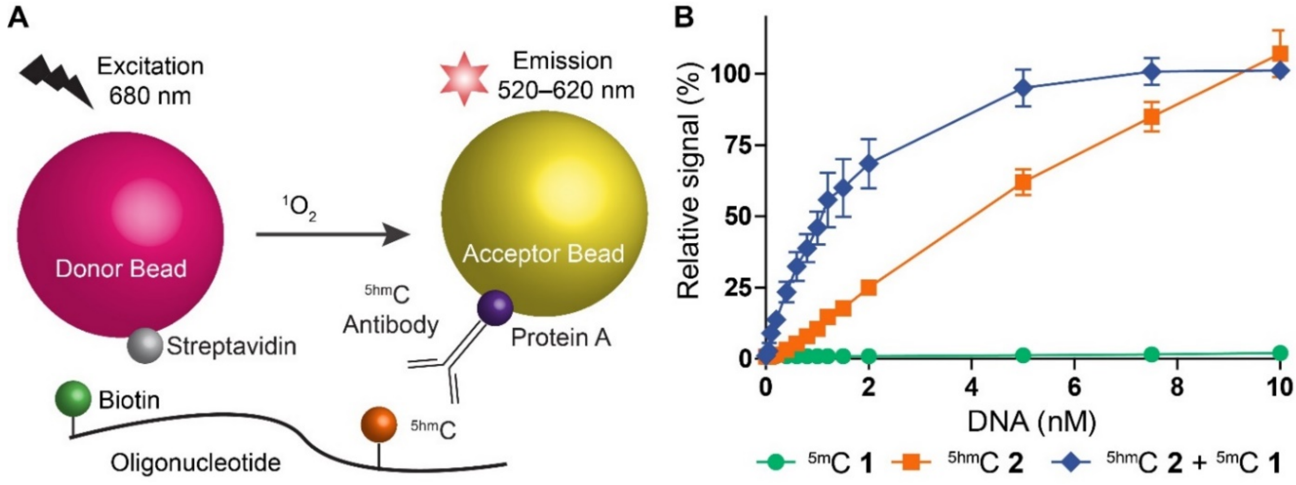


**Figure S2. AlphaScreen assay for measuring activity of TET enzymes.** (A) Schematic representation of the AlphaScreen assay. The streptavidin conjugated donor beads bind biotinylated DNA which, on TET catalysed oxidation of $^{5m}$C to $^{5hm}$C, can bind to a $^{5hm}$C selective antibody. The product is captured by Protein A conjugated acceptor beads. When the beads are excited (680 nm), the donor beads release singlet molecular oxygen that is converted to chemiluminescence by proximate acceptor beads (520–620 nm). (B) Standard curves for AlphaScreen for ssDNA containing $^{5m}$C **1** (green), $^{5hm}$C **2** (orange) or $^{5hm}$C **2** / $^{5m}$C **1** mixture (total concentration of DNA maintained to 10 nM, plotted as a function of $^{5hm}$C concentration) (blue). A standard curve for the $^{5hm}$C **2** / $^{5m}$C **1** mixture was used as an internal for calculating the activities of the TETs. Data are plotted as the mean ± StDev (n $\geq$ 7).

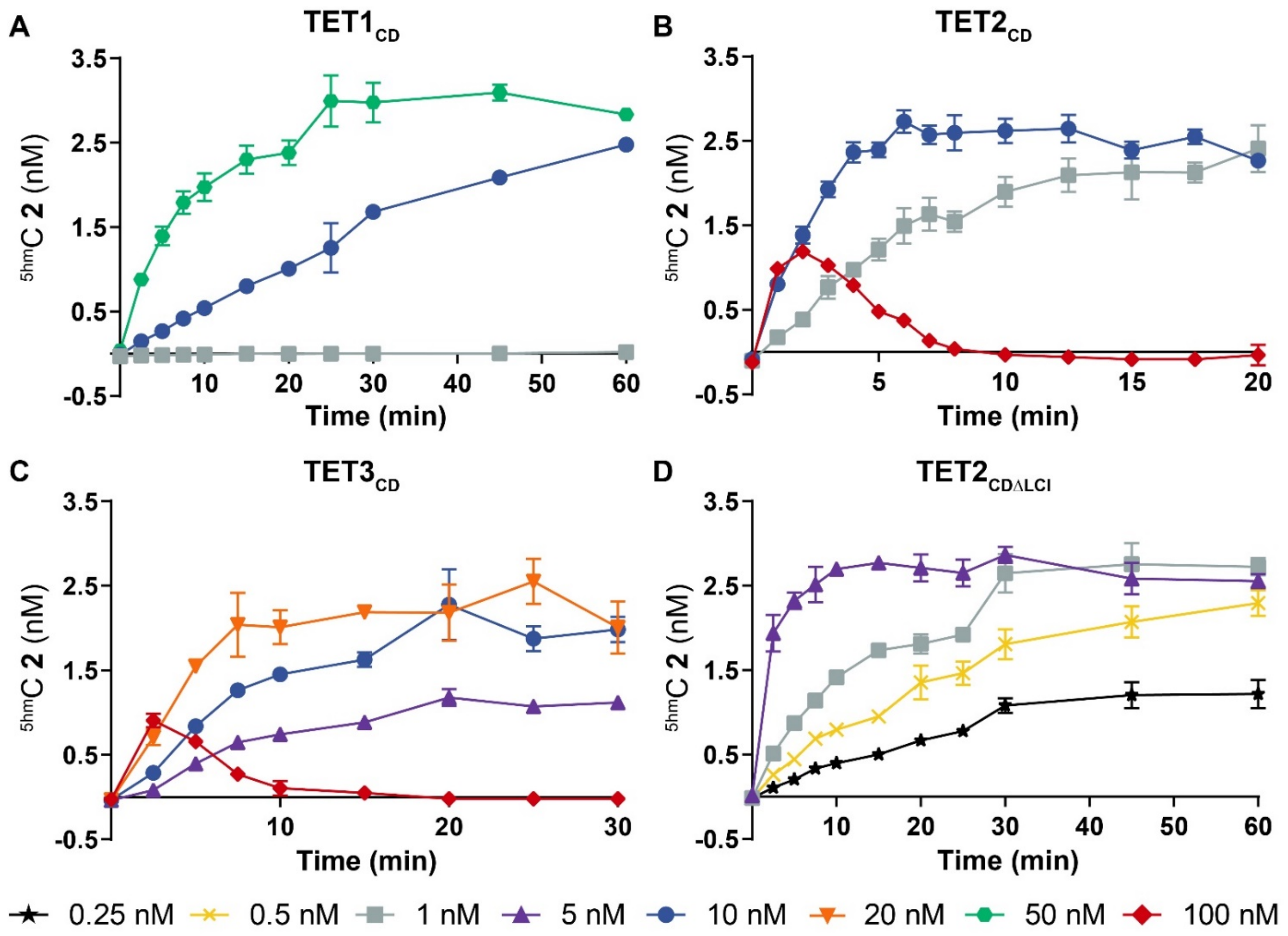


**Figure S3. Time-course of TET1$_{CD}$ (A), TET2$_{CD}$ (B), TET3$_{CD}$ (C), and TET2$_{CD\Delta LCI}$ (D) catalysed oxidation of $^{5m}$C 1 to $^{5hm}$C 2 measured by AlphaScreen.** Standard assay conditions: $^{5m}$C **1** (10 nM), Asc (100 µM), Fe(**II**) (10 µM), and 2OG (10 µM), TET enzyme (0.25–100 nM) at r.t. Data are plotted as the mean ± StDev (n $\geq$ 3). The turnover to $^{5hm}$C **2** product was calculated using an internal standard curve (Figure S2B) carried out on each independent assay plate.

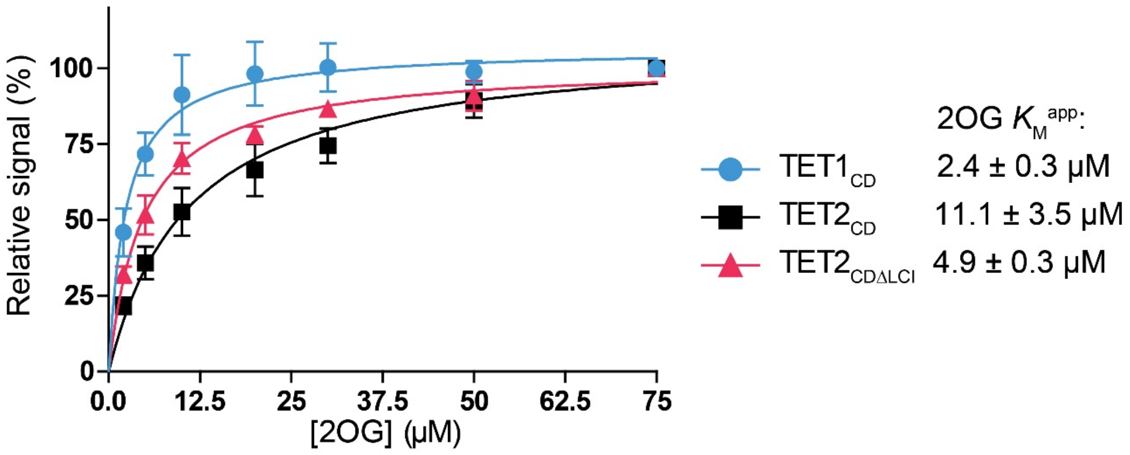


**Figure S4. Kinetic analysis of TET enzymes using the AlphaScreen assay.** $K_M^{app}$ of 2OG of $TET1_{CD}$ (10 nM), $TET2_{CD}$ (1 nM) and $TET2_{CD\Delta LCI}$ (1 nM) were determined using the AlphaScreen assay, measuring the AlphaScreen signal at each 2OG concentration with 10 nM $^{5m}C$ **1** substrate. The signal was normalized against the activity observed at 75 μM 2OG, where maximal activity was observed. Standard conditions: $^{5m}C$ **1** (10 nM), Asc (100 μM), Fe(**II**) (10 μM), 2OG (2–75 μM). Data plotted as the mean ± StDev (n= 5).

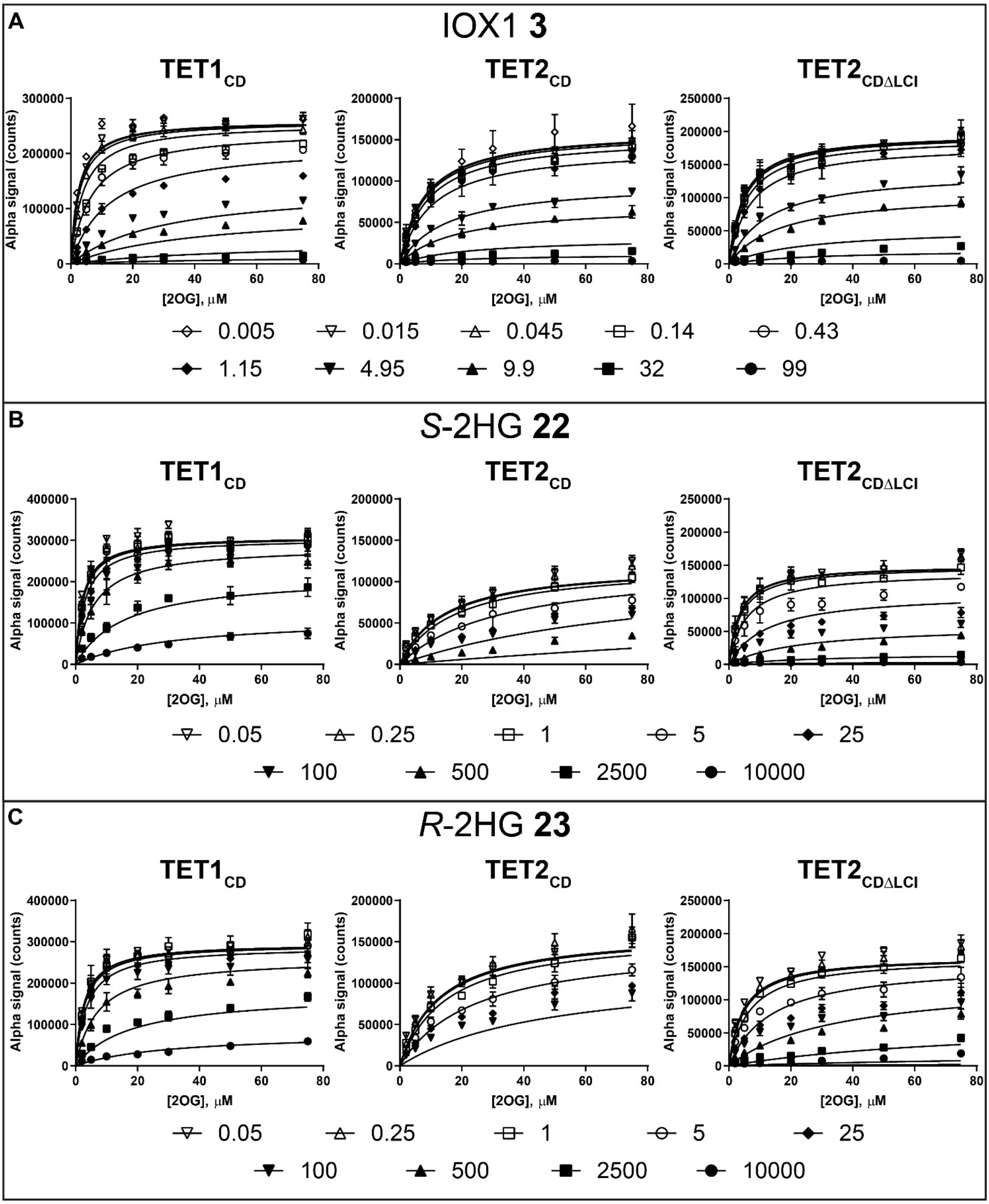


**Figure S5. $K_i$ determination for IOX1 3 (A), *S*-2HG 22 (B), *R*-2HG 23 (C) using the AlphaScreen assay displaying kinetic curves as the AlphaScreen signal (counts) as function of the 2OG concentration (2–75 μM)**. Standard conditions: $^{5m}$C **1** (10 nM), Asc (100 μM), Fe(II) (10 μM), 2OG (2–75 μM). Data plotted as the mean ± StDev (n = 2–4). The concentrations of inhibitor (μM) is given under each plot. See Table S1 for $K_i$ values.

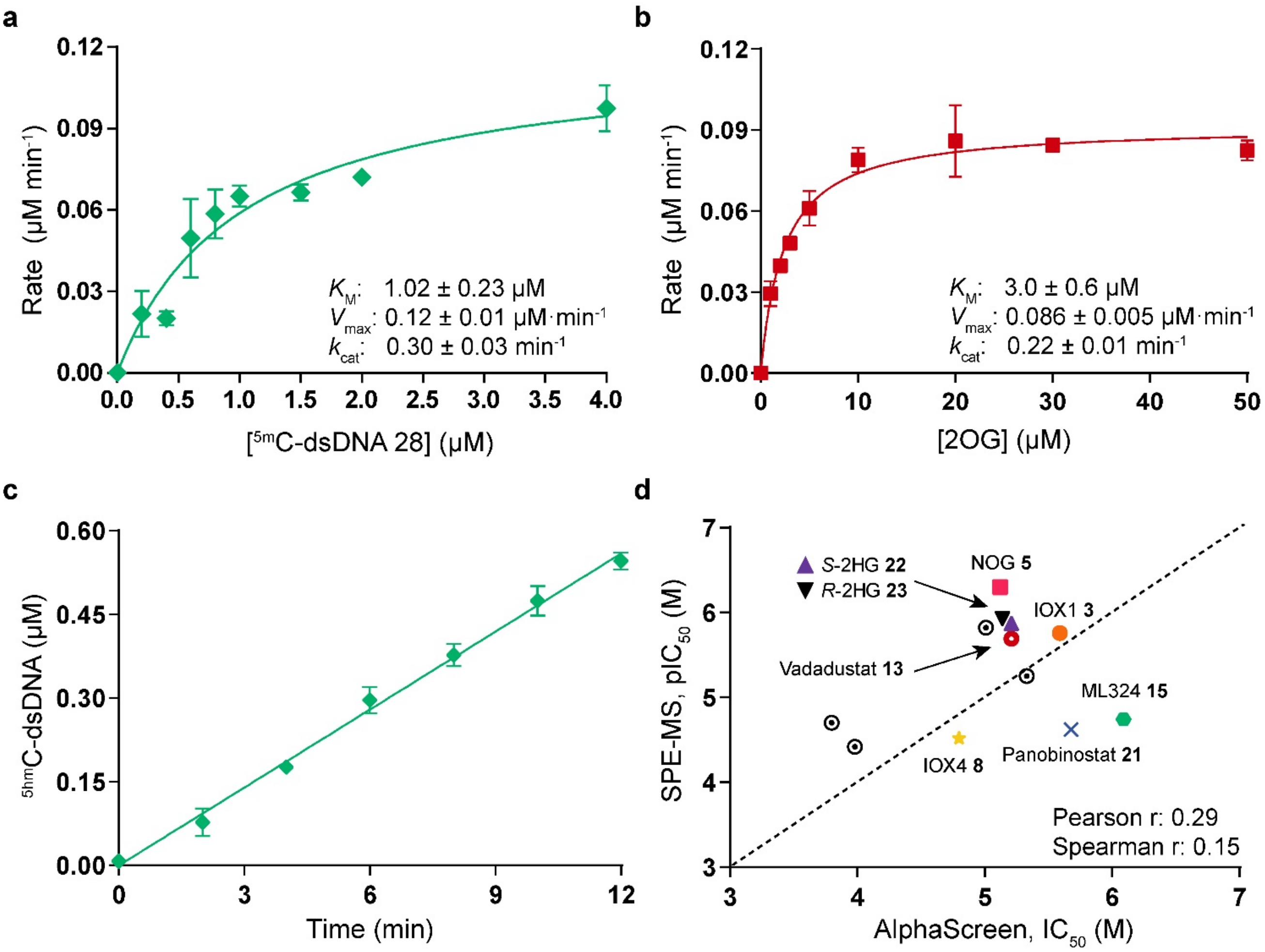


**Figure S6. Kinetic analysis of TET2 using mass spectrometry assays.** (A) Michaelis-Menten kinetics analysis of TET2$_{CD}$ with variable $^{5m}$C-DNA **28** substrate concentrations determined using MALDI-TOF MS. Standard conditions: $^{5m}$C-DNA **28** (0.2–4.0 μM), TET2$_{CD}$ (0.4 μM), Asc (500 μM), Fe(II) (50 μM) and 2OG (100 μM). Values shown as the mean ± StDev. from two independent kinetic curves. (B) Representative Michaelis-Menten analysis of TET2$_{CD\Delta LCI}$ with varied 2OG concentrations determined using SPE-MS. Standard conditions: $^{5m}$C-DNA **28** (2.0 μM), TET2$_{CD\Delta LCI}$ (0.4 μM), Asc (200 μM), Fe(II) (50 μM), and 2OG. Data plotted as the mean ± StDev (n = 3). from kinetic analysis of 4 independent curves: $K_M$: 3.0 ± 0.6 μM; $V_{max:}$ 0.086 ± 0.005 μM·min$^{-1}$; $k_{cat}$: 0.22 ± 0.01 min$^{-1}$. (C) Time course of $^{5m}$C-DNA **28** oxidation by TET2$_{CD\Delta LCI}$ (0.4 μM) acquired using SPE-MS and represented as $^{5hm}$C (μM) produced in the assay. Linear range (up to 12 min) is shown ($R^2$: 0.99). Standard conditions: $^{5m}$C-DNA **28** (2.0 μM), TET2$_{CD\Delta LCI}$ (0.4 μM), Asc (200 μM), Fe(II) (50 μM) and 2OG (10 μM). Data plotted as the mean ± StDev (n = 4). (D) Correlation plot of pIC$_{50}$ values for TET2$_{CD\Delta LCI}$ as determined by AlphaScreen and SPE-MS assays. Circled dots are other compounds from Table 1.

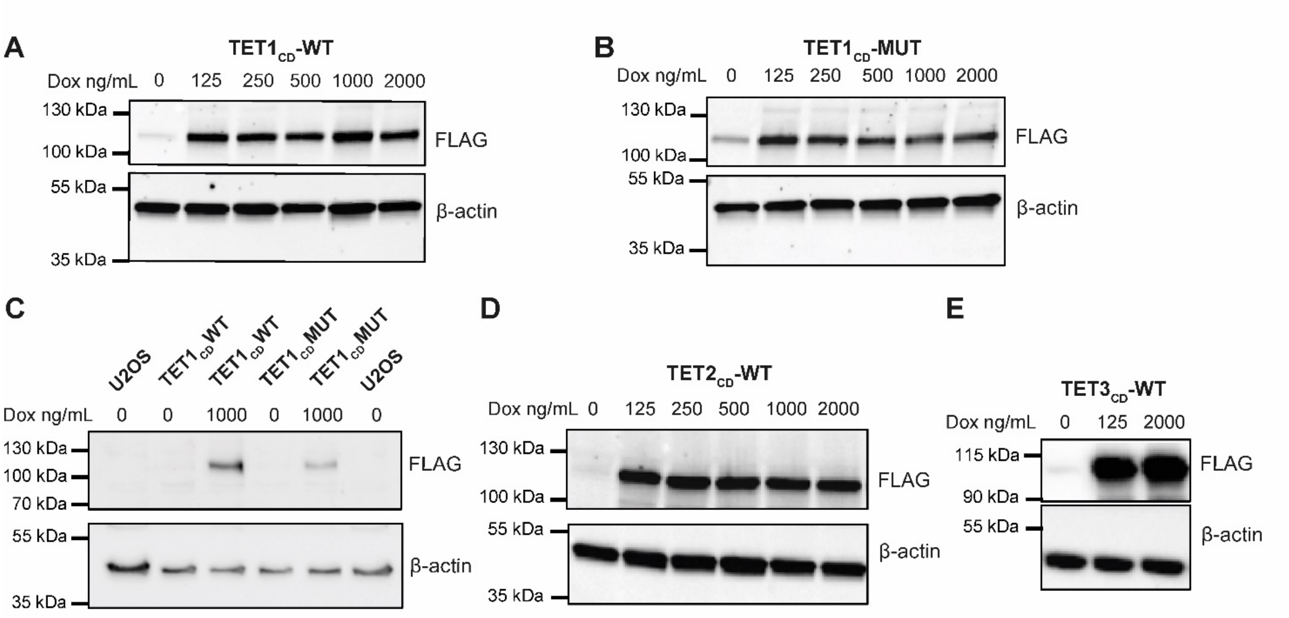


**Figure S7. Western blot analysis of stable U2OS cell lines overexpressing 3×FLAG-TET$_{CD}$1–3.**
Dox titration (24 h) of U2OS cells stably transfected with 3×FLAG-TET1–3$_{CD}$ were analysed with anti-FLAG and anti-ß-actin antibodies. (A)TET1$_{CD}$-WT; (B) TET1$_{CD}$-Mut; (C) Western blot of Dox induced (1000 ng / mL) or uninduced TET1$_{CD}$WT and TET1$_{CD}$MUT U2OS cells and standard U2OS cells. See Methods and Materials for details; (D) TET2$_{CD}$-WT and (E) TET3$_{CD}$-WT.

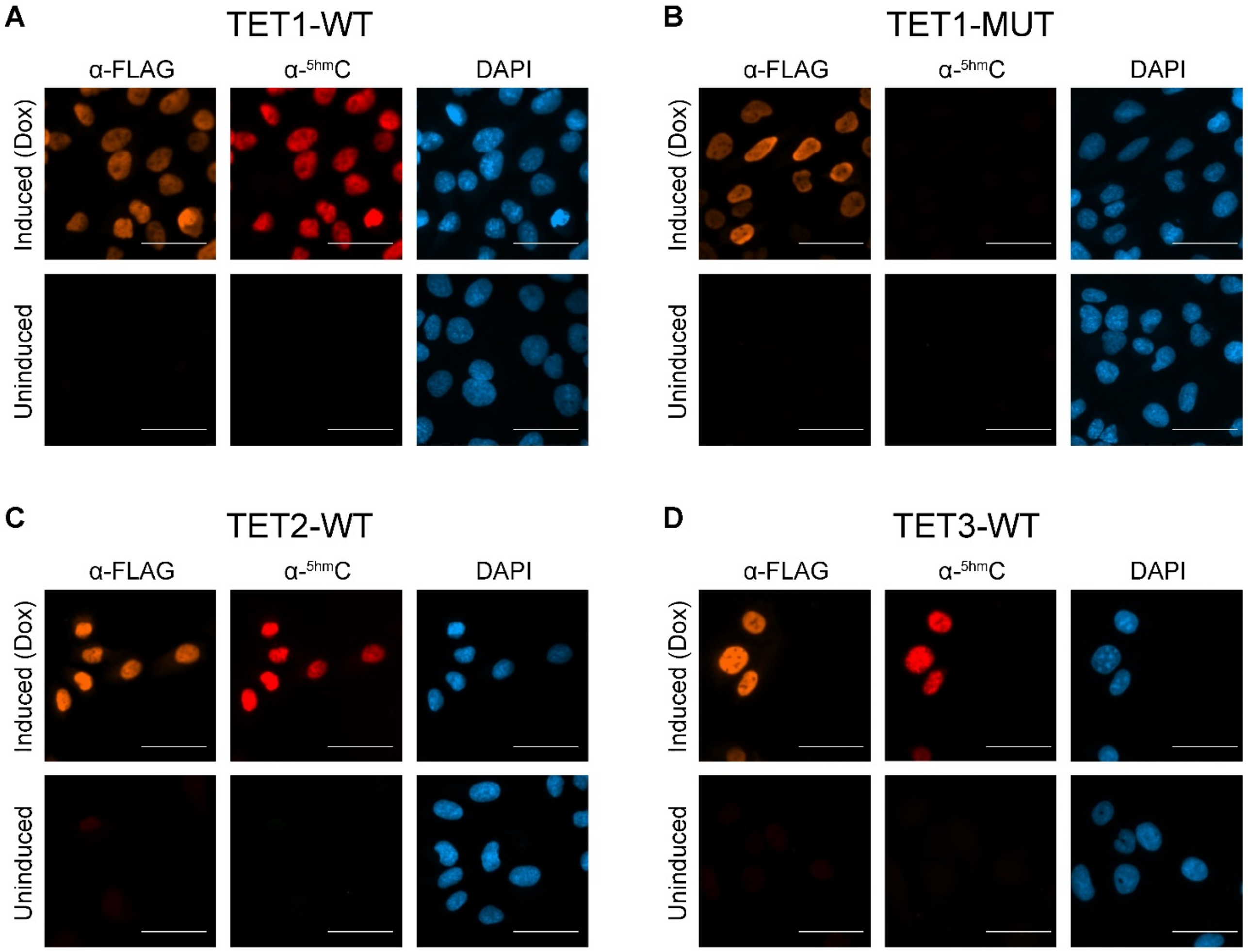


**Figure S8. Representative immunofluorescence images of stable U2OS cells expressing Dox-inducible *N*-terminal 3×FLAG-tagged wild-type (WT) and mutant (MUT) TET catalytic domains.**
Cells were treated with or without Dox (1 μg/mL) for 24 h, fixed and stained with DAPI (nuclear), anti-FLAG (3×FLAG-TET), and anti-$^{5hm}$C antibodies: (A) TET1$_{CD}$-WT, (B) mutant TET1$_{CD}$-MUT (C) wild-type TET2$_{CD}$, and (D) wild-type TET3$_{CD}$. Scale bar: 50 μm. Images acquired using Cell Discoverer 7 high-throughput (Zeiss) systems.

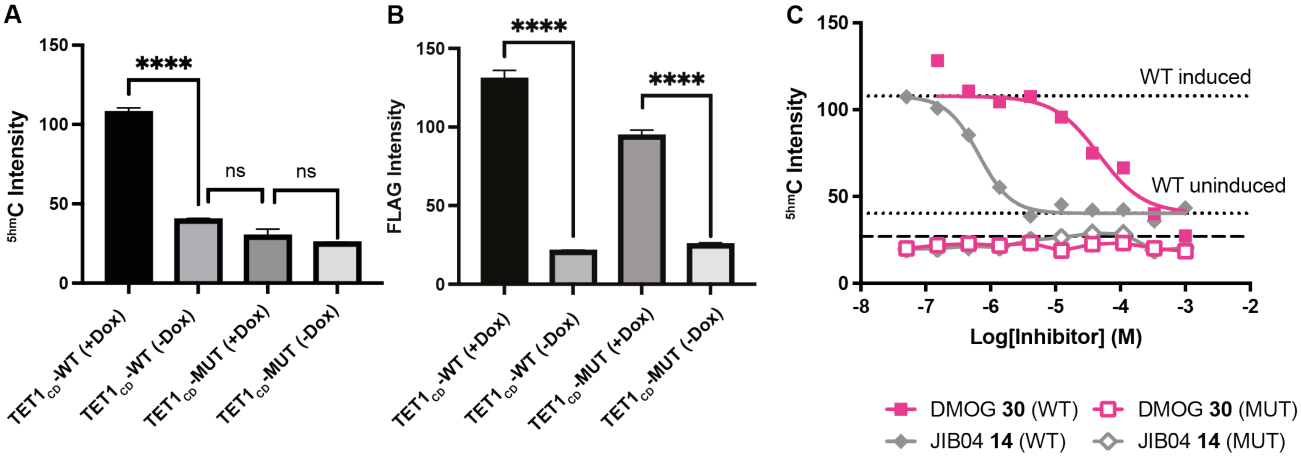


**Figure S9. Quantitative analysis of global $^{5hm}$C levels of stably transfected TET1$_{CD}$ expressing U2OS cells using high-content immunofluorescence imaging.** FLAG-TET1$_{CD}$ wild-type (WT) or catalytically inactive mutant (MUT) stable U2OS cells were treated with or without Dox for 24 h, fixed and stained with DAPI (nuclear), anti-FLAG and anti-$^{5hm}$C antibodies. The global $^{5hm}$C levels were measured as the mean ± s.e.m (n > 3000 cells). (A) An increase in $^{5hm}$C levels correlates with TET1$_{CD}$ wild-type expression, but not with mutant. (B) An increase in cellular FLAG staining correlating with the expression of FLAG-TET1$_{CD}$ (WT) and FLAG-TET1$_{CD}$ (MUT) were observed when the cells were treated with Dox. Statistical analysis was carried out using the unpaired t-test with Welsch's correction (**** = P $\leq$ 0.001, ns = P $\geq$ 0.05). (C) Exemplar dose-response curves of compound treatment generated using the immunofluorescence assay. Dox-inducible wild-type or mutant TET1$_{CD}$ expressing stable U2OS cells with or without Dox were treated with DMSO, JIB-04 **14** or DMOG **30** (1% (v/v) DMSO final conc.) for 24 h. The $^{5hm}$C levels of 1% (v/v) DMSO treated controls of TET1$_{CD}$-WT (dotted-line, black) and TET1$_{CD}$-MUT (dashed-line, grey) with and without Dox are indicated as additional grid lines on the y-axis. Note 5hmC levels for MUT induced and uninduced cells are the same. Data are plotted as mean ± s.e.m (n > 3000 cells). TET1$_{CD}$-WT data were fitted using non-linear regression (four parameters) with top and bottom constraints as Dox-induced and uninduced DMSO treated WT cells, respectively. In some cases, the WT uninduced cells had slightly higher $^{5hm}$C levels than induced mutant expressing cells, in part due to basal level of leaky TET1 expression (Figure S7). FLAG-TET1$_{CD}$-WT and FLAG-TET1$_{CD}$-MUT U2OS cells were treated at the same time, on different 96-well plates. Images acquired using Operetta CLS High-Content Analysis (PerkinElmer).

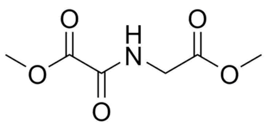

dimethyl-NOG
DMOG, **30**
(prodrug of **5**)

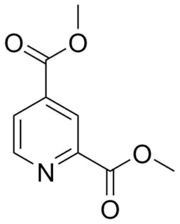

dimethyl-2,4-PDCA
**31**
(prodrug of **4**)

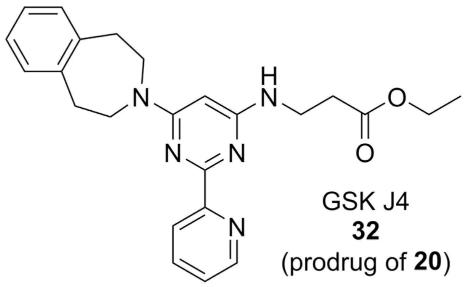

GSK J4
**32**
(prodrug of **20**)

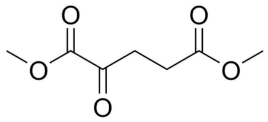

dimethyl-2OG
**33**
(prodrug of 2OG)

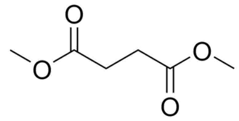

dimethyl succinate
**34**
(prodrug of **24**)

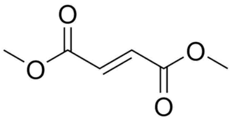

dimethyl fumarate
**35**
(prodrug of **25**)

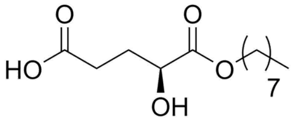

octyl-(*S*)-2HG
**36**
(prodrug of **22**)

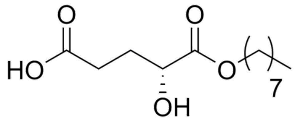

octyl-(*R*)-2HG
**37**
(prodrug of **23**)

**Figure S10. Structures of prodrugs used in cellular assays in this study.**

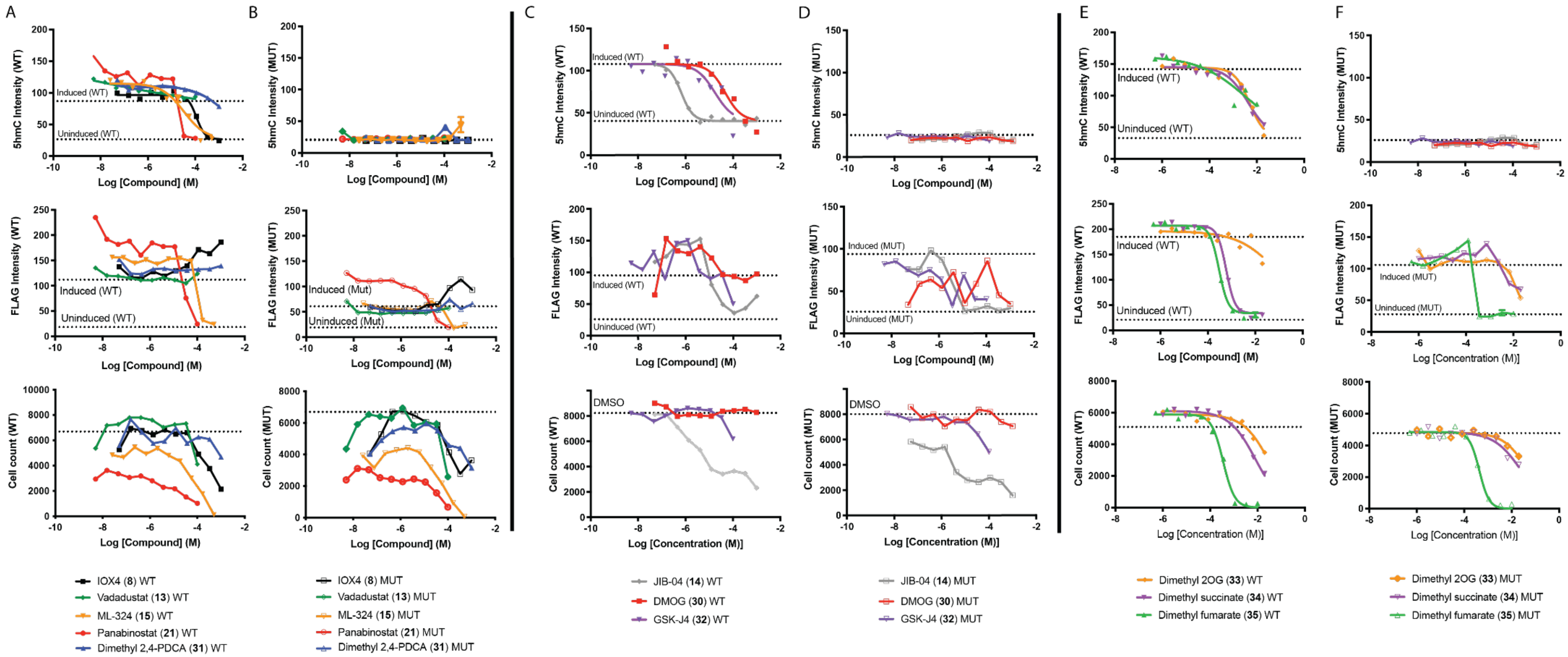


**Figure S11. High-content immunofluorescence imaging of stably transfected FLAG-TET1$_{CD}$ expressing U2OS cells dosed with small molecules.** Doxycycline (Dox)-inducible FLAG-TET1$_{CD}$ wild-type (WT) or catalytically inactive mutant (MUT) stable U2OS cells were treated with Dox and small molecules (in DMSO) for 24 h. Cells were fixed and stained with DAPI (nuclear), anti-FLAG and anti-$^{5hm}$C antibodies. The global $^{5hm}$C levels (top row) and FLAG-levels (middle row) of cells were measured as a mean fluorescence intensity ± s.e.m. Fluorescence intensities of induced control (+Dox, DMSO treatment) and uninduced control (-Dox, DMSO treatment) are indicated as grid lines on the y-axis. Exemplar dose-response curves of compound treatment are provided. Dose-response data were fitted using non-linear regression (four parameters) with top and bottom constraints as Dox-induced and uninduced DMSO treated WT cells, respectively. Bottom row shows the total cell count (DAPI nuclear staining) of the images. (A, C, E) TET1$_{CD}$ wild-type cells treated with compounds; (B, D, F) TET1$_{CD}$ mutant cells treated with compounds. Images acquired using Operetta CLS High-Content Analysis (PerkinElmer).

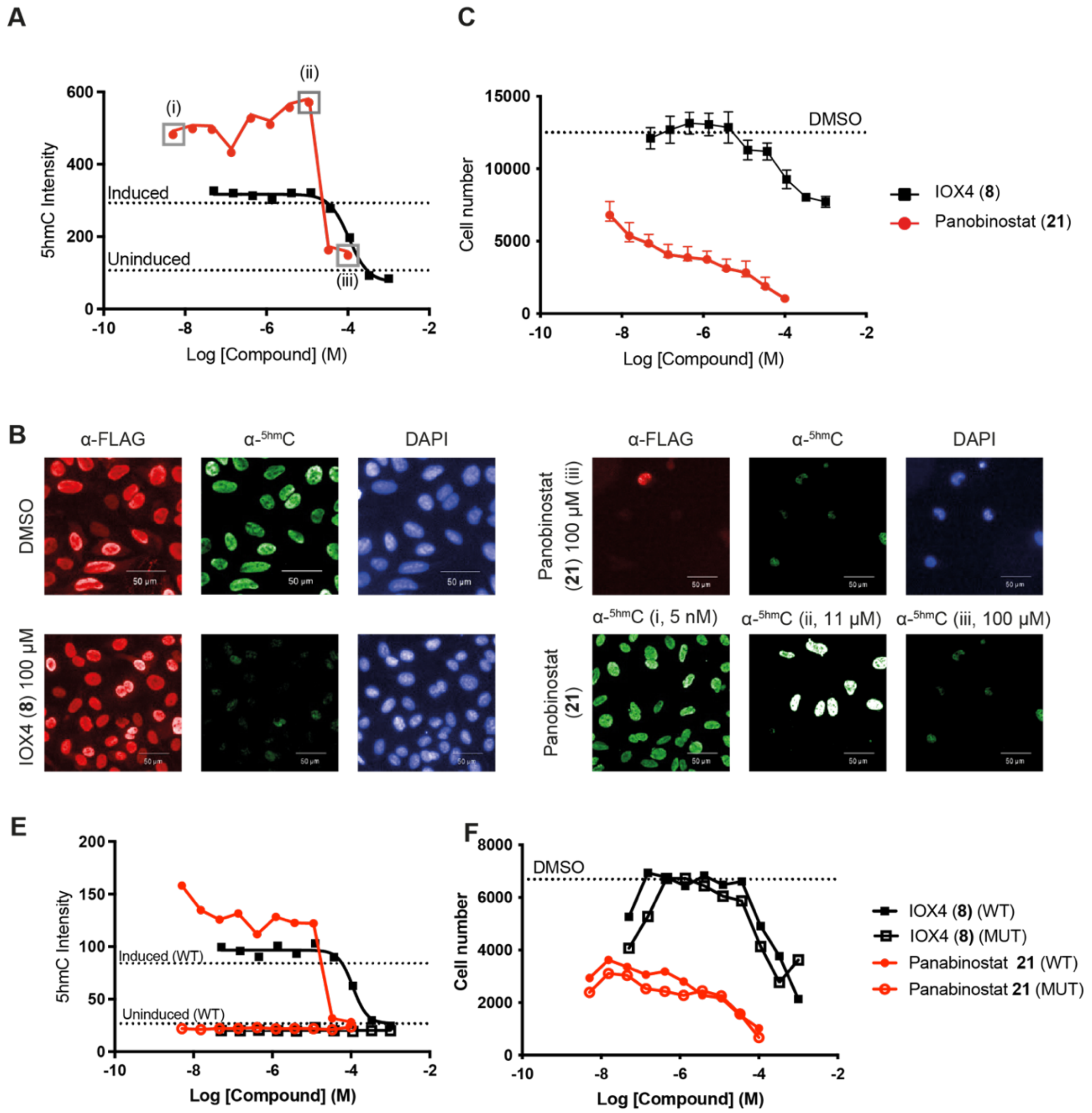


**Figure S12. Panobinostat 21 increases the global 5hmC levels in a concentration dependent manner.** (**A**) Representative dose-response curves of cellular immunofluorescence assay with Panobinostat **21** and IOX4 **8** treatment. Doxycycline (Dox)-inducible FLAG-TET1CD-WT expressing stable U2OS cells were treated with or without Dox and dosed with DMSO, IOX4 **8**, or Panobinostat **21** (1% (v/v) DMSO final concentration) for 24 h, fixed and stained with DAPI (nuclear), anti-FLAG and anti-5hmC antibodies (see Supplementary Methods). The 5hmC levels of 1% (v/v) DMSO treated controls (TET1CD-WT with and without Dox) are indicated as additional dashed grid lines on the y-axis (induced/uninduced). Data are plotted as a mean ± s.e.m (n > 3000 cells, except for >10 μM for Panobinostat **21** where n >1000 cells used). Scale bar: 50 μm. (**B**) Representative immunofluorescence images of select data points of Dox-induced TET1CD-WT expressing stable U2OS cells. Images of cells treated with DMSO or compounds from (**A**) are shown, including 5hmC staining at different Panobinostat **21** concentrations (i) 5 nM, (ii) 11 μM, (iii) 100 μM. Scale bar: 50 μm. (**C**) Cell counts from immunofluorescence images (nuclear counts, 9 fields (×10 magnification)) plotted against inhibitor concentrations. Mean, error given as ± s.e.m (3 independent biological experiments). (**E, F**) An exemplar dose-response curves with Dox-inducible FLAG-TET1CD-WT (closed symbols) or FLAG-TET1CD-MUT (open symbols) expressing stable U2OS cells treated with or without Dox and dosed with compounds as described in (A). No changes in 5hmC levels (**E**) were observed for FLAG-TET1CD-MUT expressing cells dosed with IOX4 **8** or Panabinostat **21**. (**F**) No differences in the cell numbers were observed between the FLAG-TET1CD-WT or FLAG-TET1CD-MUT overexpressing cells. Images acquired using Operetta CLS High-Content Analysis (PerkinElmer).

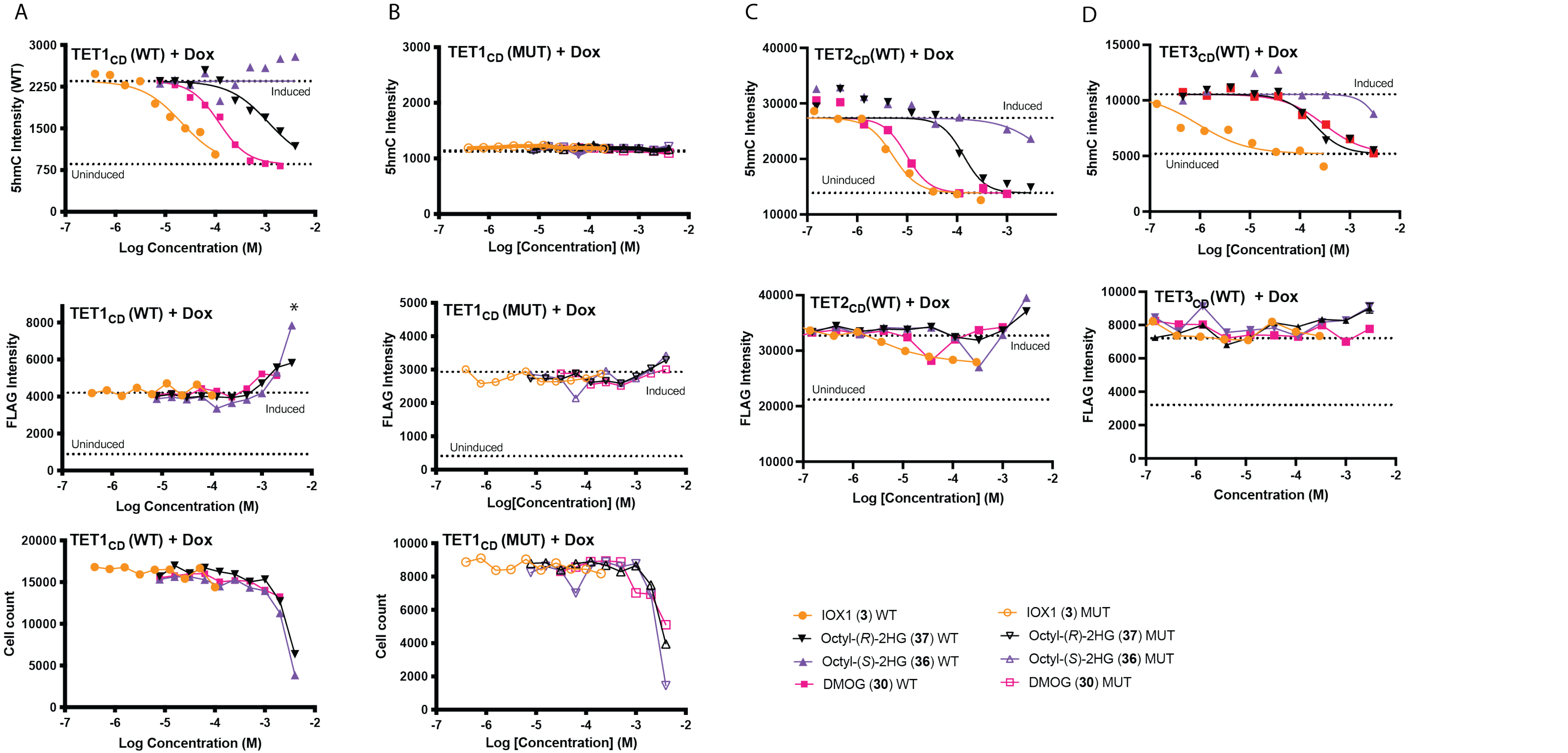


**Figure S13. High-content immunofluorescence imaging of stably transfected FLAG-TET$_{CD}$ expressing U2OS cells dosed with small molecules.** Doxycycline (Dox)-inducible FLAG-TET1$_{CD}$, TET2$_{CD}$ or TET3$_{CD}$ wild-type (WT), or catalytically inactive TET1$_{CD}$ mutant (MUT) stable U2OS cells were treated with Dox and small molecules (in DMSO) for 24 h. Cells were fixed and stained with DAPI (nuclear), anti-FLAG and anti-$^{5hm}$C antibodies. The global $^{5hm}$C levels (top row) and FLAG-levels (middle row) of cells were measured as a mean fluorescence intensity (n > 1000 except *) ± s.e.m. Fluorescence intensities of induced control (+Dox, DMSO treatment) and uninduced control (-Dox, DMSO treatment) are indicated as grid lines on the y-axis. Exemplar dose-response curves of compound treatment are provided. Dose-response data were fitted using non-linear regression (four parameters) with top and bottom constraints as Dox-induced and uninduced DMSO treated WT cells, respectively. Bottom row shows the total cell count (DAPI nuclear staining) of the images. (A) TET1$_{CD}$ wild-type, (B) TET1$_{CD}$ mutant, (C) TET2$_{CD}$ wild-type and (D) TET3$_{CD}$ wild-type cells treated with compounds. Images acquired using Cell Discoverer 7 high-throughput (Zeiss) systems.

**Table S1. Kinetic parameters for inhibition of recombinant TETs by IOX1 3 and 2HGs 22,23.** $K_i$ values for IOX1 **3**, *S*-2HG **22**, and *R*-2HG **23** with respect to 2OG (2–75 μM) competition as determined using the AlphaScreen assay (Figure S5). $K_i$ values in μM. Mean ± StDev (n= 4 independent experiments).

| | | TET1$_{CD}$ | | TET2$_{CD}$ | | TET2$_{CD\Delta LCI}$ | |
|---|---|---|---|---|---|---|---|
| Compound | | $K_i$ | α-value | $K_i$ | α-value | $K_i$ | α-value |
| IOX1 | **3** | 0.213 ± 0.030 | 28 ± 9 | 2.31 ± 0.59 | 3.3 ± 1.4 | 1.77 ± 0.36 | 6.7 ± 2.5 |
| *S*-2HG | **22** | 269 ± 59 | 21 ± 10 | 18.8 ± 9.5 | $> 10^6$ | 6.08 ± 1.69 | 10 ± 5 |
| *R*-2HG | **23** | 246 ± 46 | 15 ± 6 | 22.6 ± 7.2 | 11 ± 13 | 11.5 ± 2.9 | 31 ± 30 |

**Table S2. Inhibition data for *R*-2HG and *S*-2HG with isolated 2OG dependent dioxygenases.** Reported inhibition data are referenced. $K_i$ values are experimentally determined, except for those labelled*, where the $K_i$ values were calculated using the $IC_{50}$ values in columns 2 and 3 of the Table, assuming competitive binding using the Cheng-Prussoff equation $K_i = IC_{50} / (1+ [S]/K_M)$. N. S. – Not specified. Experimental screening methods a: AlphaScreen; b: MALDI-TOF; c: SPE-MS; d: $^{14}CO_2$ Radioactive; e: FDH; f: NMR; g: hydroxyl-[$^{14}$C]-proline radioactive. N.A.: Not applicable; N.S.: not specified; *Calculated $K_i$.

| Enzyme | $K_M$ (2OG), μM | $IC_{50}$, μM | | [2OG] | [Enzyme] | $K_i$, μM | |
|---|---|---|---|---|---|---|---|
| | | *R*-2HG | *S*-2HG | μM | nM | *R*-2HG | *S*-2HG |
| TET1CD | 2.4 [a] | 676 [a] | 1047 [a] | 10 | 10 | 246 [a] | 269 [a] |
| TET2CD | 11.1 [a] | 15 [a] | 13 [a] | 10 | 1 | 34 [a] | 19 [a] |
| | 1.02 [b] | - | - | - | - | - | - |
| TET2CDΔLCI | 4.90 [a] | 7 [a] | 6 [a] | 10 | 1 | 12 [a] | 6 [a] |
| | 3.02 [c] | 1.2 [c] | 1.3 [c] | 10 | 400 | - | - |
| | 15.7 [b, 1] | 5,300 [b, 1] | 12,400 [b, 1] | 100 | 5,000 | 885* | 2,071* |
| TET3CD | 24.2 [b, 1] | 95 [a] | 105 [a] | 10 | 10 | 239* | 265* |
| mTet1 | 55 [d, 2] | 4,000 [d, 2] | 1,000 [d, 2] | 240 | N.S. | 933* | 233* |
| mTet2 | 60 [d, 2] | 5,000 [d, 2] | 1,600 [d, 2] | 240 | N.S. | 1271* | 407* |
| FIH | 110 [b, 3] | 1,500 [b, 4] | 189 [b, 4] | 100 | 1,000 | 1,665* | 210* |
| PHD1 | 60 [d, 5] | - | - | - | - | 1,150 [d, 6] | - |
| PHD2 | 13 [b, 3] | 7,300 [b, 4] | 419 [b, 4] | 60 | 500 | 1,703* | 98* |
| | 60 [d,] | - | - | - | - | 625 [d, 6] | - |
| PHD3 | 55 [d, 5] | - | - | - | - | 90 [d, 6] | - |
| ABH2 | 4 [e, 4] | 424 [e, 4] | 150 [e, 4] | 10 | 2,000 | 212* | 75* |
| BBOX1 | 160 [f, 7] | 13,200 [f, 4] | 142 [f, 4] | 40 | 25 | 53,130* | 572* |
| KDM2A | 6 [e, 4] | 106 [e, 4] | 48 [e, 4] | 10 | 2,000 | 74* | 34* |
| KDM4A | 6 [e, 4] | 24 [e, 4] | 26 [e, 4] | 10 | 2,000 | 13 [e, 4] | 30 [e, 4] |
| | 15 [d, 8] | 160 [d, 8] | 290 [d, 8] | 50 | N.S. | 51* | 93* |
| KDM4B | 6 [d, 8] | 150 [d, 8] | 450 [d, 8] | 20 | N.S. | 53* | 158* |
| KDM4C | 4 [e, 4] | 79 [e, 4] | 97 [e, 4] | 10 | 1000 | 40* | 49* |
| KDM5B | 10 [d, 8] | 3,600 [d, 8] | 1,600 [d, 8] | 40 | N.S. | - | - |
| | - | - | - | N.A. | N.S. | 10,870 [b, 9] | 628 [b, 9] |
| | 2.6 [b, 10] | 203 [b, 10] | 150 [b, 10] | 3 | 600 | 244* | 180* |
| KDM6A | 8 [d, 8] | 180 [d, 8] | 180 [d, 8] | 37.5 | N.S. | 43* | 43* |
| KDM6B | 50 [d, 8] | 350 [d, 8] | 750 [d, 8] | 200 | N.S. | 89* | 191* |

**Table S3. Summary of cellular inhibition data for *R*-2HG and *S*-2HG with 2OG dependent dioxygenases.** Reported inhibition data is referenced. Experimental screening method on cell line with: a: by immunofluorescence; b: western blot; or c: by dot blot and LC-MS/MS.

| | *R*-2HG (μM) | *S*-2HG (μM) | Cell line |
|---|---|---|---|
| $TET1_{CD}$ | 1480 [a] | >1000 [a] | U2OS-$TET1_{CD}$ |
| $TET2_{CD}$ | 132 [a] | >3160 [a] | U2OS-$TET2_{CD}$ |
| $TET3_{CD}$ | 186 [a] | >3160 [a] | U2OS-$TET3_{CD}$ |
| PHD1<br>PHD2<br>PHD3 | Putative inhibition of PHD resulting in HIF1α stabilization inducing cellular hypoxic response. [b, 4] | - | RCC4/VHLHA, Hep3B, MCF7 |
| ABH2 | Inhibition [b, 11] | - | U87-MG, U373-MG, HT-1080 |
| ABH3 | Inhibition [b, 11] | - | U87-MG, U373-MG, HT-1080 |
| KDM4A | Inhibition [a, 4] | Inhibition [a, 4] | HeLa |
| KDM5B | No inhibition (2.0 mM) [a, 10] | No inhibition (1.0 mM) [a, 10] | U2OS |
| FTO | Inhibition [c, 12] | - | NOMO-1, U937, MA9.3ITD |

# NMR spectra

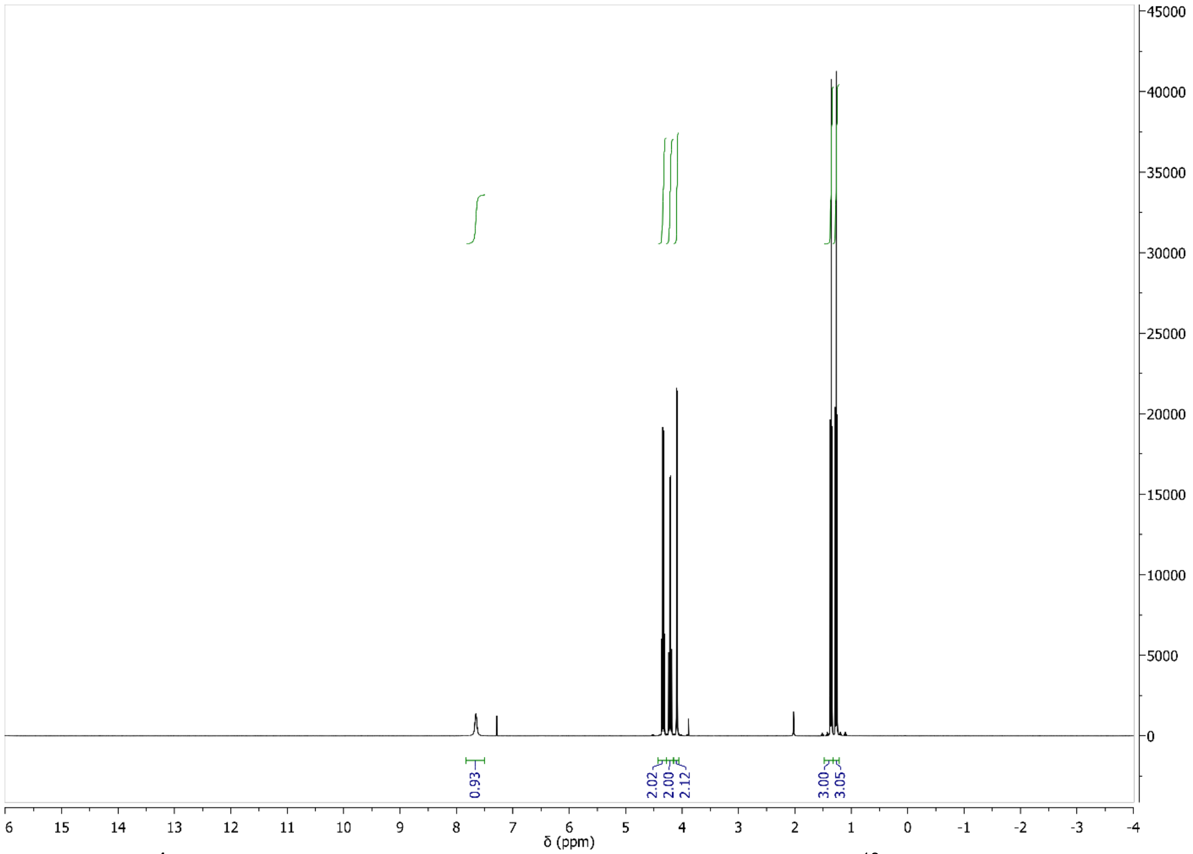


**Figure S14: $^{1}$H-NMR (400 MHz) spectrum of diethyloxalylglycine 38.** [13]

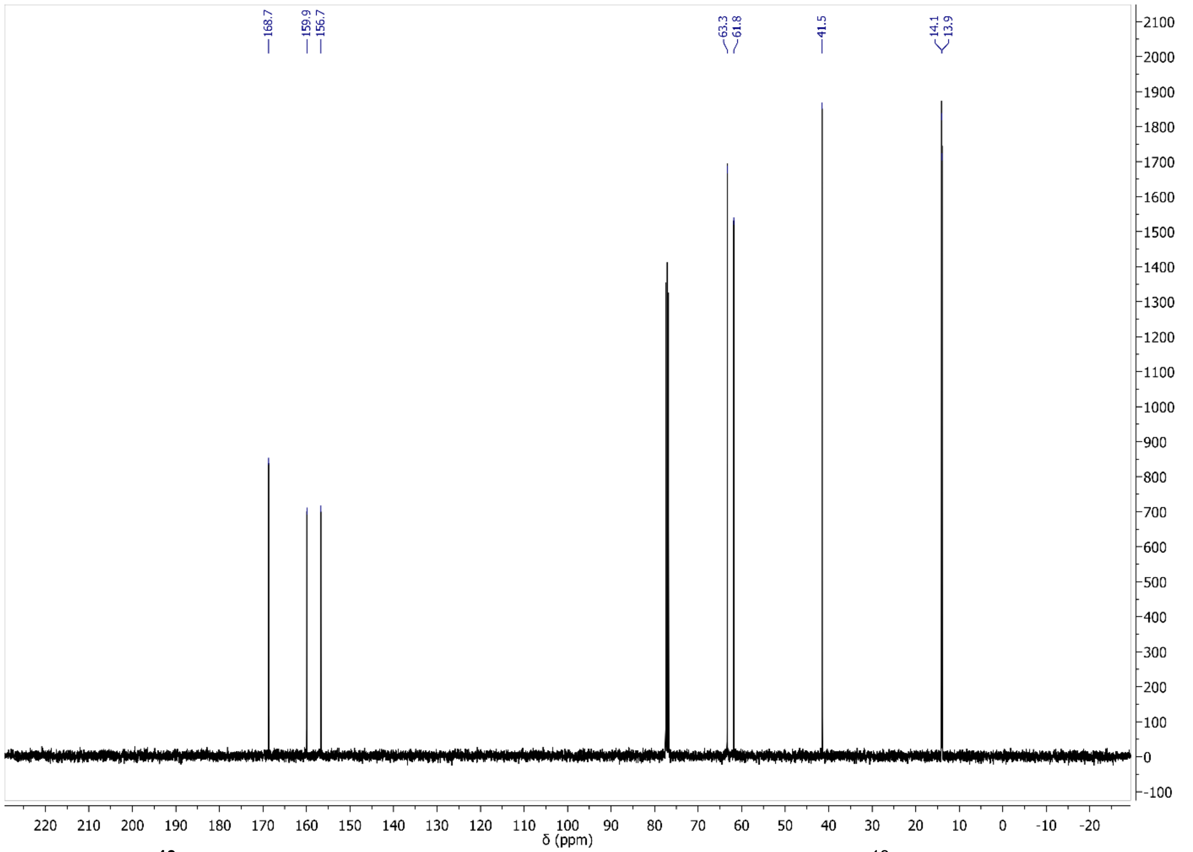


**Figure S15: $^{13}$C-NMR (101 MHz) spectrum of diethyloxalylglycine 38.** [13]

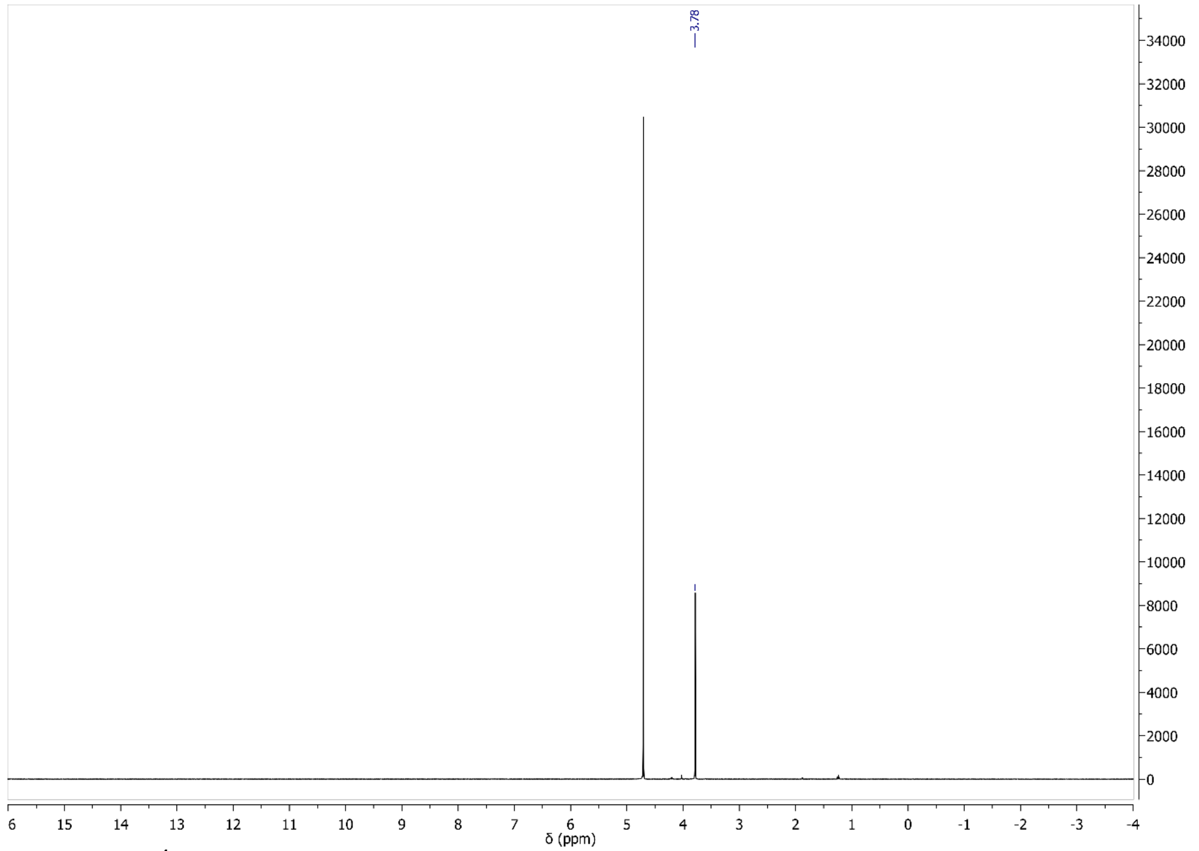


**Figure S16: ¹H-NMR (400 MHz) spectrum of *N*-oxalylglycine 5; data are consistent with the literature.** [14]

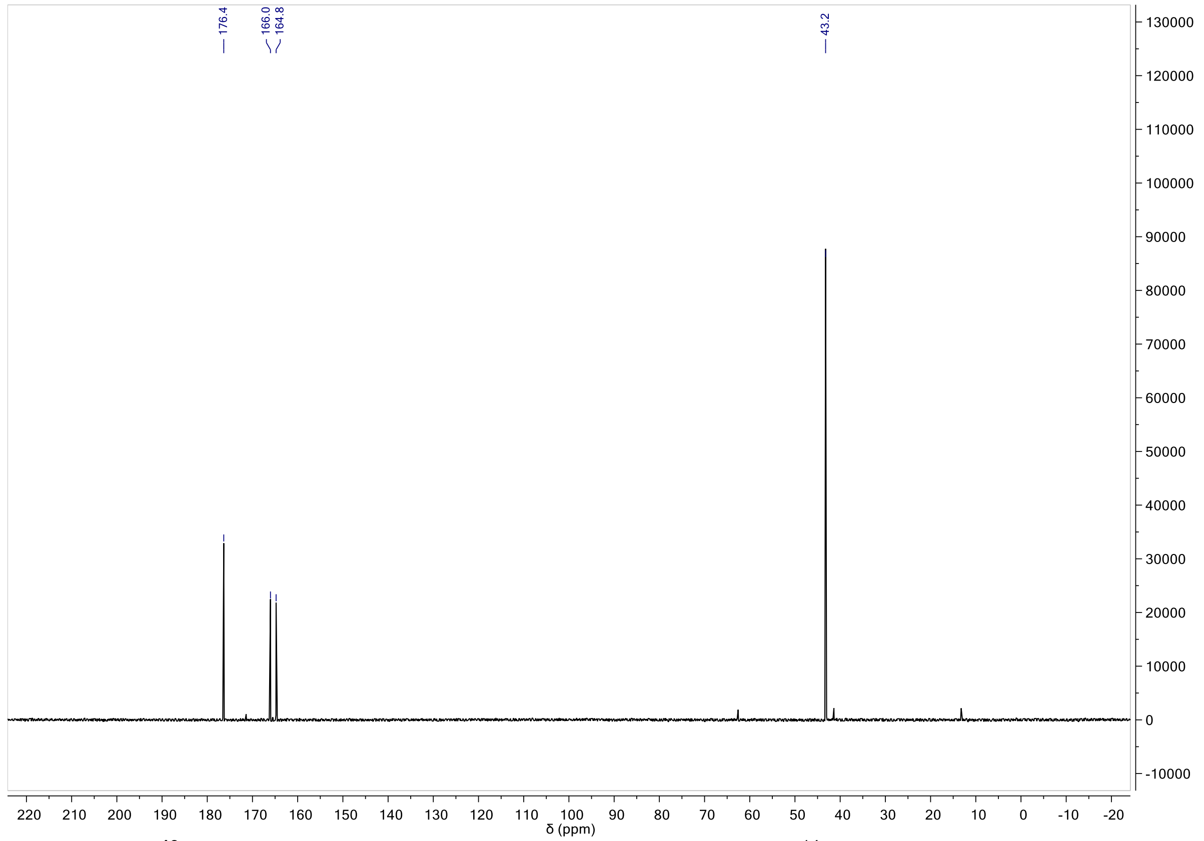


**Figure S17: ¹³C-NMR (126 MHz) spectrum of *N*-oxalylglycine 5.** [14]

# HPLC spectra

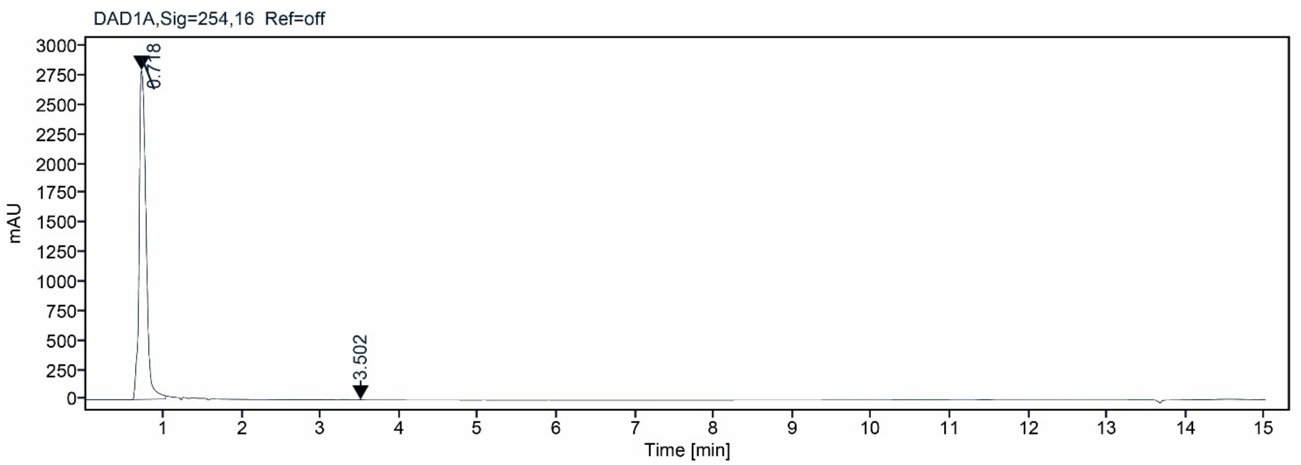

**Figure S18: HPLC trace of IOX1 3 using a UV-detector (254 nm).**

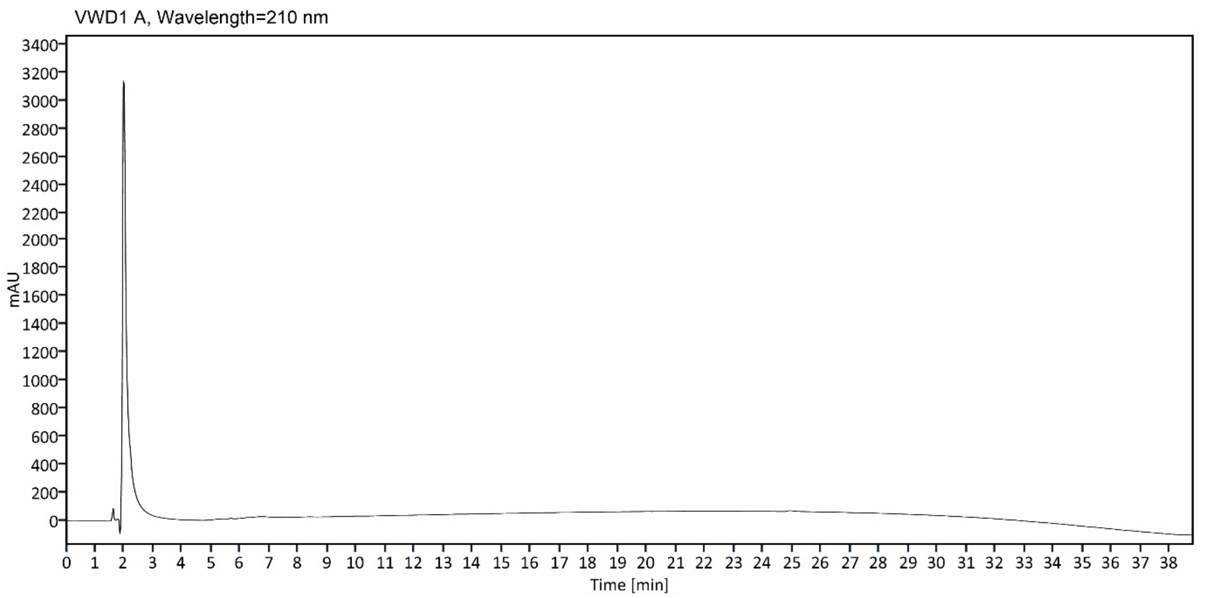

**Figure S19: HPLC trace of NOG 5 using a UV-detector (210 nm).**

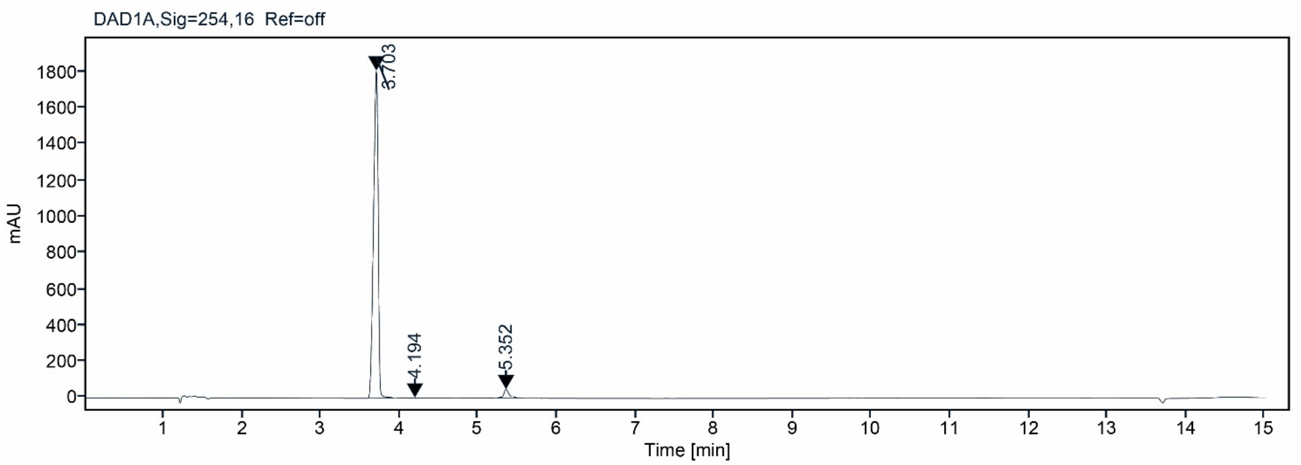

**Figure S20: HPLC trace of IOX4 8 using a UV-detector (254 nm).**

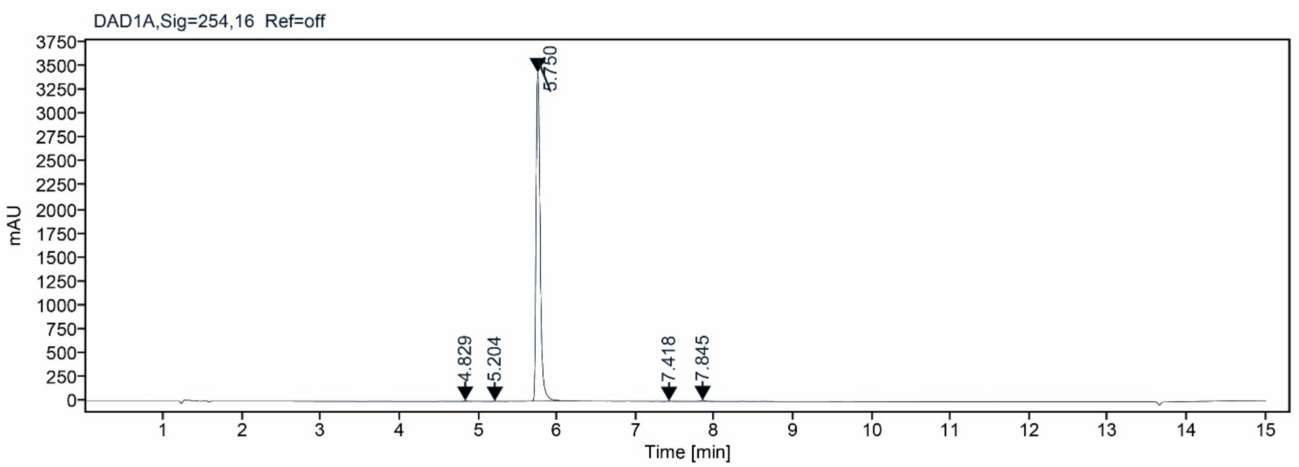


**Figure S21: HPLC trace of FG-0041 12 using a UV-detector (254 nm).**

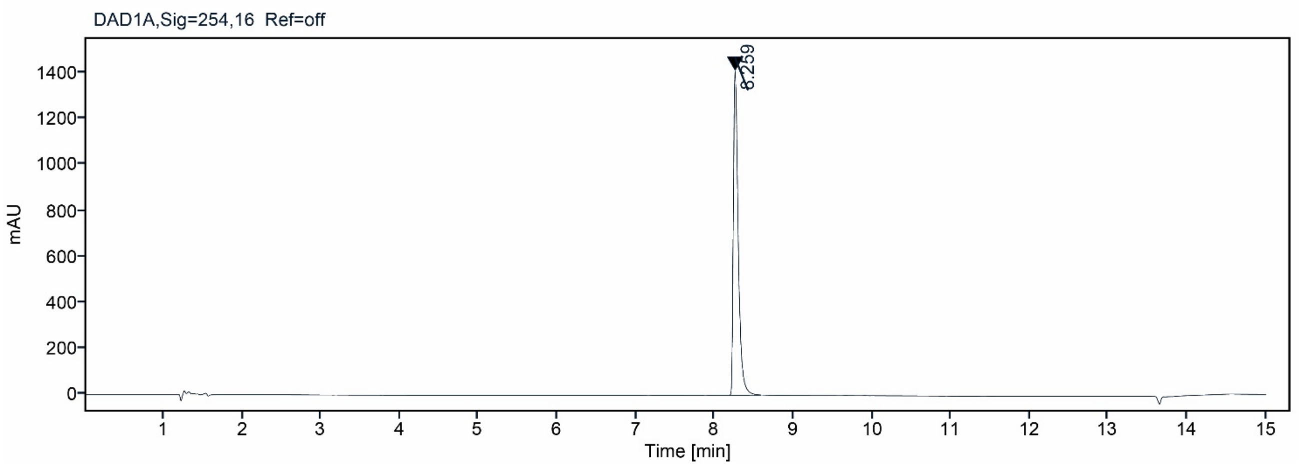


**Figure S22: HPLC trace of Vadadustat 13 using a UV-detector (254 nm).**

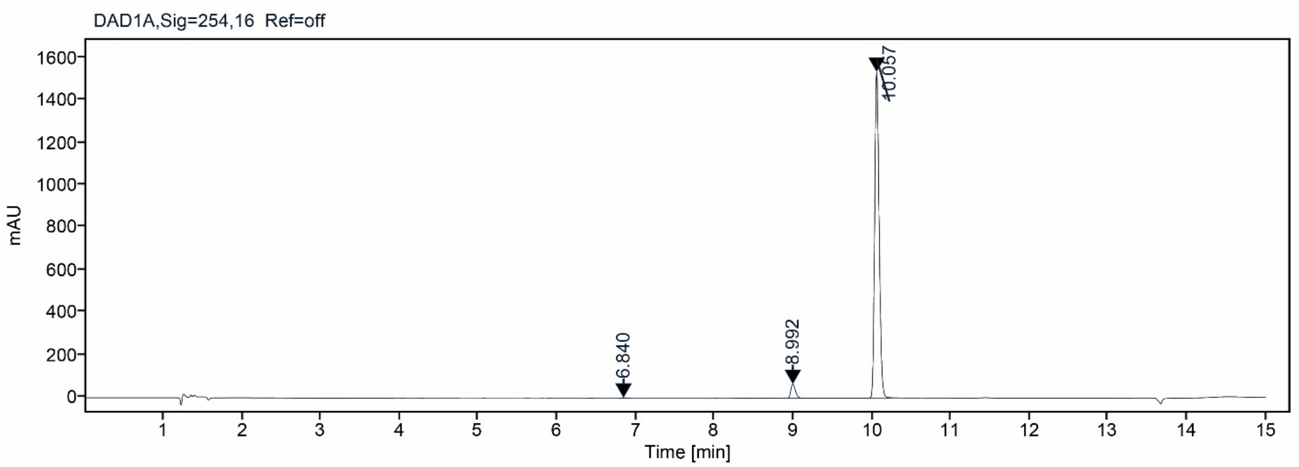


**Figure S23: HPLC trace of JIB-04 14 using a UV-detector (254 nm).**

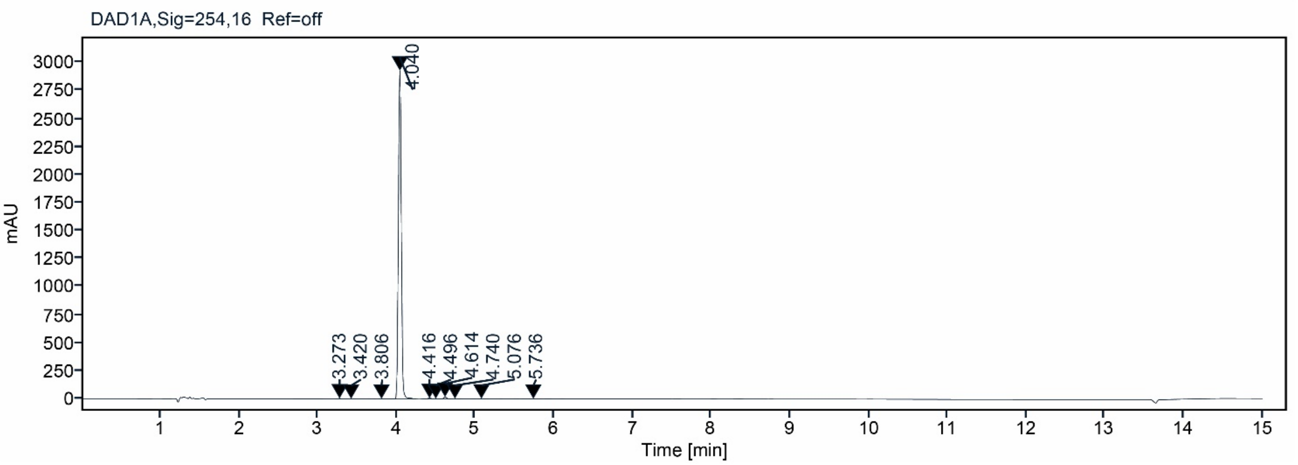

**Figure S24: HPLC trace of Panobinostat 21 using a UV-detector (254 nm).**

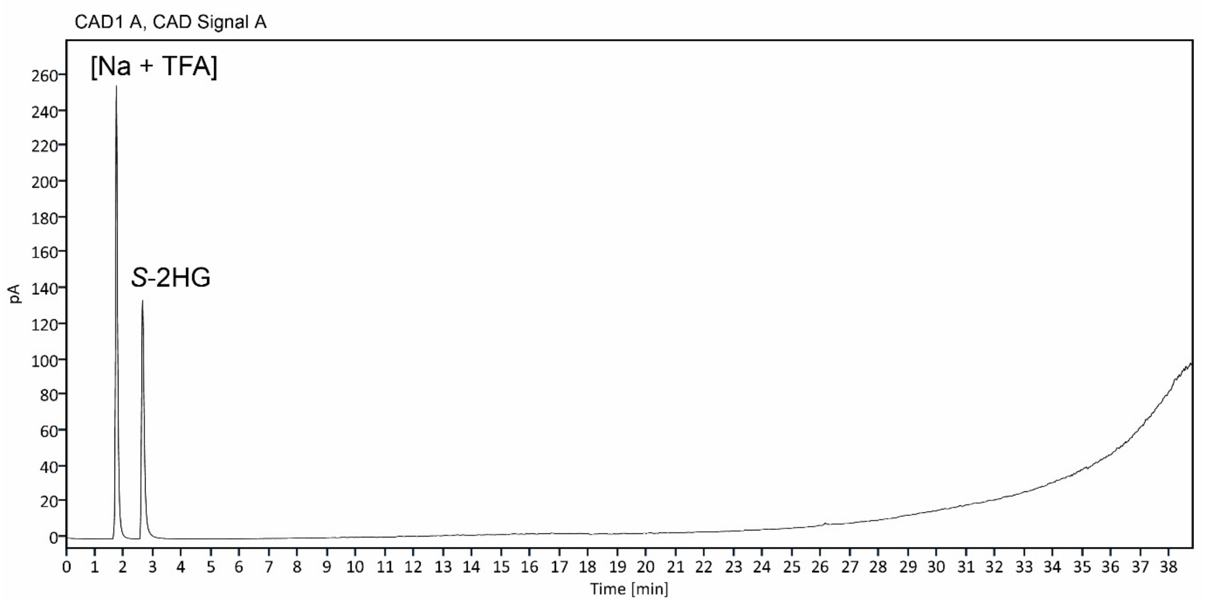


**Figure S25: HPLC trace of disodium *S*-2-hydroxyglutarate 22 using a CAD-detector.**

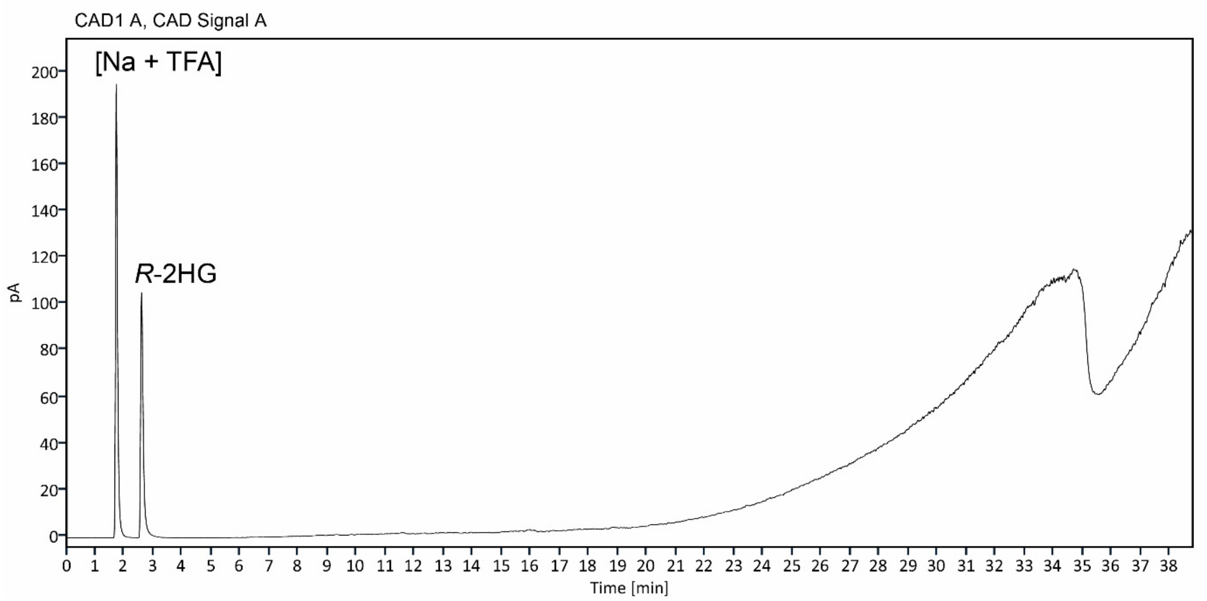


**Figure S26: HPLC trace of disodium *R*-2-hydroxyglutarate 23 using a CAD-detector.**

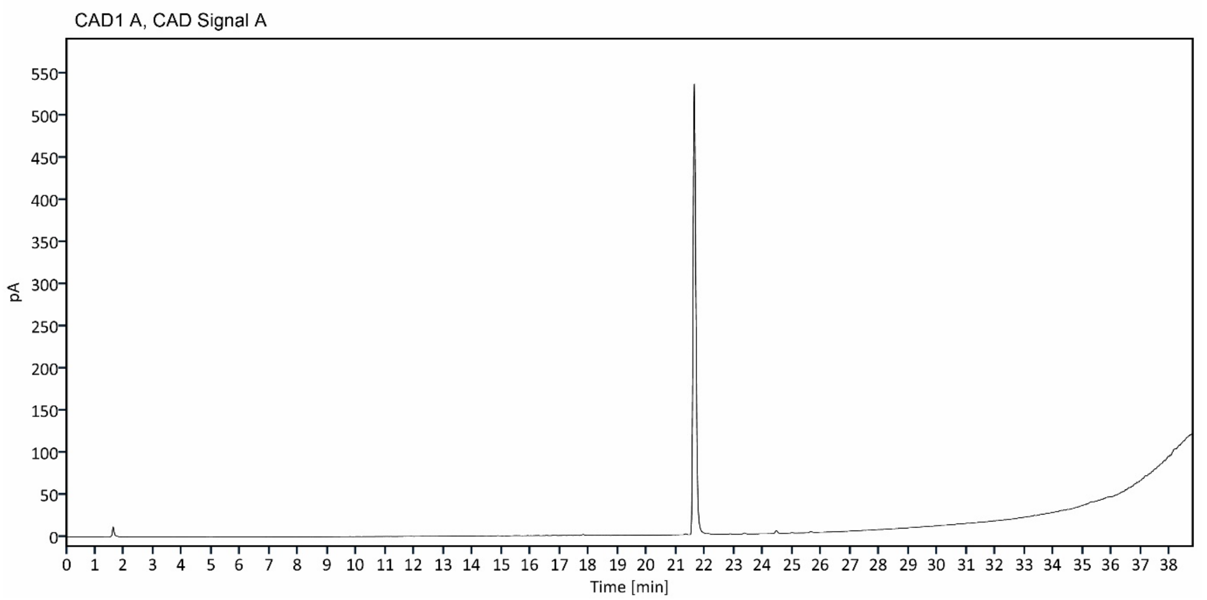


**Figure S27: HPLC trace of (*S*)-Octyl-α-hydroxyglutaric acid 36 using a CAD-detector.**

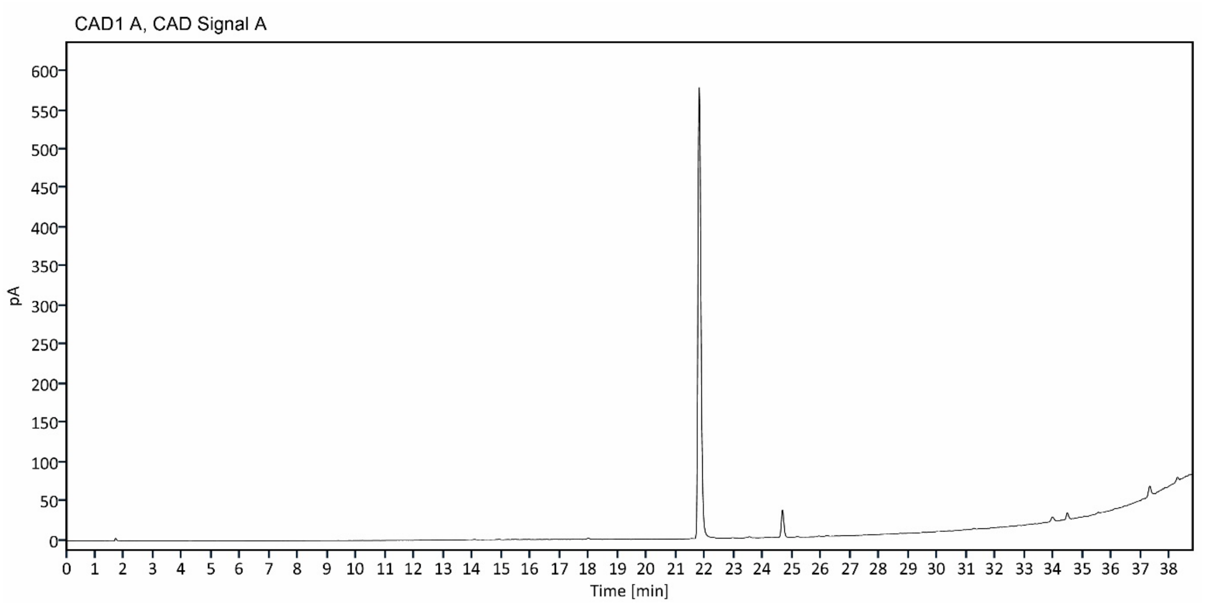


**Figure S28: HPLC trace of (*R*)-Octyl-α-hydroxyglutaric acid 37 using a CAD-detector.**

**References**


(1) Sudhamalla, B.; Dey, D.; Breski, M.; Islam, K. A Rapid Mass Spectrometric Method for the Measurement of Catalytic Activity of Ten-Eleven Translocation Enzymes. *Anal. Biochem.* **2017**, *534*, 28–35. https://doi.org/10.1016/j.ab.2017.06.011.

(2) Laukka, T.; Mariani, C. J.; Ihantola, T.; Cao, J. Z.; Hokkanen, J.; Kaelin, W. G.; Godley, L. A.; Koivunen, P. Fumarate and Succinate Regulate Expression of Hypoxia-Inducible Genes via TET Enzymes. *J. Biol. Chem.* **2016**, *291* (8), 4256–4265. https://doi.org/10.1074/jbc.M115.688762.

(3) Tarhonskaya, H.; Hardy, A. P.; Howe, E. A.; Loik, N. D.; Kramer, H. B.; McCullagh, J. S. O.; Schofield, C. J.; Flashman, E. Kinetic Investigations of the Role of Factor Inhibiting Hypoxia-Inducible Factor (FIH) as an Oxygen Sensor. *J. Biol. Chem.* **2015**, *290* (32), 19726–19742. https://doi.org/10.1074/jbc.M115.653014.

(4) Chowdhury, R.; Yeoh, K. K.; Tian, Y. M.; Hillringhaus, L.; Bagg, E. A.; Rose, N. R.; Leung, I. K. H.; Li, X. S.; Woon, E. C. Y.; Yang, M.; McDonough, M. A.; King, O. N.; Clifton, I. J.; Klose, R. J.; Claridge, T. D. W.; Ratcliffe, P. J.; Schofield, C. J.; Kawamura, A. The Oncometabolite 2-Hydroxyglutarate Inhibits Histone Lysine Demethylases. *EMBO Rep.* **2011**, *12* (5), 463–469. https://doi.org/10.1038/embor.2011.43.

(5) Hirsilä, M.; Koivunen, P.; Günzler, V.; Kivirikko, K. I.; Myllyharju, J. Characterization of the Human Prolyl 4-Hydroxylases That Modify the Hypoxia-Inducible Factor. *J. Biol. Chem.* **2003**, *278* (33), 30772–30780. https://doi.org/10.1074/jbc.M304982200.

(6) Koivunen, P.; Lee, S.; Duncan, C. G.; Lopez, G.; Lu, G.; Ramkissoon, S.; Losman, J. A.; Joensuu, P.; Bergmann, U.; Gross, S.; Travins, J.; Weiss, S.; Looper, R.; Ligon, K. L.; Verhaak, R. G. W.; Yan, H.; Kaelin, W. G. Transformation by the (R)-Enantiomer of 2-Hydroxyglutarate Linked to EGLN Activation. *Nature* **2012**, *483* (7390), 484–488. https://doi.org/10.1038/nature10898.

(7) Rydzik, A. M.; Leung, I. K. H.; Kochan, G. T.; Thalhammer, A.; Oppermann, U.; Claridge, T. D. W.; Schofield, C. J. Development and Application of a Fluoride-Detection-Based Fluorescence Assay for γ-Butyrobetaine Hydroxylase. *ChemBioChem* **2012**, *13* (11), 1559–1563. https://doi.org/10.1002/CBIC.201200256.

(8) Laukka, T.; Myllykoski, M.; Looper, R. E.; Koivunen, P. Cancer-Associated 2-Oxoglutarate Analogues Modify Histone Methylation by Inhibiting Histone Lysine Demethylases. *J. Mol. Biol.* **2018**, *430* (18), 3081–3092. https://doi.org/10.1016/j.jmb.2018.06.048.

(9) Xu, W.; Yang, H.; Liu, Y.; Yang, Y.; Wang, P. P.; Kim, S.-H.; Ito, S.; Yang, C.; Wang, P. P.; Xiao, M.-T.; Liu, L.; Jiang, W.; Liu, J.; Zhang, J.; Wang, B.; Frye, S.; Zhang, Y.; Xu, Y.; Lei, Q.; Guan, K.-L.; Zhao, S.; Xiong, Y. Oncometabolite 2-Hydroxyglutarate Is a Competitive Inhibitor of α-Ketoglutarate-Dependent Dioxygenases. *Cancer Cell* **2011**, *19* (1), 17–30. https://doi.org/10.1016/j.ccr.2010.12.014.

(10) Tarhonskaya, H.; Nowak, R. P.; Johansson, C.; Szykowska, A.; Tumber, A.; Hancock, R. L.; Lang, P.; Flashman, E.; Oppermann, U.; Schofield, C. J.; Kawamura, A. Studies on the Interaction of the Histone Demethylase KDM5B with Tricarboxylic Acid Cycle Intermediates. *J. Mol. Biol.* **2017**, *429* (19), 2895–2906. https://doi.org/10.1016/J.JMB.2017.08.007.

(11) Wang, P.; Wu, J.; Ma, S.; Zhang, L.; Yao, J.; Hoadley, K. A.; Wilkerson, M. D.; Perou, C. M.; Guan, K. L.; Ye, D.; Xiong, Y. Oncometabolite D-2-Hydroxyglutarate Inhibits ALKBH DNA Repair Enzymes and

Sensitizes IDH Mutant Cells to Alkylating Agents. *Cell Rep.* **2015**, *13* (11), 2353–2361. https://doi.org/10.1016/j.celrep.2015.11.029.

(12) Su, R.; Dong, L.; Li, C.; Nachtergaele, S.; Wunderlich, M.; Qing, Y.; Deng, X.; Wang, Y.; Weng, X.; Hu, C.; Yu, M.; Skibbe, J.; Dai, Q.; Zou, D.; Wu, T.; Yu, K.; Weng, H.; Huang, H.; Ferchen, K.; Qin, X.; Zhang, B.; Qi, J.; Sasaki, A. T.; Plas, D. R.; Bradner, J. E.; Wei, M.; Marcucci, G.; Jiang, X.; Mulloy, J. C.; Jin, J.; He, C.; Chen, J. R-2HG Exhibits Anti-Tumor Activity by Targeting FTO/M6A/MYC/CEBPA Signaling. *Cell* **2018**, *172* (1–2), 90-105.e23. https://doi.org/10.1016/j.cell.2017.11.031.

(13) Fleitz, F. J.; Lyle, T. A.; Zheng, N.; Armstrong, J. D.; Volante, R. P. Kilogram Scale Synthesis of the Pyrazinone Acetic Acid Core of an Orally Efficacious Thrombin Inhibitor. *Synth. Commun.* **2000**, *30* (17), 3171–3180. https://doi.org/10.1080/00397910008086927.

(14) Tarhonskaya, H.; Szöllössi, A.; Leung, I. K. H.; Bush, J. T.; Henry, L.; Chowdhury, R.; Iqbal, A.; Claridge, T. D. W.; Schofield, C. J.; Flashman, E. Studies on Deacetoxycephalosporin C Synthase Support a Consensus Mechanism for 2-Oxoglutarate Dependent Oxygenases. *Biochemistry* **2014**, *53* (15), 2483–2493. https://doi.org/10.1021/bi500086p.
